# Mathematical Assessment of the Roles of Vaccination and Pap Screening on the Burden of HPV and Related Cancers in Korea

**DOI:** 10.1007/s11538-025-01548-5

**Published:** 2025-12-03

**Authors:** Soyoung Park, Hyunah Lim, Abba B. Gumel

**Affiliations:** 1 Department of Mathematics, University of Maryland, College Park, 20742 MD, USA; 2 University of Maryland Institute for Health Computing, North Bethesda, 20852 MD, USA; 3 Department of Mathematics and Applied Mathematics, University of Pretoria, 0002 Pretoria, South Africa

**Keywords:** HPV, Cervical cancer, Vaccination, Korea, Reproduction number, Asymptotic stability

## Abstract

This study is based on using a novel sex-structured mathematical model to assess the effectiveness of vaccination and Pap screening against HPV and related cancers in South Korea. In addition to its disease-free equilibrium (DFE) being locally-asymptotically stable when the associated control reproduction number is less than one, the model could have one or three endemic equilibria, for a special case with negligible disease-induced mortality, if the reproduction number exceeds one. It’s shown, using a Krasnoselskii sublinearity argument, that this special case has a unique and locally-asymptotically stable endemic equilibrium, when the reproduction number is larger than one, if, additionally, the HPV vaccine is assumed to be perfect. The DFE of a simplified version of the model, which is calibrated using HPV-related cancer data in Korea, is globally-asymptotically stable when its reproduction number is less than one. Simulations of the full model showed that, although vaccine-derived herd immunity (needed for HPV elimination) cannot be achieved in Korea under the current vaccination coverage of females (of 88%), it can be achieved if, additionally, at least 65% of males are vaccinated at steady-state. While the current combined vaccination-screening strategy (termed Strategy A) will fail to eliminate HPV, extended strategies that include increased coverage of female vaccination (termed Strategy B) or additionally vaccinating boys (termed Strategy C) could lead to such elimination in Korea. The implementation of boys-only vaccination strategy induces a significant spillover benefit in reducing cervical cancer burden, which exceeds the corresponding spillover benefit achieved by implementing a girls-only vaccination strategy.

## Introduction

While cervical cancer is the fourth most frequent female cancer globally (there were 661,021 new cervical cancer cases and 348,189 fatalities globally in the year 2022 alone (Bray et al. 2024)), it is the third most frequent in the 15-44-year female age group in South Korea (there were 3,218 new cervical cancer cases and 1,018 fatalities in South Korea in the year 2020 (Bruni et al. 2023)). Cervical cancer is one of the top three cancers in females under the age of 45 globally, and the average ages for cervical cancer diagnosis and death are 53 years and 59 years, respectively (Arbyn et al. 2020). The burden of the cervical cancer disproportionately affects resource-limited countries that have lack of availability and access to screening, vaccination, and treatment measures (Bray et al. 2024).

Virtually all cervical cancer cases are caused by HPV(Human *Papillomavirus*) (Walboomers et al. 1999), which is transmitted primarily through skin-to-skin or mucosa-to-mucosa sexual contacts (Schiffman et al. 2007). Among the more than 200 serotypes of HPV, the most prevalent serotypes are HPV-16 and HPV-18, which are high-risk oncogenic types that are found in 70% of cervical cancer cases (Smith et al. 2007). The oncogenic types HPV-31/33/35/45/52/58 are the next most common serotypes that account for additional 20% of cervical cancers globally (Clifford et al. 2006). These high-risk oncogenic types cause the most pre-cancerous lesions and cancers in humans. On the other hand, HPV-6 and HPV-11 are low-risk non-oncogenic types that are responsible for 90% of genital warts (Walboomers et al. 1999). Although 70-90% of sexually active adults contract HPV at least once during their lifetime (Bosch and de Sanjose 2003; Chesson et al. 2014), 90% of HPV infections clear naturally within 2 years (Schiffman et al. 2007). HPV persists in the remaining 10%, increasing the risk of developing pre-cancerous lesions and cancer in these individuals (Bulkmans et al. 2007; Schiffman et al. 2007). Empirical studies in the US and Netherlands show that males recover faster from HPV infection than females (Giuliano et al. 2008a; Moscicki et al. 2004, 1998), and persistent infection is more common in females than in males (Van Doornum et al. 1994). This explains, in part, the higher HPV prevalence and number of HPV-related cancer cases in females than in males in South Korea (National Cancer Center Korea Central Cancer Registry 2022; Shin et al. 2004). The pre-cancer stage of the natural history of HPV is referred to as *intraepithelial neoplasia*. The *cervical intraepithelial neoplasia* (CIN) stage is divided into three grades, namely CIN1, CIN2, and CIN3, depending on the extent of the abnormalities (IARC 1995), and the risk of developing invasive cervical cancer is higher for higher grades of CIN (IARC 1995; Schiffman et al. 2007). Similarly, *intraepithelial neoplasia in males* (INM) is divided into three stages (INM1, INM2, and INM3), and leads to various cancers in males (such as anal and penile cancers) (IARC 1995).

Although the risk posed by the cervical cancer has been the major driving force of the studies on the HPV dynamics, HPV is also responsible for 88% of anal cancer, 50% of penile cancer, and 30.8% of oropharyngeal cancer cases globally, exerting burden on both females and males (de Martel et al. 2017). In South Korea, HPV-related male cancer cases tripled in the past 20 years, reaching 573 new cases in 2020 (National Cancer Center Korea Central Cancer Registry 2022). An empirical study in the US, Mexico, and Brazil reported that 65.2% of asymptomatic males in the age group of 18-70 have HPV DNA in the genital area (Giuliano et al. 2008b). Major risk factors for HPV infection include anal intercourse and contraction of other sexually-transmitted infections (STIs) (Chin-Hong Peter et al. 2004; Hernandez et al. 2008; Kyo et al. 1994; Lee et al. 2016; Marra et al. 2018), hence men who have sex with men (MSM) is one of the main risk groups of HPV infection among sexually active individuals (Chin-Hong Peter et al. 2004; Lee et al. 2016; Marra et al. 2018).

Three HPV vaccines have been approved for use in females of age 9-45 and males of age 9-26 in South Korea; the bivalent (Cervarix^®^, preventing HPV 16/18), quadrivalent (Gardasil^®^, preventing HPV 16/18/6/11), and 9-valent (Gardasil9^®^, preventing HPV 16/18/6/11/31/33/45/52/58) vaccines, which were approved in 2008, 2007, and 2016, respectively. The 9-valent vaccine (Gardasil9^®^) provides the broadest coverage of HPV types, covering 9 HPV types, and costs about $287.54 per dose in the United States in 2024 (CDC 2024) (it costs about $150 per dose in South Korea (Choi et al. 2022)). All three vaccines protect against HPV infection with over 90% efficacy if used prior to HPV exposure, and offer protection against precancerous lesions (Cheng et al. 2020). People of age 9-14 are recommended to get 2 doses, while those of age 15-26 are recommended to get 3 doses of the vaccine (World Health Organization 2022). In South Korea, the HPV National Immunization Program (NIP) began in June 2016, offering 2 doses of bivalent(Cervarix^®^) or quadrivalent(Gardasil^®^) vaccines to girls of age 12. The routine HPV immunization program in Korea was expanded to cover girls of ages 12-17 in 2022. In 2016, before the onset of the NIP, 23.1% of Korean females aged 9-59 years received the first dose of HPV vaccine (Choi et al. 2022). In 2018 (two years after the implementation of the NIP), however, 82.3% of 12-year-old girls received the first dose of HPV vaccine in Korea (Kim et al. 2018). This is still relatively low compared to the 91-98% coverage of infant vaccination series against childhood diseases covered by the NIP in South Korea (KCDC Center for Infectious Disease Control 2023).

Papanicolaou test (Pap test) is widely used as the primary method of screening for cervical cancer in countries with organized screening programs, allowing for early detection and treatment of pre-cancerous lesions, thereby reducing the incidence and mortality of cervical cancer (Devesa et al. 1989; Lim and Yoo 2019; Min et al. 2015). Some empirical studies suggest the utility of using HPV DNA test (such as, Hybrid Capture 2 and PCR-based methods) as an alternative or adjunctive screening method to the Pap test (Reid et al. 1991; Denny and Wright 2005). However, South Korea does not recommend it as a standalone test, due to its low specificity, high cost, and high variability of its performance (Lim and Yoo 2019). In contrast, Pap test (conventional Pap smear or liquid-based cytology) is implemented in South Korea, with the Korean Society for Cytopathology entrusted with managing the quality control of its nationwide implementation (Lim and Yoo 2019; Lee et al. 2008). South Korea started offering free Pap screening, as a part of the National Cancer Screening Program (NCSP), to females over age 30 every 2 years in 2002, and then extended to females over age 20 since 2016 (Shin et al. 2022). The national cervical cancer screening guideline in South Korea (2015) suggests that females over age 20 receive Pap test every 3 years, with the option of combining Pap test with HPV test in consideration of clinical decision for individual risk and preference (Min et al. 2015). The guideline also states that the screening can terminate at the age of 74 if more than 3 consecutive cytology results are negative within 10 years (Min et al. 2015). The Korean National Cancer Screening Survey reported that the Pap screening coverage in Korea during the period 2005-2020 was 61% on average (Shin et al. 2022).

Numerous mathematical models, typically in the form of a deterministic system of nonlinear differential equations, have been developed and used to study the natural history of HPV (Elbasha and Galvani 2005; Gao et al. 2021) and its progression to associated cancers (Alsaleh and Gumel 2014; Choi and Shim 2024; Elbasha et al. 2007; Goldie et al. 2007, 2008; Javame and Gumel 2016; Malik et al. 2013; Milwid et al. 2018). For instance, Elbasha and Galvani (Elbasha and Galvani 2005) examined the impact of vaccination on the dynamics of two types of HPV in a community. (Gao et al. 2021) developed and analyzed a model for HPV dynamics that includes vaccination in both the heterosexual and the MSM populations. (Alsaleh and Gumel 2014) developed and analyzed a 29-dimensional risk-structured model for HPV dynamics, computing optimal vaccination coverage level. Some mathematical models were also developed and used to investigate the cost-effectiveness of different HPV vaccination strategies (Choi and Shim 2024; Cody et al. 2021; Elbasha et al. 2007; Goldie et al. 2007, 2008). Furthermore, while (Javame and Gumel 2016) investigated the effects of Pap screening on the prevalence of HPV and related cancers, other studies (such as those in (Malik et al. 2013; Milwid et al. 2018)) examined the effects of both the vaccination and Pap screening programs against HPV and related cancers in a community.

The purpose of the current study is to extend some of the previous modeling studies to quantify the combined population-level impacts of vaccination and Pap screening against HPV and HPV-related cancers in South Korea. Specifically, the main objective of this study is to assess whether or not the existing control resources (i.e., vaccination and Pap screening, under current levels of coverage and efficacy) will be sufficient to significantly curtail the incidence and burden of HPV and HPV-related cancers in the main high-risk groups in South Korea. The effectiveness of various control strategies will also be theoretically assessed. The paper is organized as follows. The vaccination and Pap screening model for the transmission of HPV is formulated in Section 2. Detailed qualitative analysis (with respect to the existence and asymptotic stability of its disease-free and endemic equilibria) of the model are carried out in this section. In order to calibrate the model and estimate its unknown parameters, a simplified version of the model without Pap screening and with the three CIN and INM stages lumped into one compartment, is considered in Section 3. The calibration and qualitative analysis of this simplified version of the model are also described and presented in this section. Detailed global sensitivity analysis for the full model with vaccination and Pap screening (based on the values of the unknown parameters estimated from the fitting of the reduced model) is carried out in Section 4 to determine the most influential parameters of the full vaccination and Pap screening model. Numerical simulations of this model are carried out in Section 5. Concluding remarks and discussion are presented in Section 6.

## Formulation of Vaccination and Pap Screening Model

In this section, a mechanistic model for the heterosexual transmission of HPV will be developed and used to assess the combined population-level impact of routine HPV vaccination and Pap cytology screening on the incidence and burden of HPV and HPV-related cancers in South Korea. In addition to incorporating the life history features of HPV and associated dysplasia, the model to be developed stratifies the total sexually active population (which is assumed to be of age 17 or older (Nakazawa et al. 2023; Shin et al. 2004)) based on gender (male or female). Specifically, the total sexually active population of South Korea at time *t*, denoted by *N*(*t*), is split into the total number of females at time *t* (denoted by $N_f(t)$), and the total number of males at time *t* (denoted by $N_m(t)$), so that $N(t)=N_f(t)+N_m(t)$. Furthermore, the population of sexually active females is stratified into the 15 mutually exclusive compartments of unvaccinated susceptible ($S_f(t)$), vaccinated susceptible ($V_f(t)$), exposed/pre-symptomatic infectious ($E_f(t)$), symptomatic infectious ($I_f(t)$), persistently infected ($P_f(t)$) females, females having undetected CIN ($Q_{iu}$, with $i=1,2,3$ accounting for the three CIN stages or grades), detected CIN ($Q_{id}, i=1,2,3$), undetected cervical cancer ($C_u(t)$), detected cervical cancer ($C_d(t)$), females who recovered from cervical cancer ($R_f^C(t)$), and females who recovered from HPV infection before developing cervical cancer ($R_f(t)$). Hence,

$$\begin{aligned} N_f(t)= &  S_f(t)+V_f(t)+E_f(t)+I_f(t)+P_f(t)\\ &  +{\sum _{i=1}^3\left( Q_{iu}(t)+Q_{id}(t)\right) }+C_u(t)+C_d(t)+R_f^C(t)+R_f(t). \end{aligned}$$

Similarly, the total population of sexually active males is split into the 11 mutually exclusive compartments of unvaccinated susceptible ($$S_m(t)$$), vaccinated susceptible ($$V_m(t)$$), exposed/pre-symptomatic infectious ($$E_m(t)$$), symptomatic infectious ($$I_m(t)$$), persistently infected ($$P_m(t)$$) males, males having INM ($$J_{im},$$ with $$ i=1,2,3$$ accounting for the three INM stages), males having HPV-related cancers ($$C_m$$), males who recovered from HPV-related cancers ($$R_m^C$$), and males who recovered from HPV infection before developing HPV-related cancers ($$R_m$$), so that

$$\begin{aligned}N_m(t)=S_m(t) + V_m(t) + E_m(t) + I_m(t) + P_m(t) + \sum _{i=1}^3 J_{im}(t) + C_m(t) + R_m^C(t) + R_m(t).\end{aligned}$$

The population of sexually active unvaccinated susceptible females ($S_f$) and males ($S_m$) acquire HPV infection at the rate $\lambda _{mf}$ and $\lambda _{fm}$, respectively, given by:

2.1 $$\begin{aligned} \lambda _{mf}&= \left( \beta _{mf} c_{mf}\right) \left[ \frac{I_m + \eta _m E_m + \theta _m [P_m + J_{1m} + \kappa _m(J_{2m} + J_{3m})]}{N_m}\right] , \\ \lambda _{fm}&= \left( \beta _{fm} c_{fm}\right) \left[ \frac{I_f + \eta _{f}E_f + \theta _{f} [P_f + Q_{1u} + \kappa _{f}(Q_{2u} + Q_{3u}) + \nu \{Q_{1d} + \kappa _{fd}(Q_{2d} + Q_{3d})\}]}{N_f}\right] . \nonumber \end{aligned}$$

In (2.1), $\beta _{mf}$ is the probability of HPV transmission from symptomatic infectious males to unvaccinated susceptible females per partnership, and $c_{mf}$ is the average number of male sexual partners a sexually active female has per unit time (for this study, time is measured in years). The modification parameter $0\le \eta _m < 1$ accounts for the assumed reduced transmission probability of HPV by exposed/pre-symptomatic infectious males, in comparison to the transmission probability of symptomatic infectious males. Furthermore, $0 \le \theta _m < 1$ measures the assumed reduction of infectiousness of males who are at, or past, the persistent HPV infection stage, in comparison to the infectiousness of symptomatic infectious males, and $\kappa _m \ge 1$ is the modification parameter accounting for the assumed increased probability of HPV transmission from males with INM2 and INM3, in comparison to males with INM1 and persistent HPV infection. Similarly, $\beta _{fm}$ is the transmission probability from symptomatic infectious females to unvaccinated susceptible males per partnership, and $c_{fm}$ represents the average number of female sexual partners a male has per unit time. The modification parameter $0 \le \eta _{f} < 1$ accounts for the assumed reduced probability of HPV transmission by exposed/pre-symptomatic infectious females, in comparison to the HPV transmission by symptomatic infectious females. Furthermore, the parameter $0 \le \theta _{f} < 1$ represents the assumed reduction of the infectiousness of females that are at, or past, the persistent HPV infection stage, in comparison to the infectiousness of symptomatic infectious females, and $\kappa _{f} \ge 1$ accounts for the assumed increased probability of HPV transmission by females with undetected CIN2 and CIN3, in comparison to females with undetected CIN1 and persistent HPV infection. The parameter $0 \le \nu < 1$ accounts for the assumed reduction in the number of sexual contacts by females detected with pre-cancerous lesions, in comparison to females with undetected pre-cancerous lesions and persistent infection. Finally, the parameter $\kappa _{fd} \ge 1$ represents the assumed increase in the probability of HPV transmission by females with detected CIN2 and CIN3, in comparison to females with detected CIN1.

It should be stated that, since the model to be developed in this section only considers heterosexual transmission of HPV (i.e., transmission only occurs *via* sexual contacts between males and females), the following conservation law of total number of sexual partnerships per unit time must hold (i.e., the total number of sexual partnerships sexually active females have (with males) in the community must balance those by sexually active males (with females)):

2.2 $$\begin{aligned} c_{mf}N_f(t) = c_{fm}(t)N_m(t). \end{aligned}$$

It is assumed that females can always find enough number of male partners in the community to have sexual contacts with, so $c_{mf}$ is set to be a constant (this assumption is justified by the fact that males generally have a higher desired number of female sexual partners than females (Fenigstein and Preston 2007), and tend to overreport their lifelong number of female sexual partners (Brewer et al. 2000), suggesting a higher demand among males for multiple female sexual partners. In addition, an empirical study using the famous online dating app, Tinder, shows that women attain a larger number of men matches in a short time (Tyson et al. 2016), which supports the claim that it is easier for women to find sexual partners than men). Hence, based on all this, the parameter $c_{fm}$ (for the average number of female sexual partners a male has) can be computed from (2.2) as (i.e., the rate $c_{fm}$ depends on the ratio of the total sub-populations of males and females at each time)

2.3 $$\begin{aligned} c_{fm}(t) = \frac{c_{mf}N_f(t)}{N_m(t)}. \end{aligned}$$

Using (2.2) (with (2.3)), it follows that the expressions for the forces of infection, $\lambda _{mf}$ and $\lambda _{fm}$, given in (2.1), can now be re-written, respectively, as:

2.4 $$\begin{aligned} \lambda _{mf}&= \left( \beta _{mf} c_{mf}\right) \left[ \frac{I_m + \eta _m E_m + \theta _m [P_m + J_{1m} + \kappa _m(J_{2m} + J_{3m})]}{N_m}\right] , ~\textrm{and}\\ \lambda _{fm}&= \left( \beta _{fm} c_{mf}\right) \left[ \frac{I_f + \eta _{f}E_f + \theta _{f} [P_f + Q_{1u} + \kappa _{f}(Q_{2u} + Q_{3u}) + \nu \{Q_{1d} + \kappa _{fd}(Q_{2d} + Q_{3d})\}]}{N_m}\right] . \nonumber \end{aligned}$$

Based on the above formulation, derivations (for the conservation law (2.3) and forces of infection (2.4)), and assumptions, the sex-structured model for the heterosexual transmission of HPV and related cancers, in the presence of HPV vaccination and Pap screening, in South Korea is given by the following deterministic system of nonlinear differential equations:

$$\begin{aligned}&{Females} \left\{ \begin{array}{lcl} \dot{S}_f & =& (1-q_f)\pi _f - (\lambda _{mf} + \xi _f + \mu ) S_f, \vspace{2mm} \\ \dot{V}_f & =& q_f \pi _f + \xi _f S_f- (1-\varepsilon _v) \lambda _{mf} V_f - \mu V_f, \vspace{2mm} \\ \dot{E}_f & =& \lambda _{mf} S_f + (1-\varepsilon _v) \lambda _{mf} V_f - (\sigma _f + \mu ) E_f, \vspace{2mm} \\ \dot{I}_f & =& \sigma _f E_f - (\psi _f + \mu ) I_f, \vspace{2mm}\\ \dot{P}_f & =& (1-b_f)\psi _f I_f - (\alpha _f + \mu )P_f, \vspace{2mm}\\ \dot{Q}_{1u} & =& (1-k_f) \alpha _f P_f + h_{u2}y_f Q_{2u} -(g_f +\mu ) Q_{1u}, \vspace{2mm}\\ \dot{Q}_{2u} & =& (1-d_{u1}-d_{u3}) g_f Q_{1u} +q_{u2}z_fQ_{3u} -(y_f +\mu ) Q_{2u}, \vspace{2mm}\\ \dot{Q}_{3u} & =& (1-h_{u1}-h_{u2}-h_{u3})y_f Q_{2u}-(z_f+\mu ) Q_{3u}, \vspace{2mm}\\ \dot{Q}_{1d} & =& d_{u3}g_f Q_{1u} + h_{d2} \zeta _2 Q_{2d} -(\zeta _1 +\mu ) Q_{1d}, \vspace{2mm}\\ \dot{Q}_{2d} & =& h_{u3}y_f Q_{2u} +(1-d_{d1})\zeta _1 Q_{1d} + q_{d2}\zeta _3 Q_{3d} -(\zeta _2+\mu ) Q_{2d}, \vspace{2mm}\\ \dot{Q}_{3d} & =& q_{u3} z_f Q_{3u} + (1-h_{d1}-h_{d2})\zeta _2 Q_{2d} -(\zeta _3+\mu ) Q_{3d}, \vspace{2mm}\\ \dot{C}_u & =& (1-q_{u1}-q_{u2}-q_{u3})z_f Q_{3u} -(w_f + r_u + \delta + \mu ) C_u, \vspace{2mm} \\ \dot{C}_d & =& (1-q_{d1}-q_{d2})\zeta _3 Q_{3d} + w_f C_u -(r_d + \delta _d + \mu ) C_d, \vspace{2mm} \\ \dot{R}_f^C & =& r_u C_u + r_d C_d -\mu R_f^C, \vspace{2mm}\\ \dot{R}_f & =& b_f \psi _f I_f + k_f \alpha _f P_f + d_{u1}g_f Q_{1u} + h_{u1}y_f Q_{2u} + q_{u1}z_f Q_{3u} + d_{d1} \zeta _1 Q_{1d} \\  & & + h_{d1}\zeta _2 Q_{2d} + q_{d1} \zeta _3 Q_{3d} -\mu R_f, \end{array} \right. \\\\ \end{aligned}$$

2.5 $$\begin{aligned} \begin{aligned}&{Males} \left\{ \begin{array}{lcl} \dot{S}_m & =& (1-q_m)\pi _{m} - (\lambda _{fm} +\xi _m +\mu ) S_m, \vspace{2mm}\\ \dot{V}_m & =& q_m\pi _m +\xi _mS_m - (1-\varepsilon _v) \lambda _{fm} V_m - \mu V_m, \vspace{2mm}\\ \dot{E}_m & =& \lambda _{fm} S_m+ (1-\varepsilon _v) \lambda _{fm} V_m - (\sigma _m +\mu )E_m, \vspace{2mm} \\ \dot{I}_m & =& \sigma _m E_m - (\psi _m + \mu ) I_m, \vspace{2mm}\\ \dot{P}_m & =& (1-b_m)\psi _m I_m - (\alpha _m + \mu )P_m, \vspace{2mm}\\ \dot{J}_{1m} & =& (1-k_m)\alpha _m P_m + h_{m2}y_m J_{2m}- (g_m+\mu )J_{1m}, \vspace{2mm} \\ \dot{J}_{2m} & =& (1-d_m)g_m J_{1m} + q_{m2}z_m J_{3m}- (y_m +\mu )J_{2m}, \vspace{2mm} \\ \dot{J}_{3m} & =& (1-h_{m1}-h_{m2})y_m J_{2m}-(z_m+\mu ) J_{3m}, \vspace{2mm} \\ \dot{C}_m & =& (1-q_{m1}-q_{m2})z_m J_{3m} -(r_m +\mu ) C_m, \vspace{2mm} \\ \dot{R}_m^C & =& r_m C_m -\mu R_m^C, \vspace{2mm}\\ \dot{R}_m & =& b_m \psi _m I_m + k_m \alpha _m P_m + d_mg_m J_{1m} + h_{m1}y_m J_{2m} + q_{m1}z_m J_{3m}- \mu R_m. \end{array} \right. \end{aligned} \end{aligned}$$

In the model (2.5), the population of sexually active unvaccinated susceptible females ($S_f$) is generated by the recruitment of new sexually active individuals into the community at a rate $(1-q_f)\pi _f$ (where the proportion $q_f$ is vaccinated against HPV), and is reduced by the acquisition of HPV infection at the rate $\lambda _{mf}$, administration of the catch-up HPV vaccination (at a rate $\xi _f$), and natural death (at a rate $\mu$; individuals in all epidemiological class are assumed to die naturally at this rate). The population of sexually active vaccinated susceptible females ($V_f$) is increased by the cohort (at the rate $q_f\pi _f$) and catch-up (at the rate $\xi _f$) vaccination. Breakthrough infection occurs at the rate $(1-\varepsilon _v)\lambda _{mf}$, where $0 \le \varepsilon _v \le 1$ is the efficacy of the vaccine to protect against the acquisition of HPV infection. Exposed/pre-symptomatic infectious females (i.e., those in the $E_f$ class) develop clinical symptoms of HPV at a rate $\sigma _f$. Symptomatic infectious females (i.e., those in the $I_f$ class) leave this class at a rate $\psi _f$, a proportion, $b_f$, of these individuals recover (at a rate $b_f\psi _f$), and the remaining proportion, $1-b_f$, progress to persistent HPV infection (at a rate $(1-b_f)\psi _f$). Females with persistent HPV infection (i.e., those in the $P_f$ class) leave this class at a rate $\alpha _f$, a proportion, $k_f$, of these individuals recover (at a rate $k_f\alpha _f$), and the remaining proportion, $1-k_f$, move to the undetected CIN1 class (at a rate $(1-k_f)\alpha _f$).

Females with undetected CIN1 (i.e., those in the $Q_{1u}$ class) transition out of this class at a rate $g_f$, and a proportion, $d_{u1}$, of these individuals recover (at a rate $d_{u1}g_f$), another proportion, $d_{u3}$, are detected *via* Pap screening and move to the detected CIN1 class (at a rate $d_{u3}g_f$), and, finally, the remaining proportion, $1-(d_{u1}+d_{u3})$, progress to the undetected CIN2 class (at a rate $(1-d_{u1}-d_{u3})g_f$). Females with undetected CIN2 (i.e., those in the $Q_{2u}$ class) leave this class at a rate $y_f$. A proportion, $h_{u1}$, of these individuals recover (at a rate $h_{u1}y_f$), another proportion, $h_{u2}$, regress to the undetected CIN1 class (at a rate $h_{u2}y_f$), a proportion, $h_{u3}$, are detected *via* Pap screening and move to the detected CIN2 class (at a rate $h_{u3}y_f$), and the remaining proportion, $1-(h_{u1}+h_{u2}+h_{u3})$, progress to the undetected CIN3 class (at a rate $(1-h_{u1}-h_{u2}-h_{u3})y_f$). Females with undetected CIN3 (i.e., those in the $Q_{3u}$ class) leave this class at a rate $z_f$. A proportion, $q_{u1}$, of these individuals recover (at a rate $q_{u1}z_f$), a proportion, $q_{u2}$, regress to the undetected CIN2 class (at a rate $q_{u2}z_f$), a proportion, $q_{u3}$, are detected *via* Pap screening and move to the detected CIN3 class (at a rate $q_{u3}z_f$), and the remaining proportion, $1-(q_{u1}+q_{u2}+q_{u3})$, progress to the undetected cervical cancer class (at a rate $(1-q_{u1}-q_{u2}-q_{u3})z_f$). Females with detected CIN1 (i.e., those in the $Q_{1d}$ class) transition out of this class at a rate $\eta _1$. A proportion, $d_{d1}$, of these individuals recover (at a rate $d_{d1}\eta _1$), and the remaining proportion, $1-d_{d1}$, progress to the detected CIN2 class (at a rate $(1-d_{d1})\eta _1$). Females with detected CIN2 (i.e., those in the $Q_{2d}$ class) leave this class at a rate $\eta _2$. A proportion, $h_{d1}$, of these individuals recover (at a rate $h_{d1}\eta _2$), a proportion, $h_{d2}$, regress to the detected CIN1 class (at a rate $h_{d2}\eta _2$), and the remaining proportion, $1-(h_{d1}+h_{d2})$, progress to the detected CIN3 class (at a rate $(1-h_{d1}-h_{d2})\eta _2$). Females with detected CIN3 (i.e., those in the $Q_{3d}$ class) leave this class at a rate $\eta _3$. A proportion, $q_{d1}$, of these individuals recover (at a rate $q_{d1}\eta _3$), a proportion, $q_{d2}$, regress to the detected CIN2 class (at a rate $q_{d2}\eta _3$), and the remaining proportion, $1-(q_{d1}+q_{d2})$, progress to the detected cervical cancer class (at a rate $(1-q_{d1}-q_{d2})\eta _3$). Females with undetected cervical cancer (i.e., those in the $C_u$ class) recover from cancer at a rate $r_u$, are detected *via* Pap screening and move to the detected cervical cancer class at a rate $w_f$, or die of cancer at a rate $\delta$. Females with detected cervical cancer (i.e., those in the $C_d$ class) recover from cervical cancer at a rate $r_d$, or die of cancer at a rate $\delta _d$.

The population of sexually active unvaccinated susceptible males ($S_m$) is increased by recruitment at a rate $(1-q_m)\pi _m$ (where the proportion $q_m$ is vaccinated against HPV), and is decreased by the acquisition of HPV infection (at the rate $\lambda _{fm}$), catch-up vaccination (at a rate $\xi _m$), and natural death (at the rate $\mu$). The population of vaccinated susceptible males ($V_m$) is increased by the cohort (at the rate $q_m\pi _m$) and catch-up (at the rate $\xi _m$) vaccination. Breakthrough infection occurs at the rate $(1-\epsilon _v)\lambda _{mf}$. Exposed/pre-symptomatic infectious males (i.e., those in the $E_m$ class) develop clinical symptoms of HPV at a rate $\sigma _m$. Symptomatic infectious males (i.e., those in the $I_m$ class) leave this class at a rate $\psi _m$. A proportion, $b_m$, of these individuals recover (at a rate $b_m\psi _m$), and the remaining proportion, $1-b_m$, progress to persistent infection (at a rate $(1-b_m)\psi _m$). Males with persistent HPV infection (i.e., those in the $P_m$ class) leave this class at a rate $\alpha _m$. A proportion, $k_m$, of these individuals recover (at a rate $k_m\alpha _m$), and the remaining proportion, $1-k_m$, move to the undetected CIN1 class (at a rate $(1-k_m)\alpha _m$). Males with INM1 (i.e., those in the $J_{1m}$ class) transition out of this class at a rate $g_m$. A proportion, $d_{a}$, of these individuals recover (at a rate $d_mg_m$), and the remaining proportion, $1-d_m$, progress to the INM2 class (at a rate $(1-d_m)g_m$). Males with INM2 (i.e., those in the $J_{2m}$ class) leave this class at a rate $y_m$. A proportion, $h_{m1}$, of these individuals recover (at a rate $h_{m1}y_m$), a proportion, $h_{m2}$, regress to the INM1 class (at a rate $h_{m2}y_m$), and the remaining proportion, $1-(h_{m1}+h_{m2})$, progress to the INM3 class (at a rate $(1-h_{m1}-h_{m2})y_m$). Males with INM3 (i.e., those in the $J_{3m}$ class) leave this class at a rate $z_m$. A proportion, $q_{m1}$, of these individuals recover (at a rate $q_{m1}z_m$), a proportion, $q_{m2}$, regress to the INM2 class (at a rate $q_{m2}z_m$), and the remaining proportion, $1-(q_{m1}+q_{m2})$, progress to the HPV-related cancer class (at a rate $(1-q_{m1}-q_{m2})z_m$). Males with HPV-related cancer (i.e., those in the $C_m$ class) recover from cancer at a rate $r_m$.

The main assumptions made in the formulation of the model (2.5) include (*inter alia*):

(i) Homogeneous mixing (i.e., every sexually active heterosexual individual is equally likely to mix with, and have sexual contact, with every other sexually active heterosexual individual)
(ii) Large population size (so that random or stochastic fluctuations can be ignored)
(iii) Exponentially-distributed waiting times in each epidemiological compartment
(iv) No prior immunity against HPV infection (i.e., no maternal immunity) and recovery from previous HPV infection induces permanent immunity against the acquisition of future infections (Elbasha and Galvani 2005; Malik et al. 2013)
(v) Immunity derived from HPV vaccine does not wane during the period of consideration for the current study (i.e., during the simulation period considered in this study) (Elbasha 2007)
(vi) Individuals in the detected CIN stages ($Q_{id}; ~i=1,2,3$) are females who got detected for their pre-cancerous lesions during the regular Pap screening performed in the population (South Korea) every 2 years (Shin et al. 2022)
(vii) Males do not die of HPV-related cancers (although males die of anal, penile, or oropharyngeal cancers, the rates of occurrence of these deaths are quite negligible compared to deaths due to HPV-related cancers in females (Bray et al. 2024; de Martel et al. 2017)).

The model (2.5) is an extension of many HPV models in the literature, such as those in (Alsaleh and Gumel 2014; Elbasha 2007; Elbasha and Galvani 2005; Javame and Gumel 2016; Malik et al. 2013; Milwid et al. 2018) by (*inter alia*):

- including both cohort and catch-up vaccination for sexually active unvaccinated susceptible females and males (only cohort vaccination is considered in (Alsaleh and Gumel 2014; Elbasha 2007; Elbasha and Galvani 2005; Javame and Gumel 2016; Malik et al. 2013))
- incorporating multiple grades of pre-cancerous lesions (CIN for females, INM for heterosexual males) in the HPV dynamics (pre-cancerous stages are not included in (Elbasha 2007; Elbasha and Galvani 2005), and only CIN stages for females are included in (Malik et al. 2013))
- allowing for regression from grade *i* to grade $i-1$ ($i=2$ and 3) of the pre-cancerous CIN and INM stages for females and males, respectively (regression of detected pre-cancerous lesions are not included in (Javame and Gumel 2016), regression from grade 3 to grade 2 is not included in (Malik et al. 2013), and regression of undetected pre-cancerous lesions from grade 3 to grade 2 are not included in (Milwid et al. 2018))
- allowing for HPV transmission by individuals in the pre-cancerous stages (this is not included in (Alsaleh and Gumel 2014; Malik et al. 2013), and HPV transmission by individuals with CIN3 is not included in (Milwid et al. 2018))
- including a class of exposed (pre-symptomatic infectious) individuals (females and males), and allowing for HPV transmission by these individuals (no exposed class is included in (Elbasha 2007; Elbasha and Galvani 2005; Malik et al. 2013; Milwid et al. 2018))

**Fig. 1:**
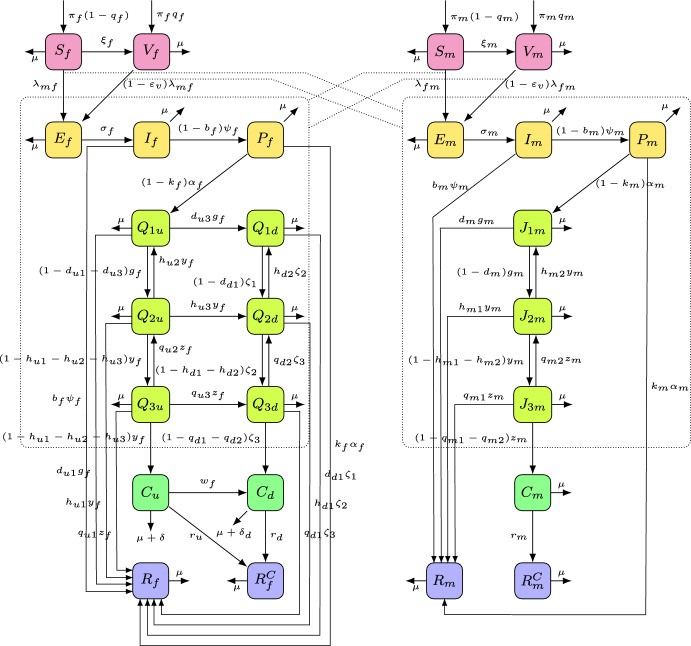
Schematic diagram of the model (2.5).

A flow diagram of the model (2.5) is depicted in Figure 1 (the state variables and parameters of the model are described in Tables 1-3).

**Table 1:** Description of the state variables of the model (2.5).

State variable ($i = 1,2,3$)	Description ($i = 1,2,3$)
$S_f (S_{m})$	Number of unvaccinated susceptible females (males)
$V_f (V_{m})$	Number of vaccinated susceptible females (males)
$E_f (E_{m})$	Number of exposed/pre-symptomatic infectious females (males)
$I_f (I_{m})$	Number of symptomatic infectious females (males)
$P_f (P_{m})$	Number of females (males) with persistent infection
$Q_{iu}$	Number of females having undetected CIN of grade *i*
$Q_{id}$	Number of females having detected CIN of grade *i*
$J_{im}$	Number of males having INM of grade *i*
$C_{u}$	Number of females having undetected cervical cancer
$C_{d}$	Number of females having detected cervical cancer
$C_{m}$	Number of males having HPV-related cancers
$R_f^C (R_{m}^C)$	Number of females (males) recovered from cancer
$R_f (R_{m})$	Number of females (males) recovered from HPV infection before developing cancer

**Table 2:** Description of the parameters of the model (2.5).

Parameter	Description ($i =1,2,3$)
$\pi _f (\pi _m)$	Recruitment rate of sexually active females (males)
$q_f (q_m)$	Proportion of new sexually active females (males) vaccinated
$\xi _f (\xi _m)$	Catch-up vaccination rate of females (males)
$1/\mu$	Average duration of sexual activity
$c_{mf} (c_{fm})$	Average number of sexual partners of females (males) per year
$\beta _{mf} (\beta _{fm})$	Transmission probability from infected males (females) to susceptible females (males) per partnership
$\eta _{f} (\eta _m)$	Modification parameter for infectiousness of $E_f (E_m)$ in comparison to $I_f (I_m)$
$\theta _{f} (\theta _m)$	Modification parameter for infectiousness of $P_f, Q_{iu}, Q_{id} (P_m, J_{im})$ in comparison to $I_f (I_m)$
$\kappa _{f} (\kappa _m)$	Modification parameter for infectiousness of $Q_{2u}, Q_{3u} (J_{2m}, J_{3m})$ in comparison to $P_f, Q_{1u} (P_m, J_{1m})$
$\nu$	Modification parameter for infectiousness of $Q_{1d}, Q_{2d}, Q_{3d}$ in comparison to $P_f, Q_{iu}$
$\kappa _{fd}$	Modification parameter for infectiousness of $Q_{2d}, Q_{3d}$ in comparison to $Q_{1d}$
$\varepsilon _v$	Efficacy of bivalent Cervarix^®^ (quadrivalent Gardasil^®^) vaccine
$\sigma _f (\sigma _m)$	Rate of symptom development of females (males)
$\psi _f (\psi _m)$	Rate at which females (males) leave the symptomatic infectious class
$\alpha _f (\alpha _m)$	Rate at which females (males) leave the persistent infection class
$g_f, y_f, z_f$	Rate at which females leave the undetected CIN1, CIN2, CIN3 class
$\zeta _1, \zeta _2, \zeta _3$	Rate at which females leave the detected CIN1, CIN2, CIN3 class
$g_m, y_m, z_m$	Rate at which males leave the INM1, INM2, INM3 class
$w_f$	Detection rate of cancer in females
$r_u (r_d)$	Recovery rate of undetected (detected) cancer for females
$r_m$	Recovery rate of cancer for males
$\delta (\delta _d)$	Mortality rate for females with undetected (detected) cervical cancer

**Table 3:** Epidemiological proportions associated with some of the parameters of the model (2.5) described in Table 2.

Parameter	Description
$b_f (b_m)$	Proportion of symptomatic infectious females (males) that recover
$1-b_f$	Proportion of symptomatic infectious females that progress to persistent infection
$1-b_m$	Proportion of symptomatic infectious males that progress to persistent infection
$k_f (k_m)$	Proportion of persistently infected females (males) that recover
$1-k_f$	Proportion of persistently infected females that progress to undetected CIN1
$1-k_m$	Proportion of persistently infected males that progress to INM1
$d_{u1}$	Proportion of females with undetected CIN1 that recover naturally
$d_{u3}$	Proportion of females with undetected CIN1 that get detected for CIN
$1-d_{u1}-d_{u3}$	Proportion of females with undetected CIN1 that progress to undetected CIN2
$h_{u1}$	Proportion of females with undetected CIN2 that recover naturally
$h_{u2}$	Proportion of females with undetected CIN2 that regress to undetected CIN1
$h_{u3}$	Proportion of females with undetected CIN2 that get detected for CIN
$1-h_{u1}-h_{u2}-h_{u3}$	Proportion of females with undetected CIN2 that progress to undetected CIN3
$q_{u1}$	Proportion of females with undetected CIN3 that recover naturally
$q_{u2}$	Proportion of females with undetected CIN3 that regress to undetected CIN2
$q_{u3}$	Proportion of females with undetected CIN3 that get detected for CIN
$1-q_{u1}-q_{u2}-q_{u3}$	Proportion of females with undetected CIN3 that progress to undetected cancer
$d_{d1}$	Proportion of females with detected CIN1 that recover
$1-d_{d1}$	Proportion of females with detected CIN1 that progress to detected CIN2
$h_{d1}$	Proportion of females with detected CIN2 that recover
$h_{d2}$	Proportion of females with detected CIN2 that regress to detected CIN1
$1-h_{d1}-h_{d2}$	Proportion of females with detected CIN2 that progress to detected CIN3
$q_{d1}$	Proportion of females with detected CIN3 that recover
$q_{d2}$	Proportion of females with detected CIN3 that regress to detected CIN2
$1-q_{d1}-q_{d2}$	Proportion of females with detected CIN3 that progress to detected cancer
$d_{a}$	Proportion of males with INM1 that recover
$1-d_m$	Proportion of males with INM1 that progress to INM2
$h_{m1}$	Proportion of males with INM2 that recover
$h_{m2}$	Proportion of males with INM2 that regress to INM1
$1-h_{m1}-h_{m2}$	Proportion of males with INM2 that progress to INM3
$q_{m1}$	Proportion of males with INM3 that recover
$q_{m2}$	Proportion of males with INM3 that regress to INM2
$1-q_{m1}-q_{m2}$	Proportion of males with INM3 that progress to HPV-related cancer

### Basic qualitative properties

The basic qualitative properties of the model (2.5) will now be explored to assess its well-posedness. Specifically, the non-negativity, boundedness, and invariance of its solutions will be explored. We claim the following result (the proof of Lemma 2.1 is given in Appendix A).

#### Lemma 2.1

Consider the model (2.5) with non-negative initial conditions and strict positivity condition for the initial values of $Q_{2u}, ~Q_{2d}$, and $J_{2m}$ (i.e., $Q_{2u}(0)>0, ~Q_{2d}(0) > 0$, and $J_{2m}(0) > 0$). Then, all solutions of the model remain non-negative for all time $t>0$.

Consider, next, the following biologically feasible region for the model: (2.5):

$$\begin{aligned} \mathcal {D}_{S} = \mathcal {D}_{S,f} \times \mathcal {D}_{S,m} \subset \mathbb {R}_+^{15} \times \mathbb {R}_+^{11}, \end{aligned}$$

where, $$\mathcal {D}_{S,f} = \{(S_f, V_f, E_f, I_f, P_f, Q_{1u},Q_{2u},Q_{3u},Q_{1d},Q_{2d},Q_{3d}, C_u, C_d, R_f^C, R_f) \in \mathbb {R}_+^{15} : 0 \le N_f \le \frac{\pi _f}{\mu }\}$$ and $$ \mathcal {D}_{S,m} = \{(S_m, V_m, E_m, I_m, P_m, J_{1m},J_{2m},J_{3m}, C_m, R^C_m, R_m) \in \mathbb {R}_+^{11} : 0 \le N_m \le \frac{\pi _m}{\mu }\}.$$

We claim the following result.

#### Lemma 2.2

The region $\mathcal {D}_{S}$ is positively invariant with respect to the flow generated by the model (2.5), and attracts all solutions of the model. Furthermore, all solutions of the model are bounded for all time $t \ge 0$.

#### Proof

Adding the equations for the rates of change of each of the epidemiological compartment of the model (2.5) shows that the equations for the rate of change of the total female and male populations are given, respectively, by

2.6 $$\begin{aligned} \dot{N}_f (t)=\pi _f - \mu N_f - \delta C_u -\delta _d C_d \le \pi _f - \mu N_f \,\,\textrm{and}\,\, \dot{N}_m (t)=\pi _m - \mu N_m, \end{aligned}$$

from which it, respectively, follows, using a standard comparison theorem (Lakshmikantham et al. 2016), that

2.7 $$\begin{aligned} N_f (t)\le N_f(0)e^{-\mu t} + \frac{\pi _f}{\mu }[1-e^{-\mu t}] \,\,\textrm{and}\,\, N_m (t)=N_m(0)e^{-\mu t} + \frac{\pi _m}{\mu }[1-e^{-\mu t}]. \end{aligned}$$

Consequently, $N_f(t) \le \frac{\pi _f}{\mu }$ and $N_m(t) \le \frac{\pi _m}{\mu }$ for all $t\ge 0$ provided that $N_f(0) \le \frac{\pi _f}{\mu }$ and $N_m(0) \le \frac{\pi _m}{\mu }$. In addition, Lemma 2.1 shows that solutions with non-negative initial conditions stays non-negative for all $t \ge 0$. Thus, these results (for the upper bounds of $N_f(t)$ and $N_m(t)$ and the non-negativity of the solutions of the model) show that any solution of the model with initial condition inside $\mathcal {D}_S$ stays inside $\mathcal {D}_S$ for $t \ge 0$, and the region $\mathcal {D}_S$ is positively invariant with respect to the model (2.5). Moreover, it follows from (2.6) that $\dot{N}_f(t) < 0$ whenever $N_{f}(t) > \frac{\pi _{f}}{\mu }$, and $\dot{N}_m(t) < 0$ whenever $N_m(t) > \frac{\pi _{a}}{\mu }$. Thus, any solution of the model (2.5) either enters $\mathcal {D}_S$ in a finite time, or $N_f(t)$ and $N_m(t)$ approach $\frac{\pi _f}{\mu }$ and $\frac{\pi _m}{\mu }$, respectively. Hence, all solutions of the model (2.5) in $\mathbb {R}_+^{15} \times \mathbb {R}_+^{11}$ are bounded for all time $t\ge 0$, and $\mathcal {D}_S$ is attracting (i.e., attracts all solutions of the model (2.5) in $\mathbb {R}_+^{15} \times \mathbb {R}_+^{11}$). $\square$

Since the region $\mathcal {D}_S$ is positively invariant and attracting, it can be concluded that the model (2.5) is well-posed mathematically and epidemiologically in the region $\mathcal {D}_S$. Hence, it is sufficient to study the dynamics of the model in the invariant and attracting region $\mathcal {D}_S$ (Hethcote 2000).

### Existence and local asymptotic stability of equilibria of the model (2.5)

#### Disease-free equilibrium (DFE)

The model (2.5) has a unique disease-free equilibrium (DFE) given by:

$$\begin{aligned} \mathcal {E}_{0s} =(S_f^*,V_f^*,0,0,0,0,0,0,0,0,0,0,0,0,0,S_m^*,V_m^*,0,0,0,0,0,0,0,0,0), \end{aligned}$$

where, $S_f^* = \frac{(1-q_f)\pi _f}{\xi _f + \mu }, \; V_{f}^* = \frac{\pi _f(\xi _f+\mu q_f)}{\mu (\xi _f+\mu )},\; S_m^* = \frac{(1-q_m)\pi _m}{\xi _m +\mu }, \; V_m^* = \frac{\pi _m(\xi _m+\mu q_m)}{\mu (\xi _m+\mu )}$ (note that $q_f$ and $q_m$ are fractions, hence $S_f^*>0$ and $S_m^*>0$). The stability of the DFE is explored using the *next generation operator* method (Diekmann et al. 1990; Van den Driessche and Watmough 2002). In particular, using the notation in (Van den Driessche and Watmough 2002), the associated matrices $F_s$ (for the new infection terms near the DFE) and $V_s$ (for the linear transition terms in the infected compartments) are given, respectively, by (note that **0**$_{n\times n}$ is the zero matrix of order *n*):

$$ F_s =\left[ \begin{array}{c|c} \textbf{0}_{11 \times 11} & \textbf{e}_2^T\mathbf {f_2} \\ \hline \textbf{e}_1^T\mathbf {f_1} & \textbf{0}_{7 \times 7} \end{array}\right] \; \; \; \text {and} \; \; \; V_s =\left[ \begin{array}{c|c} V_1 & \textbf{0}_{7 \times 7} \\ \hline \textbf{0}_{11 \times 11} & V_2 \end{array}\right] ,$$

where,

$$\begin{aligned} \textbf{e}_1&= (1,0,0,0,0,0,0), \\ ~\textbf{f}_1&= \frac{\beta _{fm} c_{mf} p_m^*}{N_m^*} ~(\eta _{f}, 1, \theta _{f}, \theta _{f}, \theta _f\kappa _{f}, \theta _f\kappa _f, \theta _f\nu , \theta _f\nu , \theta _f\nu \kappa _{fd}, 0, 0),\\ \textbf{e}_2&= (1,0,0,0,0,0,0,0,0,0,0), ~\textbf{f}_2 = \frac{\beta _{m f} c_{mf} p_f^*}{N^*_m}~(\eta _m, 1, \theta _m, \theta _m, \theta _m\kappa _m, \theta _m\kappa _m, 0),\\ V_1&= \left[ \begin{array}{c c c c c c c c c c c} k_1 & 0 & 0 & 0 & 0 & 0 & 0 & 0 & 0 & 0 & 0 \\ -\sigma _f & k_2 & 0 & 0 & 0 & 0 & 0 & 0 & 0 & 0 & 0 \\ 0 & -l_1 & k_3 & 0 & 0 & 0 & 0 & 0 & 0 & 0 & 0\\ 0 & 0 & -l_2 & k_4 & -h_{u2}y_f & 0 & 0 & 0 & 0 & 0 & 0 \\ 0 & 0 & 0 & -l_3 & k_5 & q_{u2}z_f & 0 & 0 & 0 & 0 & 0 \\ 0 & 0 & 0 & 0 & -l_4 & k_6 & 0 & 0 & 0 & 0 & 0 \\ 0 & 0 & 0 & -d_{u3}g_f & 0 & 0 & k_7 & -h_{d2}\zeta _2 & 0 & 0 & 0 \\ 0 & 0 & 0 & 0 & -h_{u3}y_f & 0 & -l_6 & k_8 & -q_{d2}\zeta _3 & 0 & 0 \\ 0 & 0 & 0 & 0 & 0 & -q_{u3}z_f & 0 & -l_7 & k_9 & 0 & 0 \\ 0 & 0 & 0 & 0 & 0 & -l_5 & 0 & 0 & 0 & k_{10} & 0 \\ 0 & 0 & 0 & 0 & 0 & 0 & 0 & 0 & -l_8 & -w_f & k_{11} \end{array}\right] , \end{aligned}$$

and,

$$\begin{aligned} V_2&= \left[ \begin{array}{c c c c c c c} e_1 & 0 & 0 & 0 & 0 & 0 & 0\\ -\sigma _m & e_2 & 0 & 0 & 0 & 0 & 0 \\ 0 & -(1-b_m)\psi _m & e_3 & 0 & 0 & 0 & 0\\ 0 & 0 & -(1-k_m)\alpha _m & e_4 & 0 & 0 & 0 \\ 0 & 0 & 0 & -(1-d_m)g_m & e_5 & 0 & 0\\ 0 & 0 & 0 & 0 & -f_1 & e_6 & 0 \\ 0 & 0 & 0 & 0 & 0 & -f_2 & e_7 \\ \end{array}\right] , \end{aligned}$$

with, $p_f^* = S_f^* + (1-\varepsilon _v) V_f^* = \frac{\pi _f}{\mu } \bigg ( 1-\varepsilon _v \; \frac{\xi _f + \mu q_f}{\xi _f + \mu }\bigg ), \; p_m^* = S_m^* + (1-\varepsilon _v) V_m^* = \frac{\pi _m}{\mu } \bigg ( 1-\varepsilon _v \; \frac{\xi _m + \mu q_m}{\xi _m + \mu }\bigg ), \,k_1 = \sigma _f+\mu , k_2 = \psi _f + \mu , k_3 = \alpha _f + \mu , k_4 = g_f+\mu , k_5 = y_f+\mu , k_6 = z_f+\mu , k_7 = \zeta _1+\mu , k_8 = \zeta _2+\mu , k_9 = \zeta _3+\mu , k_{10} = w_f+r_u+\delta +\mu , k_{11} = r_d+\delta _d+\mu , l_1 = (1-b_f)\psi _f, l_2 = (1-k_f)\alpha _f, l_3 = (1-d_{u1}-d_{u3})g_f, l_4 = (1-h_{u1}-h_{u2}-h_{u3})y_f, l_5 = (1-q_{u1}-q_{u2}-q_{u3})z_f, l_6 = (1-d_{d1})\zeta _1, l_7 = (1-h_{d1}-h_{d2})\zeta _2, l_8 = (1-q_{d1}-q_{d2})\zeta _3, e_1 = \sigma _m + \mu , e_2 = \psi _m + \mu , e_3 = \alpha _m + \mu , e_4 = g_m+\mu , e_5 = y_m+\mu , e_6 = z_m+\mu , e_7 = r_m+\mu , f_1 = (1-h_{m1}-h_{m2})y_m$, and $f_2 = (1-q_{m1}-q_{m2})z_m$. It is convenient to define the following quantity:

2.8 $$\begin{aligned} \mathcal {R}_{vs}= \rho (F_sV_s^{-1}) = \sqrt{\mathcal {R}_f \mathcal {R}_m}, \end{aligned}$$

where,

$$\begin{aligned} \mathcal {R}_f =&~ \frac{\beta _{fm}c_{mf}\mu p_m^*}{\pi _m} \left[ \frac{\eta _{f}}{k_1} + \frac{\sigma _f}{k_1k_2} + \frac{\theta _{f}\sigma _fl_1}{k_1k_2k_3} + \right. \\&+\left. \frac{\theta _{f}\sigma _fl_1l_2(\iota _{f2} +\kappa _fl_3k_6 +\kappa _fl_3l_4)}{k_1k_2k_3\iota _{f1}} + \frac{\theta _{f}\nu \sigma _fl_1l_2(j_{f1} +\kappa _{fd}j_{f2} +\kappa _{fd}j_{f3})}{k_1k_2k_3\iota _{f1}\iota _{f3}} \right] ,\\ \mathcal {R}_m =&~ \frac{\beta _{mf}c_{mf}\mu p_{f}^*}{\pi _m} \left[ \frac{\eta _m}{e_1} + \frac{\sigma _m}{e_1e_2} + \frac{\theta _m\sigma _m\psi _m(1-b_m)}{e_1e_2e_3} \right. \\&\left. + \frac{\theta _m\sigma _m \psi _m(1-b_m) \alpha _m (1-k_m)\{\iota _{m2} + \kappa _mg_m(1-d_m)e_6 + \kappa _mg_m(1-d_m)f_1\}}{e_1e_2e_3\iota _{m1}} \right] , \end{aligned}$$

with,

$$\begin{aligned} \iota _{f1}&= k_4k_5k_6-l_3h_{u2}y_fk_6-k_4l_4q_{u2}z_f, \; \iota _{f2} = k_5k_6-l_4q_{u2}z_f,\\ \iota _{f3}&= k_7k_8k_9-l_6h_{d2}\zeta _2k_9-k_7l_7q_{d2}\zeta _3, \\ j_{f1}&= d_{u3}g_f(k_5k_6-l_4q_{u2}z_f)(k_8k_9-l_7q_{d2}\zeta _3) + l_3h_{u3}y_fh_{d2}\zeta _2k_6k_9 \\&\quad + l_3l_4q_{u3}z_fq_{d2}\zeta _3h_{d2}\zeta _2, \\ j_{f2}&= d_{u3}g_fl_6k_9(k_5k_6-l_4q_{u2}z_f) + l_3h_{u3}y_fk_6k_7k_9 + l_3l_4q_{u3}z_fq_{d2}\zeta _3k_7, \\ j_{f3}&= d_{u3}g_fl_6l_7(k_5k_6-l_4q_{u2}z_f) + l_3h_{u3}y_fl_7k_6k_7 + l_3l_4q_{u3}z_f(k_7k_8-l_6h_{d2}\zeta _2), \\ \iota _{m1}&= e_4e_5e_6-(1-d_m)g_mh_{m2}y_me_6-e_4f_1q_{m2}z_m, \; \iota _{m2} = e_5e_6-f_1q_{m2}z_m. \end{aligned}$$

It can be shown that the expression for $\mathcal {R}_{vs}$, given in (2.8), is non-negative (see Appendix B for the proof). The result below follows from Theorem 2 of (Van den Driessche and Watmough 2002).

##### Theorem 2.3

The *DFE*, $\mathcal {E}_{0s}$, of the model (2.5) is locally-asymptotically stable if $\mathcal {R}_{vs}< 1$, and unstable if $\mathcal {R}_{vs}>1.$

The epidemiological implication of Theorem 2.3 is that a small influx of HPV-infected individuals into the community (i.e., initial number of infected individuals lie within the basin of attraction of the DFE) will not generate a large outbreak of HPV in the community if the interventions implemented in the community (vaccination and Pap screening in this case) can bring, and maintain, $\mathcal {R}_{vs}$ to a value less than unity (and the disease ultimately dies out). The threshold quantity $\mathcal {R}_{vs}$ is the *control reproduction number* of the model (2.5). It measures the average number of new HPV cases generated by a typical HPV-infected individual (female or male) if introduced into a community where a certain proportion is vaccinated and/or receiving Pap screening. It is convenient to let

2.9 $$\begin{aligned} v_f^* := \frac{V_f^*}{N_f^*} = \frac{\xi _f+\mu q_f}{\xi _f+\mu }, ~ v_m^* := \frac{V_m^*}{N_m^*}= \frac{\xi _m+\mu q_m}{\xi _m+\mu } \end{aligned}$$

be the fraction of females and males vaccinated at steady-state, respectively. It follows, by using (2.9) in (2.8), that the control reproduction number $\mathcal {R}_{vs}$ can be re-written in terms of these steady-state vaccination fractions as:

2.10 $$\begin{aligned} \mathcal {R}_{vs}(v_f^*, v_m^*) = {\mathcal {R}_{cs}}\sqrt{(1-\varepsilon _vv_m^*)(1-\varepsilon _vv_f^*)}, \end{aligned}$$

where,

2.11 $$\begin{aligned} \mathcal {R}_{cs}= \mathcal {R}_{vs}|_{q_f=\xi _f=q_m=\xi _m=0} \end{aligned}$$

is the control reproduction number of the model in the absence of vaccination (i.e., the control reproduction number without vaccination but with screening). It is convenient to define $\mathcal {R}_0= \mathcal {R}_{vs}|_{q_f = \xi _f = q_m = \xi _m = d_{u3} = h_{u3} = q_{u3} = \nu = 0} = \sqrt{\mathcal {R}_{0f}\mathcal {R}_{0m}},$ where,

$$\begin{aligned} \mathcal {R}_{0f}=&~ \beta _{fm}c_{mf}\left[ \frac{\eta _{f}}{k_1} + \frac{\sigma _f}{k_1k_2} + \frac{\theta _{f}\sigma _fl_1}{k_1k_2k_3} +\frac{\theta _{f}\sigma _fl_1l_2(\tilde{\iota }_{f2} +\kappa _f\tilde{l_3}k_6 +\kappa _f\tilde{l_3}\tilde{l_4})}{k_1k_2k_3\tilde{\iota }_{f1}}\right] , \\ \mathcal {R}_{0m}=&~ \frac{\beta _{mf}c_{mf}\pi _f}{\pi _m} \left[ \frac{\eta _m}{e_1} + \frac{\sigma _m}{e_1e_2} + \frac{\theta _m\sigma _m\psi _m(1-b_m)}{e_1e_2e_3} \right. \\&\left. + \frac{\theta _m\sigma _m \psi _m(1-b_m) \alpha _m (1-k_m)\{\iota _{a2} + \kappa _mg_m(1-d_m)e_6 + \kappa _mg_m(1-d_m)f_1\}}{e_1e_2e_3\iota _{a1}} \right] , \end{aligned}$$

with, $\tilde{\iota }_{f1} = \iota _{f1}|_{d_{u3}=h_{u3}=0} = k_4k_5k_6-\tilde{l_3}h_{u2}y_fk_6-k_4\tilde{l_4}q_{u2}z_f, \;\tilde{\iota }_{f2} = \iota _{f2}|_{h_{u3}=0} = k_5k_6-\tilde{l_4}q_{u2}z_f,\; \tilde{l_3} = l_3|_{d_{u3}=0} = (1-d_{u1})g_f, \;\tilde{l_4} = l_4|_{h_{u3}=0} = (1-h_{u1}-h_{u2})y_f$. The quantity $\mathcal {R}_0$ is the *basic reproduction number* of the model (which represents the average number of new HPV cases generated by a typical HPV-infected individual introduced into a completely susceptible population). Then, the quantity $\mathcal {R}_{cs}$, given in (2.11), can be expressed as $\mathcal {R}_{cs}= \sqrt{\mathcal {R}_{csf}\mathcal {R}_{0m}},$ where

$$\begin{aligned} \mathcal {R}_{csf}=~&\mathcal {R}_{0f}- \left( 1-\frac{\tilde{\iota }_{f1}}{\iota _{f1}}\right) \beta _{fm}c_{mf} \left[ \frac{\theta _{f}\sigma _fl_1l_2(\tilde{\iota }_{f2} +\kappa _f\tilde{l_3}k_6 +\kappa _f\tilde{l_3}\tilde{l_4})}{k_1k_2k_3\tilde{\iota }_{f1}}\right] \\&- \frac{\theta _f\sigma _f l_1l_2\kappa _f(\tilde{l_3}h_{u3}y_f +d_{u3}g_f\tilde{l_4} -d_{u3}g_fh_{u3}y_f)}{k_1k_2k_3\iota _{f1}} \\&\quad + \beta _{fm}c_{mf} \left[ \frac{\theta _{f}\nu \sigma _fl_1l_2(j_{f1} +\kappa _{fd}j_{f2} +\kappa _{fd}j_{f3})}{k_1k_2k_3\iota _{f1}\iota _{f3}} \right] . \nonumber \end{aligned}$$

**Fig. 2:**
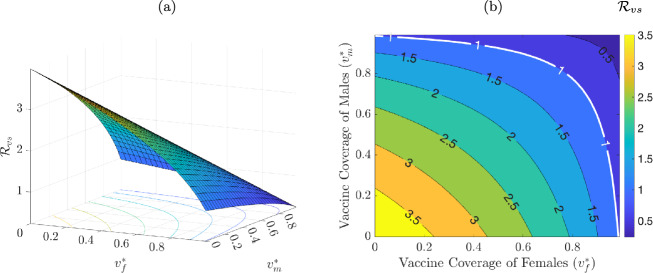
(a) Three-dimensional and (b) contour plot of the control reproduction number, $\mathcal {R}_{vs}$, of the model (2.5), as a function of vaccine coverage of females at steady-state ($v_f^*$) and vaccine coverage of males at steady-state ($v_m^*$), for vaccine efficacy $\varepsilon _v = 0.95$. The white curve represents the 1-level set $\mathcal {R}_{vs}(v_f^*,v_m^*) = 1$. The other parameter values used to generate the plots are as given in Table 9.

Furthermore, it can be seen, using (2.10), that the gradient of $\mathcal {R}_{vs}$ is:

$$\begin{aligned}\left( \frac{\partial \mathcal {R}_{vs}}{\partial v_f^*}, \frac{\partial \mathcal {R}_{vs}}{\partial v_m^*}\right) = \frac{-\varepsilon _v{\mathcal {R}_{cs}}}{2\sqrt{(1-\varepsilon _v v_m^*)(1-\varepsilon _v v_f^*)}} \left( 1-\varepsilon _v v_m^*, 1-\varepsilon _v v_{f}^*\right) ,\end{aligned}$$

from which it follows that both $\frac{\partial \mathcal {R}_{vs}}{\partial v_f^*}$ and $\frac{\partial \mathcal {R}_{vs}}{\partial v_m^*}$ are strictly negative for $0< \varepsilon _v < 1$ and $0 \le v_f^*, v_m^* \le 1$. Thus, $\mathcal {R}_{vs}$ is a decreasing function of $v_f^*$ and $v_m^*$ for any fixed values of the other parameters (this is depicted in a 3-dimensional plot in Figure 2 (a), for $\mathcal {R}_{vs}$ as a function of the two vaccination coverages). Since a reduction in the control reproduction number implies a reduction in the disease burden (as measured in terms of the number of HPV infections, HPV-related cancer cases and mortality etc.), the above analysis shows that the HPV vaccination program implemented in Korea will have a positive impact in reducing HPV burden in the country. In other words, a vaccination program (using an HPV vaccine with protective efficacy $0< \varepsilon _v < 1$) combined with Pap screening will result in a decrease in HPV burden (and related cancers) regardless of the values of the proportions of females and males vaccinated at steady-state. This result is also illustrated in a contour plot of the control reproduction number, $\mathcal {R}_{vs}$, as a function of $v_f^*$ and $v_m^*$, depicted in Figure 2 (b). This figure shows that $\mathcal {R}_{vs}$ decreases (i.e., disease burden decreases), with increasing values of the proportion of females and males vaccinated at steady-state. Furthermore, this figure shows that, using the model (2.5) with the baseline values in Table 9, the use of an HPV vaccine (with protective efficacy of 95%) and baseline Pap screening coverage can lead to the effective control of HPV in Korea for a certain combination of the vaccination coverage for females and males (e.g., 88% of females vaccinated combined with 65% of males vaccinated at steady-state; since this combination of efficacy and coverages will bring $\mathcal {R}_{vs}$ to a value less than one, which is needed for the local asymptotic stability of the disease-free equilibrium, in line with Theorem 2.3). It can also be seen from this contour plot that $\mathcal {R}_{vs}$ can be brought to a value less than one if nearly 100% of females or males are vaccinated (but this is generally not realistically attainable).

Using the baseline values of the parameters of the model (2.5), given in Table 9, it can be seen that the the control ($\mathcal {R}_{vs}$) and basic ($\mathcal {R}_0$) reproduction numbers for South Korea before the implementation of the National Immunization Program for HPV in 2016 are $\mathcal {R}_{vs}= 3.0764$ and $\mathcal {R}_0= 3.9858$ (this value of $\mathcal {R}_0$ is within the range of $\mathcal {R}_0$ for HPV reported in (Baussano et al. 2018)), respectively. In other words, based on the HPV vaccination coverage for females in Korea before the implementation of the National Immunization Program (estimated to be about 42% (Choi et al. 2022)) and the baseline Pap screening coverage (estimated to be about 61%), HPV (and associated cancers) will continue to persist in Korea (since $\mathcal {R}_0$ is about 4, and the pre-NIP vaccination and Pap screening only reduce $\mathcal {R}_0$ by about 23%). However, for post-NIP vaccination (where the vaccination coverage for females in Korea is estimated to be 88% (Choi et al. 2022; Kim et al. 2018)), the baseline value of $\mathcal {R}_{vs}$ is 1.6181, which represents a 59% reduction of the basic reproduction number, $\mathcal {R}_0$. Here, too, HPV will persist (since $\mathcal {R}_{vs}>1$), *albeit* HPV burden will be significantly reduced, in comparison to the pre-NIP era.

**Computation of vaccine-derived herd immunity threshold:** A precise expression for the vaccine-derived *herd immunity threshold* (i.e., the minimum proportion of susceptible individuals in the community that needs to be vaccinated in order to protect the ones that cannot be vaccinated) can be derived by setting the control reproduction number ($\mathcal {R}_{vs}$) to one and solving for the proportions of females and males vaccinated at steady-state ($v_f^*$ and $v_m^*$) (Elbasha and Gumel 2021; Gumel et al. 2006; Malik et al. 2013; McLean and Blower 1993; Pant and Gumel 2024). Let $\mathcal {R}_{cs}>1$. Then, setting the control reproduction number $\mathcal {R}_{vs}$, given by Equation (2.10), to one (and simplifying) shows that (noting that $0<\varepsilon _v<1$, $0 \le v_f^*, v_m^* \le 1$, and $\mathcal {R}_{cs}>1$):

2.12 $$\begin{aligned} (1-\varepsilon _v v_m^*)(1-\varepsilon _v v_f^*) = \displaystyle \frac{1}{(\mathcal {R}_{cs})^2}, \,\,\,\, \,\,\,\,0 \le v_f^*, v_m^* \le 1, \,\,\,\, \text{ and } \,\,\,\, \mathcal {R}_{cs}>1. \end{aligned}$$

**Fig. 3:**
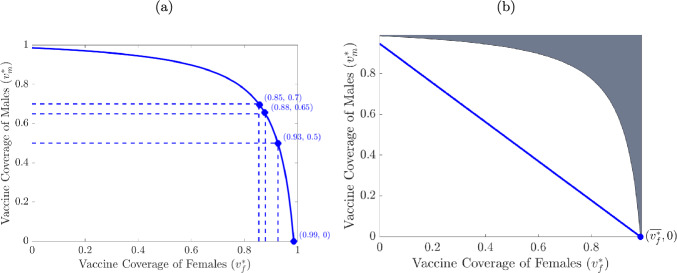
(a) The level set $\mathcal {R}_{vs}(v_f^*,v_m^*) = 1$ (shown in solid blue curve) for the model (2.5), as a function of steady-state vaccination coverage for females ($v_f^*$) and males ($v_m^*$), showing some of the, among the infinitely many, $(v_f^*,v_m^*)$ pairs that fall on the level set. (b) Geometric interpretation of the constrained optimization problem (2.14) associated with the model. The optimal solution in the feasible (shaded gray) set that minimizes $n_f^*v_f^* +n_m^*v_m^*$ is the point $(\overline{v_f^*},0)$ (i.e., the point in the shaded region that intersects the blue line, which is the line closest to the origin among the infinitely many lines with slope $-n_f^*/n_m^*$, which also intersect the feasible set). The parameter values used to generate this figure are as given in Table 9.

It follows from the level curve $\mathcal {R}_{vs}(v_f^*,v_m^*)=1$, represented by the white curve in the contour plot depicted in Figure 2 (b), that increasing the steady-state vaccination coverage of one gender group (females or males) will reduce the coverage level of the other group needed to bring (and maintain) $\mathcal {R}_{vs}$ to a value below one. Some representative $(v_f^*,v_m^*)$ pairs are illustrated in Figure 3 (a), showing that if no susceptible males are vaccinated at steady-state, then at least 99% of the sexually active females need to be vaccinated to bring the reproduction threshold $\mathcal {R}_{vs}$ below one (so that the disease can be effectively controlled and/or eliminated). However, this figure further shows that, if half of the males in the community are vaccinated, then 93% of the females need to be vaccinated in order to bring the reproduction threshold below one. Furthermore, Figure 3 (a) shows that if the vaccination coverage of males is increased (from 50%) to 70%, then the vaccination coverage needed for the female population decreases to 85%. Finally, if the post-NIP vaccination coverage of females in Korea is maintained at the $88\%$ level (i.e., $v_f^*=0.88$), then it follows from Figure 3 (a) that vaccinating $65\%$ of males would be sufficient to bring $\mathcal {R}_{vs}$ below one in Korea. In other words, this study shows that HPV can be effectively controlled and/or eliminated in Korea if the current HPV vaccination coverage in females is complemented with HPV vaccination of males with at least 65% coverage at steady-state. Solving for $v_m^*$ from (2.12) gives:

2.13 $$\begin{aligned} {v_m^*} = \displaystyle \frac{1}{\varepsilon _v}\left[ 1-\frac{1}{{(\mathcal {R}_{cs})^2}(1-\varepsilon _v v_{f}^*)}\right] =:(v_m^*)_c,\,\,\,\,\,\, { \textrm{with}\,\,0<\varepsilon _v<1,\,\,\textrm{and}}\,\,\,0 \le v_f^* \le 1. \end{aligned}$$

It follows from (2.10) and (2.13) that the control reproduction number, $\mathcal {R}_{vs}$, is less than one if and only if $v_m^* > (v_m^*)_c=f(v_{f}^*;\varepsilon _v,\mathcal {R}_0)$, and it equals one if $v_m^* = (v_m^*)_c$. Hence, the result of Theorem 2.3 can be re-written in terms of the vaccine-derived herd immunity threshold ($(v_f^*)_c$) as follows:

##### Theorem 2.4

The *DFE*, $\mathcal {E}_{0s}$, of the model (2.5) is locally-asymptotically stable if $v_m^* > (v_m^*)_c$ (i.e., $\mathcal {R}_{vs}< 1$), and unstable if $v_m^* < (v_m^*)_c$ (i.e., $\mathcal {R}_{vs}>1$).

Theorem 2.4 implies that HPV will be eliminated in Korea if the proportion of males vaccinated at steady-state exceeds the herd immunity threshold value $(v_m^*)_c$. In other words, Theorem 2.4 shows that, using the post-NIP vaccination coverage for females (i.e., $v_f^* = 0.88$) and the other parameters of the model (2.5) maintained at their baseline values given in Table 9, vaccine-derived herd immunity can be achieved in Korea by vaccinating 65% of males (i.e., $(v_m^*)_c=0.65$ for Korea). However, if males are not vaccinated (i.e., $v_m^* = 0$), then, $v_f^* = 0.99$ (i.e., in this case, at least 99% of females must be vaccinated to achieve herd immunity, and, consequently, eliminate the disease).

**Optimization of total vaccination coverage at steady-state:** The objective here is to determine the optimal values of the steady-state vaccination coverage pair, $(v_f^*, v_m^*)$, needed to minimize the total vaccination coverage (for both females and males in Korea) that results in $\mathcal {R}_{vs}<1$ in the country. In other words, the aim here is to minimize the following objective functional, for the total vaccination coverage in Korea:

$$\begin{aligned} \frac{V_f^* +V_m^*}{N_f^* +N_m^*} = \left( \frac{N_f^*}{N_f^*+N_m^*}\right) \frac{V_f^*}{N_f^*} +\left( \frac{N_m^*}{N_f^* +N_m^*}\right) \frac{V_m^*}{N_m^*} =: n_f^*v_f^* +n_m^*v_m^*, \end{aligned}$$

subject to reducing the value of $\mathcal {R}_{vs}$ below one. More formally, the aim is to

2.14 $$\begin{aligned} \text{ minimize } \,\,\,\,\, n_f^*v_f^* +n_m^*v_m^* \,\,\,\,\, \text{ subject } \text{ to } \,\,\,\,\, 0 \le v_f^*, v_m^* \le 1,\,\,\textrm{and}\,\, \mathcal {R}_{vs}\le 1, \end{aligned}$$

where $\mathcal {R}_{vs}$ is given by (2.10) with fixed vaccine efficacy $\varepsilon _v = 0.95$. The solution to this nonlinear optimization problem is obtained by using the geometric approach introduced by Elbasha and Gumel (Elbasha and Gumel 2021). The approach entails defining the feasible set (that satisfies the constraints of the optimization problem) and finding the points on the lines $n_f^*v_f^* +n_m^*v_m^*=k$ (with $k\in {\mathbb R}$, and with slope of the line, $dv_m^*/dv_f^*$, equal to $-{n_f^*}/{n_m^*}$) which intersect the feasible set. The optimal points will then lie on the line with the lowest $y-$intercept on the $(v_f^*,v_m^*)$-plane. The feasible set for the optimization problem (2.14) is $\{(v_f^*,v_m^*)\in [0, 1]^2 ~\vert ~ v_m^* \ge (v_m^*)_c = f(v_f^*)\},$ and is depicted by the shaded region in Figure 3 (b)). Since the line $n_f^*v_f^* +n_m^*v_m^* = k$ has slope $-{n_f^*}/{n_{m^*}} = -{\pi _f}/{\pi _m} >-1$, it follows (from the fact that the feasible set is symmetric with respect to the line $v_m^* = v_f^*$ (i.e., the inequality $v_m^* \ge f(v_f^*)$ remains the same if the two variables are interchanged) and Figure 3 (b)) that *k* is minimized when the line intersects the feasible set uniquely on the x-axis. The intersection occurs at the unique point on the $v_f^*$-intercept of the line $v_m^* = f(v_f^*)$, given by (where $\overline{v_f^*}$ denotes the optimal value for $v_f^*$):

2.15 $$\begin{aligned} (v_f^*, v_m^*) = (\overline{v_f^*}, 0) = \left( \frac{1}{\varepsilon _v}\left( 1-\frac{1}{{\mathcal {R}_{cs}^2}}\right) , 0\right) . \end{aligned}$$

Since the value of $\overline{v_f^*}$, computed using the baseline values of the parameters in the expression for $\mathcal {R}_0$ given in Table 9 with $\varepsilon _v=0.95$, is 0.99, it follows from (2.15) that the total vaccination coverage is optimized if 99% of females and 0% of males are vaccinated at steady-state. Thus, this study shows that the current (post-NIP) HPV vaccination coverage (of 88% vaccination of females) is insufficient to lead to vaccine-derived herd immunity. More (11%) unvaccinated females need to be vaccinated to achieve such immunity (with minimum number of vaccinated individuals). If 88% (i.e., the current vaccination coverage of females) is the maximum achievable vaccine uptake in Korea in each group (females and males) due to factors including vaccine hesitancy, limited availability and access to public health resources, and individual medical conditions, then the optimal steady-state vaccination coverage is 88% of females and 65% of males.

#### Existence and local asymptotic stability of endemic equilibria: special case

In this section, conditions for the existence of endemic equilibria (EE) of the model (2.5) will be explored for a special case with negligible mortality for females due to cervical cancer (i.e., the model with $\delta =\delta _d=0$ will now be considered). Although this assumption (for the negligible disease-induced mortality) is made for mathematical tractability, it can also be epidemiologically justified for the dynamics of HPV and its related cancers in South Korea, where the average number of deaths caused by cervical cancer each year ranges from 1, 000 to 1, 500 (which represents about $0.006\%$ to $0.009\%$ of the sexually active females in South Korea). Setting $\delta =\delta _d=0$ in the model (2.5) shows that $N_f(t)\rightarrow \pi _f/\mu$ and $N_m(t)\rightarrow \pi _m/\mu$ as $t\rightarrow \infty$. For the purpose of the analysis in this subsection, the total populations $N_f(t)$ and $N_m(t)$ will now be replaced by their limiting values $N_f^*=\pi _f/\mu$ and $\pi _m/\mu$, respectively. It should be mentioned that, since females with cervical cancer (i.e., individuals in the $C_u$ and $C_d$ compartments) do not transmit HPV, the control reproduction number $\mathcal {R}_{vs}$ of the special case of the model (2.5) is the same as that for the full model (2.5). Let

$$\begin{aligned} \mathcal {E}_{1s} = (&S_f^{**},V_f^{**},E_f^{**}, I_f^{**},P_f^{**},Q_{1u}^{**},Q_{2u}^{**},Q_{3u}^{**},Q_{1d}^{**},Q_{2d}^{**},Q_{3d}^{**},C_u^{**},C_d^{**},R_f^{C**},R_f^{**},\\ &S_m^{**},V_m^{**},E_m^{**},I_m^{**},P_m^{**},J_{1m}^{**},J_{2m}^{**},J_{3m}^{**},C_m^{**},R_m^{C**},R_m^{**}) \end{aligned}$$

represent any arbitrary endemic equilibrium (EE) of the aforementioned special case of the heterosexual model (2.5). In addition, substituting $N_f(t)=\pi _f/\mu$ and $N_m(t)=\pi _m/\mu$ into the expressions for the forces of infections, given in (2.4), shows that:

2.16 $$\begin{aligned} \lambda _{mf}^{**} =&\left( \beta _{mf} c_{mf}\mu \right) \left[ \frac{I_m^{**} + \eta _m E_m^{**} + \theta _m [P_m^{**} +J_{1m}^{**}+ \kappa _m( J_{2m}^{**} +J_{3m}^{**})]}{\pi _m}\right] , \\ \lambda _{fm}^{**} =&\left( \beta _{fm} c_{mf}\mu \right) \left[ \frac{I_f^{**} + \eta _{f}E_f^{**} + \theta _{f} [P_f^{**} +Q_{1u}^{**} + \kappa _f (Q_{2u}^{**} +Q_{3u}^{**})+\nu \{Q_{1d}^{**} +\kappa _{fd}(Q_{2d}^{**} +Q_{3d}^{**})\}]}{\pi _m}\right] .\nonumber \end{aligned}$$

It follows then that the special case of the model (2.5) at the endemic equilibrium satisfies the following steady-state relations:

2.17 $$\begin{aligned} S_{f}^{**}&= \frac{(1-q_{f})\pi _{f}}{\lambda _{mf}^{**}+\xi _f +\mu }, \;\; V_{f}^{**} = \frac{\pi _f}{(1-\varepsilon _v)\lambda _{mf}^{**} + \mu }\left[ q_f + \frac{(1-q_f)\xi _f}{\lambda _{mf}^{**} +\xi _f +\mu } \right] , \nonumber \\ E_{f}^{**}&= \frac{\lambda _{mf}^{**}}{k_1} \cdot \frac{\pi _f(1-\varepsilon _v)\lambda _{mf}^{**} +\pi _f\mu (1-q_f\varepsilon _v) +\pi _f(1-\varepsilon _v)\xi _f}{(\lambda _{mf}^{**} +\xi _f +\mu )[(1-\varepsilon _v)\lambda _{mf}^{**} +\mu ]},\nonumber \\ I_{f}^{**}&= \frac{\sigma _{f} E_{f}^{**}}{k_2}, \;\; P_{f}^{**} = \frac{l_1 I^{**}_{f}}{k_3}, \;\; Q_{1u}^{**} = \frac{l_2P_f^{**} +h_{u2}y_fQ_{2u}^{**}}{k_4}, \;\; Q_{2u}^{**} = \frac{l_3Q_{1u}^{**} +q_{u2}z_fQ_{3u}^{**}}{k_5},\nonumber \\ Q_{3u}^{**}&= \frac{l_4Q_{2u}^{**}}{k_6}, \;\; Q_{1d}^{**} = \frac{d_{u3}g_fQ_{1u}^{**} +h_{d2}\zeta _2Q_{2d}^{**}}{k_7}, \;\; Q_{2d}^{**} = \frac{h_{u3}y_fQ_{2u}^{**} +l_6Q_{1d}^{**} +q_{d2}\zeta _3Q_{3d}^{**}}{k_8},\nonumber \\ Q_{3d}^{**}&= \frac{q_{u3}z_fQ_{3u}^{**} +l_7Q_{2d}^{**}}{k_9}, \; C_u^{**} = \frac{l_5Q_{3u}^{**}}{k_{10}}, \; C_d^{**} = \frac{l_8Q_{3d}^{**} +w_fC_u^{**}}{k_{11}}, \; R_f^{C**} = \frac{r_uC_u^{**} +r_dC_d^{**}}{\mu },\nonumber \\ R_f^{**}&= \frac{1}{\mu }(b_f\psi _fI_f^{**} +k_f\alpha _fP_f^{**} +d_{u1}g_fQ_{1u}^{**} +h_{u1}y_fQ_{2u}^{**} +q_{u1}z_fQ_{3u}^{**} +d_{d1}\zeta _1Q_{1d}^{**} \\&~~~ +h_{d1}\zeta _2Q_{2d}^{**} +q_{d1}\zeta _3Q_{3d}^{**}),\nonumber \\ S_m^{**}&= \frac{(1-q_m)\pi _m}{\lambda _{fm}^{**}+\xi _m +\mu }, \;\; V_m^{**} = \frac{\pi _m}{(1-\varepsilon _v)\lambda _{fm}^{**} + \mu }\left[ q_m + \frac{(1-q_m)\xi _m}{\lambda _{fm}^{**} +\xi _m +\mu } \right] ,\nonumber \\ E_m^{**}&= \frac{\lambda _{fm}^{**}}{e_1} \cdot \frac{\pi _m(1-\varepsilon _v)\lambda _{fm}^{**} +\pi _m\mu (1-q_m\varepsilon _v) +\pi _m(1-\varepsilon _v)\xi _m }{(\lambda _{fm}^{**} +\xi _m +\mu )[(1-\varepsilon _v)\lambda _{fm}^{**} +\mu ]},\nonumber \\ I_m^{**}&= \frac{\sigma _m E_m^{**}}{e_2}, \;\; P_m^{**} = \frac{(1-b_m)\psi _m I^{**}_m}{e_3}, \nonumber \\ J_{1m}^{**}&= \frac{(1-k_m)\alpha _mP_m^{**} +h_{m2}y_mJ_{2m}^{**}}{e_4}, \;\; J_{2m}^{**} = \frac{(1-d_m)g_mJ_{1m}^{**} +q_{m2}z_mJ_{3m}^{**}}{e_5}, \;\; J_{3m}^{**} = \frac{f_1J_{2m}^{**}}{e_6}, \nonumber \\ C_m^{**}&= \frac{f_2J_{3m}^{**}}{e_7}, \;\; R_m^{C**} =\frac{r_mC_m^{**}}{\mu }, \nonumber \\ R_m^{**}&= \frac{1}{\mu }\left( b_m\psi _mI_m^{**} +k_m\alpha _mP_m^{**} +d_mg_mJ_{1m}^{**} +h_{m1}y_mJ_{2m}^{**} +q_{m1}z_mJ_{3m}^{**} \right) .\nonumber \end{aligned}$$

Substituting (2.17) into (2.16), and simplifying, gives

2.18 $$\begin{aligned} \lambda _{mf}^{**} = \frac{\lambda _{fm}^{**}\mathcal {R}_m\pi _m\mu (\xi _f+\mu )\{(1-\varepsilon _v)\lambda _{fm}^{**} +\mu (1-q_m\varepsilon _v) +(1-\varepsilon _v)\xi _m\}}{\pi _f[\mu (1-q_f\varepsilon _v) +(1-\varepsilon _v)\xi _f](\lambda _{fm}^{**} +\xi _m +\mu )[(1-\varepsilon _v)\lambda _{fm}^{**} +\mu ]}, \end{aligned}$$

and,

2.19 $$\begin{aligned} \lambda _{fm}^{**} = \frac{\lambda _{mf}^{**}\mathcal {R}_{f}\pi _f\mu (\xi _m+\mu )\{(1-\varepsilon _v)\lambda _{mf}^{**} +\mu (1-q_f\varepsilon _v) +(1-\varepsilon _v)\xi _f\}}{\pi _m[\mu (1-q_m\varepsilon _v) +(1-\varepsilon _v)\xi _m](\lambda _{mf}^{**} +\xi _f +\mu )[(1-\varepsilon _v)\lambda _{mf}^{**} +\mu ]}. \end{aligned}$$

Substituting (2.19) into (2.18) leads to the following quartic in $$\lambda _{mf}^{**}$$:

2.20 $$\begin{aligned} \sum _{i=0}^4 a_i\left( \lambda _{mf}^{**}\right) ^i = 0, \end{aligned}$$

where the coefficients $a_i$ (with $i=0,1,\cdots ,4$) are given in Appendix C. It follows from the expressions for $a_i$ (in Appendix C) that the coefficient $a_4$ is always non-negative (given that the parameters of the model (2.5) are all non-negative), and the coefficients $a_1, a_2$, and $a_3$ are all non-negative if $\mathcal {R}_{vs}\le 1$. Furthermore, $a_0>0$ if and only if $\mathcal {R}_{vs}<1,$ and $a_0=0$ if $\mathcal {R}_{vs}=1.$ Thus, it follows, using the Descartes’ Rule of Signs, that the special case of the model (2.5) can have one or three positive (endemic) equilibria whenever the control reproduction number $\mathcal {R}_{vs}$ exceeds unity, and no endemic equilibria otherwise. This result is summarized below.

##### Theorem 2.5

The special case of the model (2.5), with $\delta =\delta _d=0$, has one or three positive endemic equilibria whenever $\mathcal {R}_{vs}> 1,$ and no endemic equilibrium whenever $\mathcal {R}_{vs}\le 1.$

The various possibilities for the number of equilibria for the special case of the model are tabulated in Table 4, from which it can be deduced that the special case of the model can have one or three endemic equilibria when the associated control reproduction exceeds one. Table 4 also shows that the special case of the model does not have multiple endemic equilibria when the control reproduction number $\mathcal {R}_{vs}$ is less than one, thus ruling out the existence of backward bifurcation (a dynamic behavior characterized by the co-existence of multiple stable attractors (one disease-free and one endemic) when the reproduction number of the model is less than unity) in the special case of the model. The presence of backward bifurcation in the transmission dynamics of an infectious disease makes its effective control or elimination more difficult (Alsaleh and Gumel 2014; Brauer 2004; Dushoff et al. 1998; Gumel 2012; Malik et al. 2013; Sharomi et al. 2007). This result (for the non-existence of backward bifurcation in the model (2.5) when the disease-induced mortality is negligible) is consistent with the result in (Gumel 2012) (Theorem 6, see also (Sharomi et al. 2007)).

**Table 4:** Number of possible positive real roots of the polynomial (2.20) for values of the control reproduction number, $\mathcal {R}_{vs}$, greater, equal to, or less than one

Case		$a_4$	$a_3$	$a_2$	$a_1$	$a_0$	Number of sign changes	Number of possible positive real roots
$\mathcal {R}_{vs}>1$	1	$+$	$+$	$+$	$+$	−	1	1
	2	$+$	$+$	$+$	−	−	1	1
	3	$+$	$+$	−	$+$	−	3	1 or 3
	4	$+$	$+$	−	−	−	1	1
	5	$+$	−	$+$	$+$	−	3	1 or 3
	6	$+$	−	$+$	−	−	3	1 or 3
	7	$+$	−	−	$+$	−	3	1 or 3
	8	$+$	−	−	−	−	1	1
$\mathcal {R}_{vs}=1$	9	$+$	$+$	$+$	$+$	0	0	0
$\mathcal {R}_{vs}<1$	10	$+$	$+$	$+$	$+$	$+$	0	0

In order to explore the local asymptotic stability of an endemic equilibrium of the aforementioned special case of the model (2.5), an additional assumption of perfect vaccine efficacy ($\varepsilon _v=1$) is made. Although also made for mathematical tractability, this additional assumption can be justified considering the fact that the HPV vaccines currently being used in Korea (Cervarix^®^ and Gardasil^®^) are highly efficacious, with efficacy above 90% (Cheng et al. 2020).

It is convenient to define $\tilde{\mathcal {R}}_{vs}:= \mathcal {R}_{vs}|_{\varepsilon _v = 1}$. We claim the following result.

##### Theorem 2.6

The special case of the model (2.5) with no cancer-induced mortality in females (i.e., $\delta =\delta _d=0$) and with perfect vaccine protective efficacy (i.e., $\varepsilon _v=1$) has a unique endemic equilibrium that is locally-asymptotically stable whenever $\tilde{\mathcal {R}}_{vs}>1$.

**Fig. 4:**
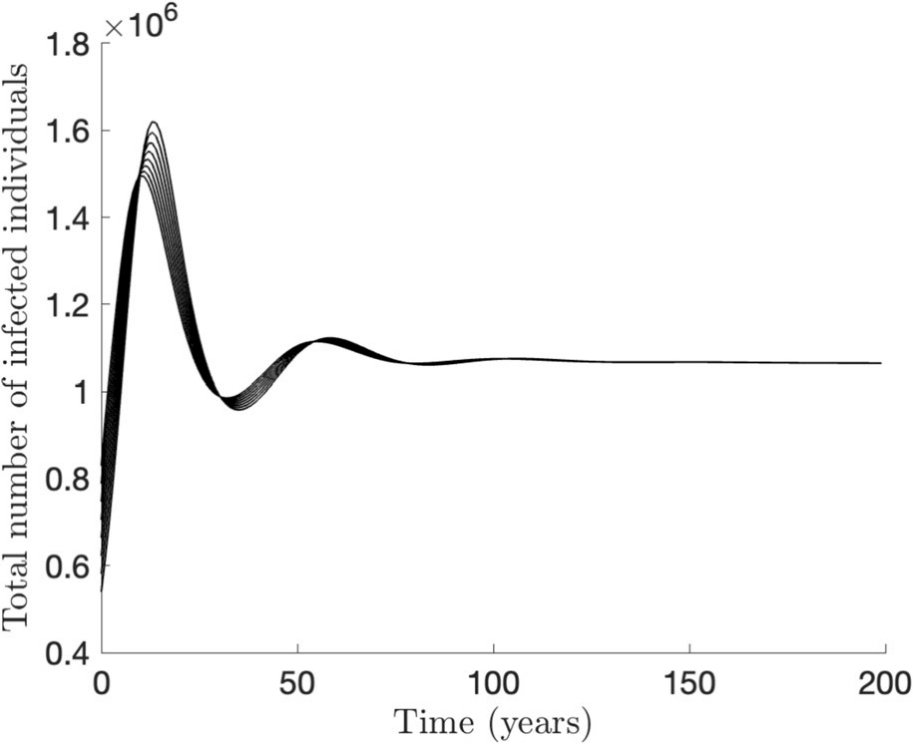
Simulations of the special case of the model (2.5) with $\delta = \delta _d = 0$ and $\varepsilon _v=1$, showing convergence of initial solutions to the unique endemic equilibrium when $\tilde{\mathcal {R}}_{vs}>1$. Parameter values used in the simulations are as given in Table 9, with $\delta = \delta _d = 0$ and $\varepsilon _v=1$ (so that, $\tilde{\mathcal {R}}_{vs}= 3.0212$).

The proof of Theorem 2.6, based on using a Krasnasolskii sub-linearity argument (Esteva and Vargas 2000; Hethcote and Thieme 1985; Thieme 1985), is given in Appendix D. The epidemiological implication of Theorem 2.6 is that, for the special case of the model (2.5) with $\delta =\delta _d=0$ and $\varepsilon _v=1$, HPV will persist in the community whenever $\tilde{\mathcal {R}}_{vs}>1$. The result of this theorem is illustrated in Figure 4, showing convergence of initial solutions to the unique endemic equilibrium when $\delta =\delta _d=0$, $\varepsilon _v=1$, and ${\tilde{\mathcal {R}}_{vs}}>1$.

## Calibration and Analysis of Simplified Model: No Screening

In order to fit the model (2.5) to available data and estimate its unknown parameters, a simplified version of the model with no Pap screening and with the three CIN and INM stages lumped into one CIN and INM compartment, respectively, is considered. The resulting model, tagged as the *simplified model*, is given by the following deterministic system of nonlinear differential equations:

3.1 $$\begin{aligned}&{Females} {\left\{ \begin{array}{ll} \dot{S}_f & =~ (1-q_f)\pi _f - (\lambda _{mf} + \xi _f + \mu ) S_f, \vspace{2mm} \\ \dot{V}_f & =~ q_f \pi _f + \xi _f S_f- (1-\varepsilon _v) \lambda _{mf} V_f - \mu V_f, \vspace{2mm} \\ \dot{E}_f & =~ \lambda _{mf} S_f + (1-\varepsilon _v) \lambda _{mf} V_f - (\sigma _f + \mu ) E_f, \vspace{2mm} \\ \dot{I}_f & =~ \sigma _f E_f - (\psi _f + \mu ) I_f, \vspace{2mm}\\ \dot{P}_f & =~ (1-b_f)\psi _f I_f - (\alpha _f + \mu )P_f, \vspace{2mm}\\ \dot{Q}_f & =~ (1-k_f)\alpha _f P_f -(\tilde{g}_f + \mu )Q_f, \vspace{2mm}\\ \dot{C}_f & =~ (1-d_f)\tilde{g}_f Q_f -(r_f + \tilde{\delta } + \mu ) C_f, \vspace{2mm} \\ \dot{R}_f^C & =~ r_f C_f - \mu R_f^C, \vspace{2mm}\\ \dot{R}_f & =~ b_f\psi _f I_f + k_f\alpha _f P_f +d_f\tilde{g}_fQ_f -\mu R_f,\\ \end{array}\right. }\nonumber \\&{Males} {\left\{ \begin{array}{ll} \dot{S}_m & =~ (1-q_m)\pi _m - (\lambda _{fm} +\xi _m+\mu ) S_m, \vspace{2mm}\\ \dot{V}_m & =~ q_m\pi _m +\xi _mS_m - (1-\varepsilon _v) \lambda _{fm} V_m - \mu V_m, \vspace{2mm}\\ \dot{E}_m & =~ \lambda _{fm} S_m+ (1-\varepsilon _v) \lambda _{fm} V_m - (\sigma _m +\mu )E_m, \vspace{2mm} \\ \dot{I}_m & =~ \sigma _m E_m - (\psi _m + \mu ) I_m, \vspace{2mm}\\ \dot{P}_m & =~ (1-b_m)\psi _m I_m - (\alpha _m + \mu )P_m, \vspace{2mm}\\ \dot{J}_m & =~ (1-k_m)\alpha _m P_m - (\tilde{g}_m + \mu )J_m, \vspace{2mm}\\ \dot{C}_m & =~ (1-\tilde{d}_m)\tilde{g}_m J_m - (r_m + \mu )C_m, \vspace{2mm}\\ \dot{R}_m^C & =~ r_mC_m -\mu R_m^C, \vspace{2mm}\\ \dot{R}_m & =~ b_m\psi _m I_m + k_m\alpha _m P_m +\tilde{d}_m\tilde{g}_mJ_m - \mu R_m, \end{array}\right. } \end{aligned}$$

where the forces of infection, $\lambda _{mf}$ and $\lambda _{fm}$, defined in (2.4), are now given by (note that the conservation law (2.3) is also applied in these expressions):

$$\begin{aligned}&\lambda _{mf} = \left( \beta _{mf}c_{mf}\right) \left[ \frac{I_m + \eta _m E_m + \tilde{\theta }_m (P_m + \tilde{\kappa }_mJ_m)}{N_m}\right] ,\\&~\lambda _{fm} = \left( \beta _{fm} c_{mf}\right) \left[ \frac{I_f + \eta _{f}E_f + \tilde{\theta }_{f} (P_f + \tilde{\kappa }_{f}Q_{f})}{N_m}\right] . \end{aligned}$$

The state variables of the simplified model (3.1) are as described in Table 1, except now that the compartments for females in the undetected and detected CIN stages ($Q_{iu}(t)$ and $Q_{id}(t)$, with $i = 1,2,3$) are lumped into the $Q_f(t)$ compartment of females having CIN. Similarly, the populations $C_u(t)$ and $C_d(t)$, of females having undetected and detected cervical cancer, are now lumped into the compartment $C_f(t)$ of females with cervical cancer. The compartments for males in the three INM stages, $J_{im}(t), i = 1,2,3$, are also lumped into the compartment $J_m(t)$ of males having INM. The parameters of the simplified model (3.1) are as described in Tables 2 and 3, with a few others described in Table 5.

**Table 5:** Description of parameters of the simplified model (3.1) with modified description.

Parameter	Description
$\tilde{\theta }_{f} (\tilde{\theta }_m)$	Modification parameter for infectiousness of $P_f, Q_{f} (P_m, J_m)$, in comparison to the infectiousness of $I_f (I_m)$
$\tilde{\kappa }_{f} (\tilde{\kappa }_m)$	Modification parameter for infectiousness of $Q_{f} (J_m)$ in comparison to $P_f (P_m)$
$\tilde{g}_f (\tilde{g}_m)$	Rate at which females (males) leave the CIN (INM) class
$r_f$	Recovery rate of cervical cancer for females
$\tilde{\delta }$	Mortality rate of females due to cervical cancer
$d_{f}$	Proportion of females with CIN that recover
$1-d_{f}$	Proportion of females with CIN that progress to cervical cancer
$\tilde{d}_m$	Proportion of males with INM that recover
$1-\tilde{d}_m$	Proportion of males with INM that progress to HPV-related cancer

Here, too, it can be shown (using the approach in Section 2.1) that all non-negative solutions of the simplified model (3.1) stay non-negative for all time *t*. Furthermore, using the approach in Section 2.1, the following region

$$\begin{aligned} \mathcal {D} = \mathcal {D}_f \times \mathcal {D}_m \subset \mathbb {R}_+^{9} \times \mathbb {R}_+^{9}, \end{aligned}$$

with, $\mathcal {D}_f =\{(S_f, V_f, E_f, I_f, P_f, Q_f, C_f, R_f^C, R_f)\in \mathbb {R}_+^{9}:0\le N_f\le \frac{\pi _f}{\mu }\}$ and $\mathcal {D}_m =\{(S_m, V_m, E_m, I_m, P_m, J_m, C_m, R^C_m, R_m) \in \mathbb {R}_+^{9} :0\le N_m\le \frac{\pi _m}{\mu }\}$, can be shown to be positively invariant with respect to the simplified model (3.1). Hence, the simplified model is well-posed in the region $\mathcal {D}$. The disease-free equilibrium of the simplified model (3.1) is given by:

$$\begin{aligned} \mathcal {E}_{0n} =(S_f^*,V_f^*,0,0,0,0,0,0,0,S_m^*,V_m^*,0,0,0,0,0,0,0), \end{aligned}$$

where, now,

3.2 $$\begin{aligned}&S_f^* = \frac{(1-q_f)\pi _f}{\xi _f + \mu }, \; V_{f}^* = \frac{\pi _f(\xi _f+\mu q_f)}{\mu (\xi _f+\mu )}, \; S_m^* = \frac{(1-q_m)\pi _m}{\xi _m+\mu }, \; \nonumber \\&V_m^* = \frac{\pi _m(\xi _m+\mu q_m)}{\mu (\xi _m+\mu )}. \end{aligned}$$

It is convenient to define the following quantity (the control reproduction number for the simplified model):

3.3 $$\begin{aligned} \mathcal {R}_{vn}= \sqrt{\mathcal {R}_{fn} \mathcal {R}_{mn}}, \end{aligned}$$

where,

$$\begin{aligned} \mathcal {R}_{fn}&= \frac{\beta _{f m} c_{mf}\mu p_m^*}{\pi _m (\sigma _f + \mu )}\left[ \eta _{f} + \frac{\sigma _f}{\psi _f + \mu } + \frac{\tilde{\theta }_{f} \sigma _f (1-b_f)\psi _f }{(\psi _f + \mu )(\alpha _f + \mu )} \right. \\&\quad \left. +\frac{\tilde{\theta }_{f} \tilde{\kappa }_{f} \sigma _f (1-b_f)\psi _f (1-k_f)\alpha _f}{(\psi _f + \mu )(\alpha _f + \mu )(\tilde{g}_f+\mu )}\right] , \\ \mathcal {R}_{mn}&= \frac{\beta _{m f} c_{mf}\mu p_f^*}{\pi _m (\sigma _m + \mu )}\left[ \eta _m + \frac{\sigma _m}{\psi _m + \mu } + \frac{\tilde{\theta }_m \sigma _m (1-b_m)\psi _m }{(\psi _m + \mu )(\alpha _m + \mu )} \right. \\&\quad \left. +\frac{\tilde{\theta }_m \tilde{\kappa }_m \sigma _m (1-b_m)\psi _m (1-k_m)\alpha _m}{(\psi _m + \mu )(\alpha _m + \mu )(\tilde{g}_m+\mu )}\right] , \end{aligned}$$

with, $p_f^* = \frac{\pi _f}{\mu } \bigg ( 1-\varepsilon _v \; \frac{\xi _f + \mu q_f}{\xi _f + \mu }\bigg )$ and $p_m^* = \frac{\pi _m}{\mu } \bigg ( 1-\varepsilon _v \; \frac{\xi _m + \mu q_m}{\xi _m + \mu }\bigg )$. The basic reproduction number of the simplified model is given by:

3.4 $$\begin{aligned} \mathcal {R}_{0n}=\mathcal {R}_{vn}|_{q_f=\xi _f=q_m=\xi _m=0}. \end{aligned}$$

The result below follows from Theorem 2 of (Van den Driessche and Watmough 2002) (see Appendix E.1 for detailed proof).

### Theorem 3.1

The *DFE*, $\mathcal {E}_{0n}$, of the simplified model (3.1) is locally-asymptotically stable if $\mathcal {R}_{vn}< 1$ and unstable if $\mathcal {R}_{vn}>1.$

This local asymptotic stability result (Theorem 3.1) is extended to the global asymptotic stability of the DFE of the special case of the simplified model with negligible disease-induced mortality (i.e., the model (3.1) with $\tilde{\delta }=0$). It is convenient to define the DFE of the special case of the simplified model (3.1) by $\tilde{\mathcal {E}}_{0n} = \mathcal {E}_{0n}|_{\tilde{\delta }=0}$. The following result will be needed in proving the global asymptotic stability of the DFE, $\tilde{\mathcal {E}}_{0n}$.

### Lemma 3.2

The region

$$\begin{aligned} \mathcal {D}^* =&~\{(S_f, V_f, E_f, I_f, P_f, Q_f, C_f, R_f^C, R_f, S_m, V_m, E_m, I_m, P_m, J_m, C_m, R^C_m, R_m) \in \mathcal {D} : \\ &~S_f \le S_f^*, V_f\le V_f^*, S_m\le S_m^*, V_m\le V_m^*\} \end{aligned}$$

is positively-invariant, and attracts all solutions of the simplified model (3.1) with $\tilde{\delta }=0$.

### Proof

Consider the special case of the simplified model (3.1) with $\tilde{\delta } = 0$. Substituting $\tilde{\delta } = 0$ into the equations for the rate of change of the total populations of females and males of the simplified model shows that $N_i(t) \rightarrow \frac{\pi _i}{\mu }$ as $t\rightarrow \infty$, for $i = f, m$, respectively. Thus, from now on, $N_i(t)$ will be replaced by their respective disease-free equilibrium values (or the respective maximum total populations in the absence of disease), $\frac{\pi _i}{\mu }$ (for $i=f,m$). Then, it follows from the first and 10th equations of the simplified model that for $i,j \in \{f, m\}$ and $i\ne j$,

$$\begin{aligned} \dot{S}_i&= (1-q_i)\pi _i - (\lambda _{ji} + \xi _i +\mu )S_i\\&\le (1-q_i)\pi _i - (\xi _i+\mu )S_i, \;\;\; \text {since}\;\;\; \lambda _{ji}\ge 0\\&= (\xi _i+\mu )(S_i^*-S_i), \end{aligned}$$

so that, by a standard comparison theorem (Lakshmikantham et al. 2016), $S_i(t) \le S_i(0)e^{-(\xi _i+\mu )t} +S_i^*[1-e^{-(\xi _i+\mu )t}]$. Thus,

3.5 $$\begin{aligned} S_i(t)\le S_i^*\;\;\;\text {for all}\;\;\;t\ge 0\;\;\;\text {provided that}\;\;\;S_i(0)\le S_i^*. \end{aligned}$$

Similarly, from the second and 11th equations of the simplified model (noting that $N_i(t)\le \frac{\pi _i}{\mu }$ in $\mathcal {D}$):

$$\begin{aligned} \dot{V}_i&= q_i\pi _i +\xi _i S_i -(1-\varepsilon _v)\lambda _{ji}V_i -\mu V_i\\&\le q_i\pi _i +\xi _i\left( \frac{\pi _i}{\mu }-V_i-E_i-I_i-P_i-Q_i-C_i-R_i^C-R_i\right) -\mu V_i\\&\le \frac{(\mu q_i +\xi _i)\pi _i}{\mu } -(\xi _i+\mu )V_i\\&= (\xi _i+\mu )(V_i^*-V_i). \end{aligned}$$

Hence, by the comparison theorem, $V_i(t) \le V_i(0) e^{-(\xi _i+\mu )t}+V_i^*[1-e^{-(\xi _i+\mu )t}],$ from which it follows that

3.6 $$\begin{aligned} V_i(t)\le V_i^*\;\;\;\text {for all}\;\;\;t\ge 0 \;\;\;\text {provided that}\;\;\;V_i(0)\le V_i^*. \end{aligned}$$

Hence, based on the bounds in (3.5) and (3.6), the region $\mathcal {D}^*$ is positively-invariant with respect to the flow generated by the special case of the simplified model (3.1) with $\tilde{\delta }=0$. In fact, it can also be shown that each solution in $\mathcal {D}$ either enters $\mathcal {D}^*$ in finite time or limits to $\tilde{\mathcal {E}}_{0n}$ (Gumel et al. 2006). $\square$

We claim the following result (the proof, which is based on using a standard comparison theorem (Lakshmikantham et al. 2016), is given in Appendix E.2):

### Theorem 3.3

The DFE, $\tilde{\mathcal {E}}_{0n}$, of the special case of the simplified model (3.1) with negligible disease-induced mortality (i.e., $\tilde{\delta }=0$) is globally-asymptotically stable in $\mathcal {D}^*$ whenever $\mathcal {R}_{vn}< 1$.

Unlike in the case of Theorem 3.1 (where the effective control of the disease when $\mathcal {R}_{vn}<1$ depends on the initial number of infected individuals being within the basin of attraction of the DFE of the simplified model), the epidemiological implication of Theorem 3.3 is that bringing the control reproduction number of the aforementioned special case of the simplified model below one is necessary and sufficient for the effective control of the disease, regardless of the size of the initial number of infected individuals.

### Data fitting and parameter estimation for the simplified model

Although the values of most of the parameters of the full model (2.5) are reliably known from the literature (as tabulated in Table 9) and demographic data from Korea, the values of some of the parameters are not known. These unknown parameters will be estimated by fitting the simplified version of the model (3.1) to the observed cumulative HPV-related cancer cases and mortality in the Republic of Korea, for both females and males, for the period from 1999 to 2020 (National Cancer Center Korea Central Cancer Registry 2022). For fitting purposes, it is assumed that males are not vaccinated (i.e., $q_m = \xi _m = 0$) and the vaccination coverage of females remains low ($q_f = 0.05$ per year, $\xi _f = 0.01$ per year (Choi et al. 2022)) before South Korea started the National Immunization Program (NIP) on HPV in 2016. Furthermore, since HPV is not the only cause of some cancer types (de Martel et al. 2017), an estimate for the average number of cancer cases attributable to HPV (needed for the data fitting) is obtained by taking the weighted average of cancer cases from different cancer sites with their HPV-attributable fraction given in Table 6.

It should be noted that, although the parameters related to disease progression and recovery in females are well-known in the literature, the corresponding parameters for males are unknown. In other words, although the values of the female progression and recovery parameters (i.e., $(1-b_f)\psi _f, (1-k_f)\alpha _f$, $(1-d_f)\tilde{g}_f$, $b_f\psi _f, k_f\alpha _f$, and $d_f\tilde{g}_f$) are known (and their baseline values are tabulated in Table 8), the values of the male-related progression and recovery parameters (i.e., $(1-b_m)\psi _m, (1-k_m)\alpha _m, (1-\tilde{d}_m)\tilde{g}_m$, $b_m\psi _m, k_m\alpha _m$, and $\tilde{d}_m\tilde{g}_m$) are unknown. These unknown male-related parameters will be estimated by fitting for the ratio of these rates for males, in relation to those for females (this is to take advantage of the fact that males recover faster from HPV infection and are less likely to have persistent infection than females (Giuliano et al. 2008a; National Cancer Center Korea Central Cancer Registry 2022; Moscicki et al. 2004, 1998; Shin et al. 2004; Van Doornum et al. 1994)). To do this, we assume that the ratio of the rate at which males progress from the $I_m$ class to the $P_m$ class (i.e., $(1-b_m)\psi _m$) to the rate at which females progress from the $I_f$ class to the $P_f$ class (i.e., $(1-b_f)\psi _f$), defined as $r_1 \in (0,1]$, equals the ratio for the progression rate of males from the $P_m$ class to the $J_m$ compartment (i.e., $(1-k_m)\alpha _m$) to the progression rate of females from the $P_f$ compartment to the $Q_f$ compartment (i.e., $(1-k_f)\alpha _f$). The justification for $r_1\in (0,1]$ stems from the fact that HPV prevalence in males is lower than in females (National Cancer Center Korea Central Cancer Registry 2022; Shin et al. 2004; Van Doornum et al. 1994). Similarly, we assume that this ratio is the same as that for the progression rate of males from the $J_m$ class to the $C_m$ class (i.e., $(1-\tilde{d}_m)\tilde{g}_m$) to that for the progression rate of females from the $Q_f$ class to the $C_f$ class (i.e., $(1-d_f)\tilde{g}_f$). That is, we set

3.7 $$\begin{aligned} \frac{(1-b_m)\psi _m}{(1-b_f)\psi _f} = \frac{(1-k_m)\alpha _m}{(1-k_f)\alpha _f} = \frac{(1-\tilde{d}_m)\tilde{g}_m}{(1-d_f)\tilde{g}_f} =: r_1. \end{aligned}$$

The objective then is to use the observed data for cumulative cancer cases and cancer mortality in South Korea (National Cancer Center Korea Central Cancer Registry 2022) to fit the simplified model for $r_1$ (this entails replacing every occurrence of the terms in the numerators of (3.7) in the simplified model (3.1) with their respective product of their denominators with $r_1$). In other words, once the fitted value of $r_1$ is obtained, equation (3.7) is then be used, together with the baseline values of the female-related parameters in Table 8, to obtain the estimated values of the three unknown male-related progression parameters (namely, $(1-b_m)\psi _m, (1-k_m)\alpha _m$, and $(1-\tilde{d}_m)\tilde{g}_m$). Similarly, it is assumed that the ratio of the recovery rate of males from the $I_m$ class (i.e., $b_m\psi _m$) to that of females from the $I_f$ class (i.e., $b_f\psi _f$), defined as $r_2 >1$, equals that for the recovery rate of males from the $P_m$ class (i.e., $k_m\alpha _m$) to that of females from the $P_f$ class (i.e., $k_f\alpha _f$). This ratio is also assumed to be the same as that for the recovery rate of males from the $J_m$ class (i.e., $\tilde{d}_m\tilde{g}_m$) to that of females from the $Q_f$ class (i.e., $d_f\tilde{g}_f$). In other words, we set

3.8 $$\begin{aligned} \frac{b_m\psi _m}{b_f\psi _f} = \frac{k_m\alpha _m}{k_f\alpha _f} = \frac{\tilde{d}_m\tilde{g}_m}{d_f\tilde{g}_f} =: r_2. \end{aligned}$$

Here, too, once the fitted value of $r_2$ is obtained, equation (3.8) can then be used, along with the baseline values of the female-related parameters in Table 8, to obtain estimated values of the three unknown male-related recovery parameters (namely, $b_m\psi _m, k_m\alpha _m$, and $\tilde{d}_m\tilde{g}_m$).

**Table 6:** HPV-attributable fraction by cancer sites (de Martel et al. 2017)

Cancer sites (ICD-10 code)	HPV-attributable fraction (%)	Cancer sites (ICD-10 code)	HPV-attributable fraction (%)
Cervix uteri (C53)	100	Oropharynx (C01, C09-10)	30.8
Anus (C21)	88	Vulva (C51)	24.9
Vagina (C52)	78	Larynx (C32)	2.4
Penis (C60)	50	Oral cavity (C02-06)	2.2

**Table 7:** Baseline values of estimated (fitted) parameters for the simplified model (3.1)

Parameter	Estimated Value	$95\%$ CI
$\beta _{mf}c_{mf}$	1.45 per year	(0.91, 1.48)
$\beta _{fm}c_{fm}$	2.5 per year	(2.49, 5.57)
$r_1$	0.42 (dimensionless)	(0.38, 0.44)
$r_2$	1.24 (dimensionless)	(1.21, 1.58)

Thus, the unknown parameters (or combination of parameters) of the simplified model (3.1) (and, by extension, the unknown parameters or combination of parameters for the full model (2.5)) to be estimated from the data fitting are:

- the effective transmission rate from infected males to susceptible females ($c_{mf}\beta _{mf})$;
- the effective transmission rate from infected females to susceptible males ($c_{fm}\beta _{fm}$);
- the ratio ($r_1\in (0, 1]$) of the progression rates of males (from $I_m$ to $P_m$, $P_m$ to $J_m$, and from $J_m$ to $C_m$) in comparison to progression rates of females (from $I_f$ to $P_f$, $P_f$ to $Q_f$, and from $Q_f$ to $C_f$). This allows us to estimate $(1-b_m)\psi _m, (1-k_m)\alpha _m$, and $(1-\tilde{d}_m)\tilde{g}_m$;
- the ratio ($r_2 >1$) of the recovery rates of males (from $I_m$, $P_m$, and $J_m$ to $R_m$) in comparison to recovery rates of females (from $I_f$, $P_f$, and $Q_f$ to $R_f$). This allows us to estimate $b_m\psi _m, k_m\alpha _m$, and $\tilde{d}_m\tilde{g}_m$.

The simplified model (3.1) is fitted to the data using a standard nonlinear least squares approach. Specifically, the "lsqcurvefit" function in-built in MATLAB is used to minimize the sum of the squared differences between each observed data point (for the cumulative cancer cases and mortality) and the corresponding cumulative cancer cases and mortality projection obtained from the simulation of the simplified model (3.1). The baseline values of the aforementioned eight unknown parameters obtained from the data fitting, together with their corresponding 95% confidence intervals, are tabulated in Table 7.

**Fig. 5:**
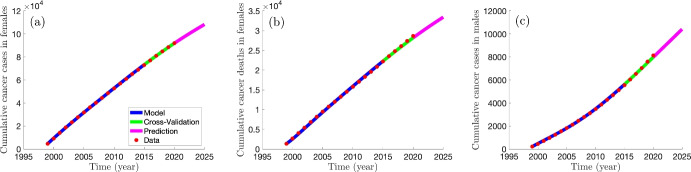
Data fitting of the simplified model (3.1), using cumulative data on the incidence and mortality of HPV-related cancers in females and males in Korea from 1999 to 2020 (National Cancer Center Korea Central Cancer Registry 2022). (a) Cumulative cervical cancer cases in females, (b) Cumulative cervical cancer mortality in females, (c) Cumulative HPV-related cancer cases in males. The first 17 years of data are used for data fitting, the next 5 years of data are used for cross-validation, and the calibrated model (3.1) predicts the next 5 years.

**Fig. 6:**
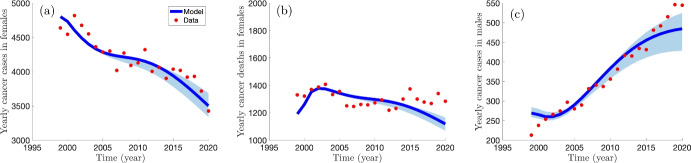
Simulation of the calibrated model (3.1) using parameters estimated from the cumulative data on the incidence and mortality of HPV-related cancers in females and males in Korea (National Cancer Center Korea Central Cancer Registry 2022). The fixed and fitted parameter values used are as given in Table 8. The shaded regions represent 95% confidence bands propagated from the bootstrap samples of fitted parameters in Table 7. (a) Annual cervical cancer cases in females, (b) Annual cervical cancer mortality in females, (c) Annual HPV-related cancer cases in males.

The results of the data fitting of the simplified model is depicted in Figure 5, showing a good fit ($R^2 = 0.99997$). The goodness of the fitting is further corroborated by simulating the simplified model (3.1) using the estimated and fixed parameters (in Tables 7 and 8) to predict the yearly cancer cases and mortality in both females and males, and compare these with the corresponding observed data. The results obtained, depicted in Figure 6, also show a very good prediction. It should be stated that, using the baseline values of the fixed and estimated parameters given in Tables 7 and 8, Equations (3.3) and (3.4) show that the vaccination reproduction number ($\mathcal {R}_{vn}$) and the basic reproduction number ($\mathcal {R}_{0n}$) of the simplified model (3.1) take the values $\mathcal {R}_{vn}= 3.1206$, and $\mathcal {R}_{0n} = 4.0389$, respectively. Thus, this study shows that the pre-NIP vaccination coverage level (which is what we used to fit the simplified model) will not lead to the effective control of HPV in Korea (since it fails to bring $\mathcal {R}_{vn}$ to a value less than one).

The baseline values for the fixed and estimated parameters of the simplified model (given in Tables 7 and 8) are used to acquire the baseline values of the remaining parameters of the full model (2.5), as tabulated in Table 9. For instance, the detection rates of females with CIN1, CIN2, and CIN3 (denoted by $d_{u3}g_f$, $h_{u3}y_f$, and $q_{u3}z_f$, respectively) are obtained by multiplying the Pap screening coverage (assumed to be 61% (Shin et al. 2022)) with sensitivity of the Pap test (assumed to be 60% for CIN1 and 78% for CIN2/CIN3 (Van de Velde et al. 2007)), and dividing by the frequency of the Pap test (which, in Korea, is recommended to be every two years for females of age 20 to 74 (Shin et al. 2022)).

**Table 8:** Baseline values of the parameters for the simplified model (3.1)

Parameter	Baseline Value (per year)	Source / Comment
$\pi _f$, $\pi _m$	358000, 372000	(Statistics Korea 2000)
$\mu$	1/65	(Malik et al. 2013)
$q_f$, $\xi _f$	0.05 (dimensionless), 0.01	(Choi et al. 2022)
$q_m$, $\xi _m$	0 (dimensionless), 0	Assumed
$c_{mf}$	2	(Podder and Gumel 2009)
$c_{fm}$	$\frac{c_{mf}N_{f}(t)}{N_m(t)} = \frac{2N_{f}(t)}{N_m(t)}$	From the value of $c_{mf}$ above
$\beta _{mf}$	0.72 (dimensionless)	Derived from (Podder and Gumel 2009)
$\beta _{fm}$	$\frac{\beta _{fm}c_{fm}}{c_{fm}(t)} = \frac{2.5}{c_{fm}(t)}$ (dimensionless)	Derived from (Podder and Gumel 2009)
$\eta _{f}, \eta _m$	0.5 (dimensionless)	(Javame and Gumel 2016)
$\tilde{\theta }_{f}, \tilde{\theta }_m$	0.9 (dimensionless)	(Javame and Gumel 2016)
$\tilde{\kappa }_{f}, \tilde{\kappa }_m$	1.5 (dimensionless)	(Javame and Gumel 2016)
$\varepsilon _v$	0.95 (dimensionless)	(Malik et al. 2013)
$\sigma _f, \sigma _m$	3	(Javame and Gumel 2016)
$(1-b_f)\psi _f$	0.017	(Javame and Gumel 2016)
$(1-b_m)\psi _m = r_1 (1-b_f)\psi _f$	0.0076	(Javame and Gumel 2016) and fitted value of $r_1$
$(1-k_f)\alpha _f$	0.07	(Javame and Gumel 2016)
$(1-k_m)\alpha _m = r_1 (1-k_f)\alpha _f$	0.03	(Javame and Gumel 2016) and fitted value of $r_1$
$(1-d_f)\tilde{g}_f$	0.14	(Malik et al. 2013)
$(1-\tilde{d}_m)\tilde{g}_m = r_1(1-d_f)\tilde{g}_f$	0.06	(Malik et al. 2013) and fitted value of $r_1$
$b_f\psi _f$	0.495	(Malik et al. 2013)
$b_m\psi _m = r_2 b_f\psi _f$	0.625	(Malik et al. 2013) and fitted value of $r_2$
$k_f\alpha _f$	0.05	(Javame and Gumel 2016)
$k_m\alpha _m = r_2 k_f\alpha _f$	0.063	(Javame and Gumel 2016) and fitted value of $r_2$
$d_f\tilde{g}_f$	0.01	(Javame and Gumel 2016)
$\tilde{d}_m\tilde{g}_m = r_2d_f\tilde{g}_f$	0.013	(Javame and Gumel 2016) and fitted value of $r_2$
$r_f$	0.5	(Javame and Gumel 2016)
$r_m = r_2 r_f$	0.63	(Javame and Gumel 2016) and fitted value of $r_2$
$\tilde{\delta }$	0.23	(Ministry of Health and Welfare 2021)

**Table 9:** Baseline values of the parameters for the full model (2.5)

Parameter	Baseline Value (per year)	Source
$\pi _f$, $\pi _m$	358000, 372000	(Statistics Korea 2000)
$\mu$	1/65	(Malik et al. 2013)
$q_f$, $\xi _f$	0.05 (dimensionless), 0.01	(Choi et al. 2022)
$q_m$, $\xi _m$	0 (dimensionless), 0	Assumed
$c_{mf}$	2	(Podder and Gumel 2009)
$c_{fm}$	$\frac{c_{mf}N_{f}(t)}{N_m(t)} = \frac{2N_{f}(t)}{N_m(t)}$	From value of $c_{mf}$ above
$\beta _{mf}$	0.72 (dimensionless)	Derived from (Podder and Gumel 2009)
$\beta _{fm}$	$\frac{\beta _{fm}c_{fm}}{c_{fm}(t)} = \frac{2.5}{c_{fm}(t)}$ (dimensionless)	Derived from (Podder and Gumel 2009)
$\eta _{f}, \eta _m$	0.5 (dimensionless)	(Javame and Gumel 2016)
$\theta _{f}, \theta _m$	0.9 (dimensionless)	(Javame and Gumel 2016)
$\kappa _{f}, \kappa _m$	1.5 (dimensionless)	(Javame and Gumel 2016)
$\nu$	0.05 (dimensionless)	Assumed
$\kappa _{fd}$	1.5 (dimensionless)	(Javame and Gumel 2016)
$\varepsilon _v$	0.95 (dimensionless)	(Malik et al. 2013)
$\sigma _f, \sigma _m$	3	(Javame and Gumel 2016)
$(1-b_f)\psi _f$	0.017	(Javame and Gumel 2016)
$(1-b_m)\psi _m = r_1 (1-b_f)\psi _f$	0.0076	(Javame and Gumel 2016) and fitted value of $r_1$
$(1-k_f)\alpha _f$	0.07	(Javame and Gumel 2016)
$(1-k_m)\alpha _m = r_1 (1-k_f)\alpha _f$	0.03	(Javame and Gumel 2016) and fitted value of $r_1$
$(1-d_{u1}-d_{u3})g_f$	0.33	Derived from model (3.1)
$(1-d_{d1})\zeta _1$	0.14	(Elbasha et al. 2007)
$(1-d_m)g_m = r_1 (1-d_{u1}-d_{u3})g_f$	0.14	Derived from model (3.1) and fitted value of $r_1$
$(1-h_{u1}-h_{u2}-h_{u3})y_f$	0.33	Derived from model (3.1)
$(1-h_{d1}-h_{d2})\zeta _2$	0.14	(Elbasha et al. 2007)
$(1-h_{m1}-h_{m2})y_m = r_1 (1-h_{u1}-h_{u2}-h_{u3})y_f$	0.14	Derived from model (3.1) and fitted value of $r_1$
$(1-q_{u1}-q_{u2}-q_{u3})z_f$	0.98	Derived from model (3.1)
$(1-q_{d1}-q_{d2})\zeta _3$	0.42	(Elbasha et al. 2007)
$(1-q_{m1}-q_{m2})z_m = r_1 (1-q_{u1}-q_{u2}-q_{u3})z_f$	0.43	Derived from model (3.1) and fitted value of $r_1$
$h_{u2}y_f, h_{d2}\zeta _2$	0.133	(Elbasha et al. 2007)
$h_{m2}y_m$	0.133	(Elbasha et al. 2007)
$q_{u2}z_f, q_{d2}\zeta _3$	0.03	(Elbasha et al. 2007)
$q_{m2}z_m$	0.03	(Elbasha et al. 2007)
$d_{u3}g_f$, $h_{u3}y_f$, $q_{u3}z_f$	0.18, 0.24, 0.24	Derived from (Shin et al. 2022; Malik et al. 2013)
$w_f$	0.24	Derived from (Shin et al. 2022; Malik et al. 2013)
$b_f\psi _f$	0.495	(Malik et al. 2013)
$b_m\psi _m = r_2 b_f\psi _f$	0.625	(Malik et al. 2013) and fitted value of $r_2$
$k_f\alpha _f$	0.05	(Javame and Gumel 2016)
$k_m\alpha _m = r_2 k_f\alpha _f$	0.063	(Javame and Gumel 2016) and fitted value of $r_2$
$d_{u1}g_f, h_{u1}y_f, q_{u1}z_f$	0.01	(Javame and Gumel 2016)
$d_{d1}\zeta _1, h_{d1}\zeta _2, q_{d1}\zeta _3$	0.13	(Malik et al. 2013)
$d_mg_m = r_2 d_{u1}g_f, h_{m1}y_m = r_2 h_{u1}y_f, q_{m1}z_m = r_2 q_{u1}z_f$	0.013	(Javame and Gumel 2016) and fitted value of $r_2$
$r_u, r_d$	0.5, 0.63	(Javame and Gumel 2016)
$r_m = r_2 r_u$	0.63	(Javame and Gumel 2016) and fitted value of $r_2$
$\delta , \delta _d$	0.23, 0.023	(Ministry of Health and Welfare 2021)

## Sensitivity Analysis for the Model (2.5)

The model (2.5) contains 62 parameters. Although the baseline values of these parameters are known (either available directly from the literature, or are obtained by fitting the simplified model (3.1) to the available data, as tabulated in Table 9), uncertainties are expected to arise in the estimate of their values. Therefore, it is crucial to evaluate the impact of these uncertainties on the overall outcome of the numerical simulations of the model, and determine which parameters have the most influence on a chosen response function (Blower and Dowlatabadi 1994; Marino et al. 2008; Safdar et al. 2022). The sensitivity analysis entails defining distributions of each parameter and finding the partial rank correlation coefficients (PRCCs) for each parameter in relation to the response function (Blower and Dowlatabadi 1994; Marino et al. 2008; Safdar et al. 2022). PRCC values range from $-1$ to 1, and a higher magnitude of the PRCC value (typically greater or equal to 0.5 in magnitude) implies a stronger correlation with the response function (Safdar et al. 2022). In addition, parameters with positive PRCC values are positively correlated, while those with negative values are negatively correlated with the response function. To implement the method, a response function is chosen first of all (specifically, the control reproduction number $\mathcal {R}_{vs}$ is chosen as the response function), and distribution of each parameter is chosen as uniform distribution with range defined by taking 20% to the left and 20% to the right of the baseline values given in Table 9 (Safdar et al. 2022). It should be mentioned that, since the response function (i.e., the control reproduction number, $\mathcal {R}_{vs}$) contains 56 of the 62 parameters of the model (2.5), the remaining six parameters of the model (namely, $w_f, r_u, r_d, r_m, \delta , \delta _d$) do not feature in the sensitivity analysis with respect to $\mathcal {R}_{vs}$. Moreover, the factor fixing approach (Saltelli 2008) was used to determine the parameters that are not influential to the value of $\mathcal {R}_{vs}$. This analysis revealed that the value of $\mathcal {R}_{vs}$ is not sensitive to 31 of the 56 parameters in $\mathcal {R}_{vs}$. Consequently, the sensitivity analysis was conducted for the remaining 25 parameters, after the monotonicity of $\mathcal {R}_{vs}$ was confirmed in each of them by generating respective scatter plots of $\mathcal {R}_{vs}$ as a function of each of the parameters (as depicted in Figure 12 in Appendix F). Specifically, the parameter ranges are divided into 1, 000 sub-intervals of equal length, and parameter sets are chosen from the sub-intervals without replacement. This leads to a $1,000 \times 25$ matrix, and each row of the matrix is used to calculate the response function $\mathcal {R}_{vs}$. Then, the Pearson correlation coefficient is calculated for each parameter (which implies the degree of influence of each parameter on the response function in the absence of the influence of the other parameters) (Blower and Dowlatabadi 1994; Marino et al. 2008).

**Fig. 7:**
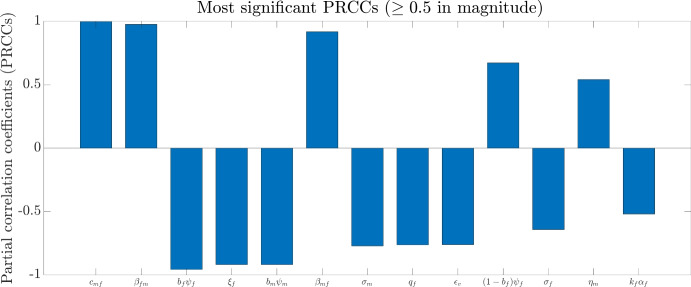
Partial rank correlation coefficients (PRCCs) of the most significant coefficients (with $|\text{ PRCC }|\ge 0.5$) in the chosen response function ($\mathcal {R}_{vs}$) of the model (2.5). The baseline values of the parameters used are as given in Table 9, and their ranges are taken to be 20% to the left and 20% to the right of their baseline values, assuming a uniform distribution.

Figure 7 depicts the PRCC values of the most influential (i.e., PRCC value greater or equal to 0.5 in magnitude) parameters of the response function obtained from the sensitivity analysis. This figure shows that the top-six parameters that have the highest impact on the response function ($\mathcal {R}_{vs}$) are:

- the average number of sexual partners for females per year ($c_{mf}$; with PRCC = +0.997). It should be stated that the parameter for the average number of sexual partners for males per year ($c_{fm}$) is also highly correlated with the response function, since it is proportional to $c_{mf}$, by the conservation law (2.3).
- the transmission probability of HPV from infected females to susceptible males ($\beta _{fm}$; with PRCC = +0.976).
- the recovery rate of symptomatic infectious females from HPV infection ($b_f\psi _f$; with PRCC $= -0.959$).
- the catch-up vaccination rate of females ($\xi _f$; with PRCC $=-0.919$).
- the recovery rate of symptomatic infectious males from HPV infection ($b_m\psi _m$; with PRCC $= -0.918$).
- the transmission probability of HPV from infected males to susceptible females ($\beta _{mf}$; with PRCC $= +0.917$).

These sensitivity analysis results indicate that, with respect to the reproduction number ($\mathcal {R}_{vs}$) as the response function, the parameters related to HPV transmission (i.e., $c_{mf}, c_{fm}, \beta _{fm}, \beta _{mf}$), recovery of symptomatic infectious individuals (i.e., $b_f\psi _f, b_m\psi _m$), and vaccination (i.e., $\xi _f$) have the most influence on the control reproduction number $\mathcal {R}_{vs}$ (hence, on the burden of HPV and HPV-related cancers in Korea). Thus, public health control and mitigation efforts against HPV infection and associated cancers should be targeted towards these highly influential parameters (i.e., increasing (decreasing) their value if their PRCC is negative (positive)). Specifically:

(i) the values of the transmission-related parameters ($c_{mf}, c_{fm}, \beta _{fm},$ and $\beta _{mf}$) can be decreased by implementing public health strategies that encourage safer sexual practices, such as condom use (which reduces $\beta _{fm}$ and $\beta _{mf}$), and reduction of average number of sexual partners (which reduces $c_{mf}$ and $c_{fm}$).
(ii) the values of the recovery-related parameters ($b_f\psi _f, b_m\psi _m$, and also $k_f\alpha _f$) can be increased by increasing the testing, detection and treatment of individuals with clinical symptoms of HPV.
(iii) the values of the vaccine-related parameters ($\xi _f$, and also $q_f, \varepsilon _v$) can be increased by increasing the coverage of the HPV vaccine among the target population (the efficacy of both the Cervarix^®^ and Gardasil^®^ vaccine are already very high (Cheng et al. 2020)).

It should be mentioned, based on the PRCC values in Figure 7, that other parameters, such as the rate of symptom development of males ($\sigma _m$, with PRCC = -0.773), the progression rate of infectious symptomatic females to persistent infection ($(1-b_f)\psi _f$, with PRCC = +0.671), the rate of symptom development of females ($\sigma _f$, with PRCC = -0.644), and the modification parameter for the infectiousness of pre-symptomatic males compared to symptomatic males ($\eta _m$, with PRCC = +0.540), are also highly correlated to the response function, $\mathcal {R}_{vs}$.

## Numerical Simulation and Assessment of Control Strategies

The full model (2.5) will now be simulated, using the estimated and fixed parameters (given in Tables 7 and 9), to assess the population-level impact of the combined vaccination and Pap screening strategies currently being implemented in an effort to effectively control the spread of HPV and related cancers in Korea. The impact of other extended versions of these strategies will also be assessed. Specifically, the following strategies will be assessed:

- *Strategy A*: This strategy is based on the post-NIP vaccination strategy (which entails the routine annual vaccination of 80% of girls in the 12-17 year age group), the annual catch-up vaccination of 30,000 females in the 18-26 year age group (which corresponds to the baseline catch-up vaccination coverage of females, calculated using the baseline value of the catch-up vaccination rate parameter for females, $\xi _f$, given in Table 9), and the routine Pap screening strategy (which involves the cervical cancer screening of 61% of adult females in the age group 20-74 (Min et al. 2015)). This strategy does not include the vaccination of boys or males.
- *Strategy B*: This is the same as Strategy A (and boys and males are not vaccinated), but with the vaccination coverage for girls increased to 95% (as against the 80% in Strategy A) and the vaccination coverage for the 18-26 year old females increased to 175,000 (as against 30,000 in Strategy A). This increase allows the vaccination coverage of females at the disease-free equilibrium $\left( v_f^* = \frac{\xi _f+\mu q_f}{\xi _f+\mu }\right)$, obtained by solving for $v_f^*$ from equation (2.12) for the case when $v_m^*=0$, to reach the herd immunity threshold ($v_f^*=0.99$, as described in Section 2.2.1).
- *Strategy C*: This is the same as Strategy A, but with the annual routine vaccination of 80% of boys in the 12-17 year age group (the catch-up vaccination rate for males, $\xi _m$, is set to zero in the numerical simulations).

Simulations will also be carried out for the aforementioned three strategies with the Pap screening coverage increased to 90%.

### Disease burden corresponding to each strategy

The control reproduction number, $\mathcal {R}_{vs}$, corresponding to each of the three strategies are tabulated in Table 10, for the cases where Pap screening is at 61% baseline and when it is increased to 90%. It follows from this table that, while the reproduction numbers for Strategies B and C are below one, the reproduction number for Strategy A is above one. In other words, based on the parameter values (tabulated in Table 9 and the caption of Table 10) used in these simulations, HPV can be eliminated under Strategies B and C, but will persist under Strategy A. Thus, the increase in the annual vaccination coverage for girls (from the current post-NIP baseline of 80% to 95%) combined with the increase in the catch-up vaccination coverage for females (from 30,000 to 175,000), corresponding to Strategy B, will significantly alter the trajectory and burden of the disease in Korea (from persistence, as shown for Strategy A, to possible elimination). Hence, extending the current post-NIP routine vaccination in Korea to also include catch-up vaccination for females of ages 18-26 will strongly enhance HPV elimination prospect in Korea. Similarly, Table 10 shows that the additional vaccination of 80% of boys (corresponding to Strategy C) can change the trajectory and burden from persistence (for Strategy A) to possible elimination. Here, too, extending the post-NIP vaccination strategy to include the vaccination of boys will significantly enhance the aforementioned elimination prospect. These increases in vaccination coverage (for girls, adult females, and even boys) are generally realistically-attainable in Korea (considering the high coverages for infant vaccination series covered by the NIP in Korea (KCDC Center for Infectious Disease Control 2023)). Table 10 also shows that increasing the screening coverage from the 61% baseline to 90% only has marginal impact in the values of the reproduction numbers for the three strategies (this may be due to the assumption in the model that screened or detected HPV-infected individuals have reduced transmissibility, compared to undetected individuals).

**Table 10:** Values of the control reproduction number, $\mathcal {R}_{vs}$, of the model (2.5) based on (a) Strategy A ($q_f = 0.8$), (b) Strategy B ($q_f = 0.95, ~\xi _f = 0.06$), and (c) Strategy C ($q_f =q_m = 0.8$), under (i) baseline Pap screening coverage of 61% and (ii) increased Pap screening coverage of 90% ($d_{u3}g_f=0.27,~ h_{u3}y_f=q_{u3}z_f=w_f=0.35$). The rest of the parameter values used are as given Table 9.

$\mathcal {R}_{vs}$	(a) Strategy A	(b) Strategy B	(c) Strategy C
(*i*) Baseline Pap screening coverage (61%)	1.6181	0.9728	0.7927
(*ii*) Increased Pap screening coverage (90%)	1.6134	0.97	0.7904

Although the National Immunization Program for HPV in Korea started in 2016 (providing routine vaccination to 12-year old girls) (Kwak and Hwang 2024), its real effect did not begin to be felt in the population until the vaccinated girls reach the age of commencement of sexual activity (which is assumed to be 17 years in Korea). Consequently, the simulations to be carried out in the study will start in the year 2021 (when the vaccinated girls have started to become sexually-active).

**Fig. 8:**
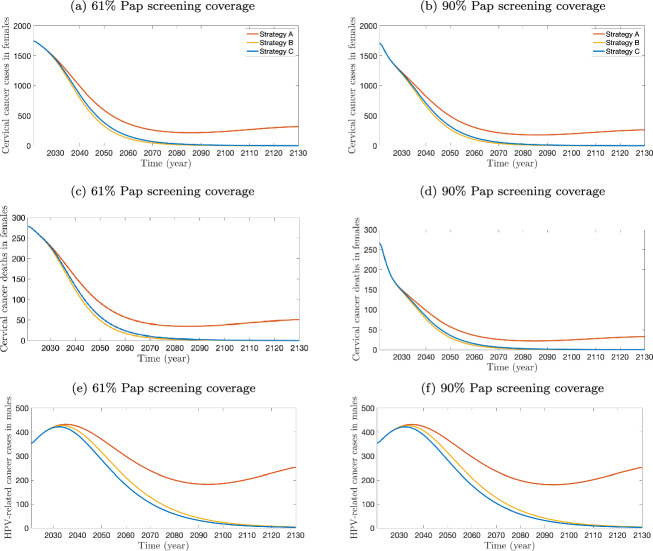
Simulation of the model (2.5) to assess the combined impact of Pap screening and vaccination (of girls, females, and boys) on the burden of cervical cancer in females and HPV-related cancers in males, for the aforementioned Strategies A, B, and C and varying Pap screening coverages. (a) Yearly cervical cancer cases in females as a function of time from 2021 to 2130 for Pap screening coverage at 61%, (b) Yearly cervical cancer cases in females from 2021 to 2130 for Pap screening coverage at 90%, (c) Yearly cervical cancer-induced deaths in females from 2021 to 2130 for Pap screening coverage at 61%, (d) Yearly cervical cancer-induced deaths in females from 2021 to 2130 for Pap screening coverage at 90%, (e) Yearly HPV-related cancer cases in males from 2021 to 2130 for Pap screening coverage at 61%, (f) Yearly HPV-related cancer cases in males from 2021 to 2130 for Pap screening coverage at 90%. Parameter values used in these simulations are as given in Table 9, but with the following changes: (i) Strategy A: $q_f = 0.8$, (ii) Strategy B: $q_f=0.95$, $\xi _f=0.06$ per year, (iii) Strategy C: $q_f=q_m=0.8$. In all simulations for the 90% Pap screening coverage, the following values were used: $d_{u3}g_f=0.27,~ h_{u3}y_f=q_{u3}z_f=w_f=0.35$ per year.

### Impact of vaccination and pap screening on cancer cases and mortality

The first set of simulations carried out are for assessing the impact of Pap screening and vaccination (of girls, females, and boys; corresponding to Strategies A, B, and C, as described above) on the yearly incidence and mortality caused by HPV-related cancers in Korea for the period between 2021 and 2130. These simulation results, obtained using the parameter values in Table 9 and the caption of Figure 8 to simulate the model (2.5), show a pronounced decline in the number of cervical cancer cases and mortality in females (for any of the three strategies) under both the baseline (61%) and improved (90%) Pap screening coverages, as depicted in Figure 8 (subplots (a)-(d)). Specifically, Figure 8 shows that while Strategy A induces the highest cervical cancer burden in females (red curve in subplots (a)-(d)), Strategies B and C incur much lower (and nearly the same) burden over the same period. These figures also show that vaccinating boys at 80% coverage (Strategy C) induces very strong reduction in cervical cancer burden in females, at a level almost the same as the reduction recorded under Strategy B, which entails allocating all the vaccine resources to girls and females only (compare the blue and gold curves in subplots (a)-(d)). These results suggest a strong *spillover benefit* (i.e., the vaccination of boys reduces the incidence and mortality due to cervical cancer in females). Furthermore, this figure shows that although the increase in Pap screening coverage (from 61% to 90%) has only marginal effect on the incidence of cervical cancer (compare subplots (a) and (b)), such an increase has a significant impact in reducing cervical cancer mortality (compare subplots (c) and (d)). It should be noted from these plots that while the burden of cervical cancer persists under Strategy A, such burden dies out with time under Strategies B and C (this is because the control reproduction number for Strategy A exceeds unity, while those for Strategies B and C are below unity, as tabulated in Table 10; the time to elimination of the HPV burden, for Strategies B and C, are given in Table 11).

The simulations depicted in Figure 8 also show that while the number of HPV-related cancer cases in males initially increases during the first 10-15 years (for any of the three strategies), it sharply declines reaching elimination levels, except for Strategy A, which rebounds after about 70 years of implementation (see subplots (e) and (f) in Figure 8). This figure further shows that vaccinating girls and females has a significant spillover effect in reducing the burden of HPV-related cancers in males (see red and gold curves in subplots (e) and (f)). In these subplots, Strategy C consistently does better than Strategy B for most of the simulation duration, suggesting that if the objective is to reduce cancer cases in males, then allocating more vaccination resources to boys (Strategy C) would be more effective than allocating these resources to girls and females (Strategy B). Furthermore, this figure shows that increasing Pap screening coverage of females (from 61% to 90%) induces very marginal spillover effect on the incidence of HPV-related cancers in males (compare Figure 8 subplots (e) and (f)).

**Fig. 9:**
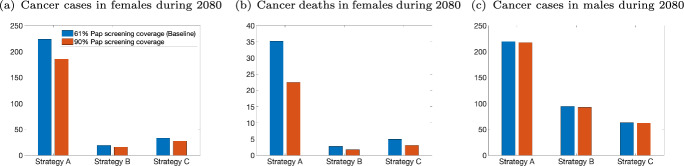
Simulation of the model (2.5) to assess the combined impact of Pap screening and vaccination (of girls, females, and boys) on the burden of HPV-related cancers in females and males during the year 2080, for Strategies A, B, and C and varying Pap screening coverages. The panels depict the number of (a) cervical cancer cases in females, (b) cervical cancer-induced deaths in females, and (c) HPV-related cancer cases in males, during 2080. Parameter values used in these simulations are as given in Table 9, but with the following changes: (i) Strategy A: $q_f = 0.8$, (ii) Strategy B: $q_f=0.95$, $\xi _f=0.06$ per year, (iii) Strategy C: $q_f=q_m=0.8$. In all simulations for the 90% Pap screening coverage, the following values were used: $d_{u3}g_f=0.27,~ h_{u3}y_f=q_{u3}z_f=w_f=0.35$ per year.

**Fig. 10:**
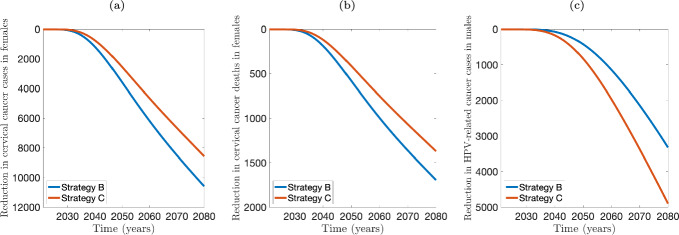
Simulation of the model (2.5) to assess the combined impact of Pap screening and vaccination (of girls, females, and boys) on the burden of HPV-related cancers in females and males in Korea by the year 2080, for Strategies B and C in comparison to Strategy A under baseline Pap screening coverage. The panels show reduction in the cumulative number of (a) cervical cancer cases in females, (b) cervical cancer-induced deaths in females, and (c) HPV-related cancer cases in males, by 2080. Parameter values used in these simulations are as given in Table 9, but with the following changes: (i) Strategy A: $q_f = 0.8$, (ii) Strategy B: $q_f=0.95$, $\xi _f=0.06$ per year, (iii) Strategy C: $q_f=q_m=0.8$.

Overall, the simulations for the impact of Pap screening and vaccination strategies implemented in Korea (depicted in Figure 8) show that increasing the vaccination coverage for girls and females (Strategy B) or the vaccination of boys at 80% coverage (Strategy C) will have a significant effect in reducing (and eliminating) cervical cancer burden in Korea (with Strategy B mostly marginally better than Strategy C, particularly during the first 60 years of implementation), and that vaccinating boys induces strong spillover effect in reducing the cervical cancer burden in females (subplots (a)-(d) of Figure 8). Furthermore, increasing the vaccination coverage for girls and females (Strategy B) or the vaccination of boys at 80% coverage (Strategy C) will have a significant effect in reducing (and eliminating) HPV-related cancer burden in males in Korea (with Strategy C always marginally better than Strategy B), and that vaccinating girls and females induces a strong spillover effect in reducing the HPV-related cancer burden in males (subplots (e) and (f) of Figure 8). The results in Figure 8 are also displayed using bar charts in Figure 9, showing similar trends and profiles, but also allowing for clear comparison of the impact between baseline (61%) and improved (90%) Pap screening coverage under each strategy. It should also be mentioned that similar trends (of the aforementioned simulation results in Figure 8 or Figure 9, for the yearly incidence and mortality of HPV-related cancers in Korea) are observed with respect to the reductions in the cumulative incidence and mortality for the HPV-related cancers, when Strategies B and C are compared with the prevailing Strategy A in Korea (as depicted in Figure 10). The reductions in the cumulative incidence and mortality for HPV-related cancers are also depicted for various vaccination coverages for boys (set at 25%, 50%, and 75%) in addition to Strategy A, and the results (depicted in Figure 13 of Appendix G.1) showed lower reductions in disease burden (compare Figure 13 with Figure 10). Similarly, simulations for Strategy A with increased vaccination of girls (set at 85%, 90%, 95%) show greater reductions in cancer incidence and mortality compared to Strategy A (compare Figure 14 in Appendix G.2 with Figure 10).

Since the simulations in Figure 8 show convergence to the disease-free equilibrium under strategies B and C (which is also in line with the corresponding results in Table 10, showing that the reproduction number, $\mathcal {R}_{vs}$, for each of the two strategies is always less than one, so that, by Theorem 2.3, the disease will die out if the initial number of HPV-infected individuals in the community is low enough), it is instructive to determine the expected *time-to-elimination* associated with the implementation of these strategies (B and C) under the long-term scenario. For simulation purposes, we consider elimination to occur when the total number of cervical cancer cases in Korea is less than 10. This figure is much lower than the figure corresponding to the World Health Organization’s elimination standard of four cervical cancer cases per 100,000 females (World Health Organization 2020) (which translates to 1,020 cervical cancer cases in Korea). Table 11 depicts the simulation results of the model (2.5), under the long-term scenario, showing the first time the total number of females with cervical cancer in Korea fall below 10. It follows from this table that cervical cancer elimination could be achieved faster under Strategy B with improved Pap screening coverage (with such elimination achieved by 2085) than under Strategy B with baseline Pap screening coverage (this achieves elimination by 2088) or Strategy C (which achieves elimination by 2097 or 2094 for baseline and improved Pap screening coverages, respectively). In other words, this study shows that, based on the parameter values used in our simulations, the combined anti-HPV vaccination and Pap screening strategy can lead to the elimination of cervical cancer in Korea in about 60 years if the routine vaccination coverage for girls (12-17 years of age) and the catch-up vaccination coverage for females (18-26 years of age), are increased from their current levels of 80% and 30,000 per year, respectively, to 95% and 175, 000 per year, respectively.

**Table 11:** Simulation of the model (2.5), showing time-to-elimination of cervical cancer in females in Korea for Strategies B and C and varying Pap screening coverages (using parameter values such that the control reproduction number, $\mathcal {R}_{vs}$, is less than one, as given in Table 10, so that the disease-free equilibrium of the model is locally-asymptotically stable, by Theorem 2.3). Parameter values used in these simulations are as given in Table 9, but with the following changes: (i) Strategy B: $q_f=0.95$, $\xi _f=0.06$ per year, (ii) Strategy C: $q_f=q_m=0.8$. In all simulations for the 90% Pap screening coverage, the following values were used: $d_{u3}g_f=0.27,~ h_{u3}y_f=q_{u3}z_f=w_f=0.35$ per year

Control strategy	Strategy B		Strategy C	
Pap screening coverage	$61\%$(baseline)	$90\%$	$61\%$(baseline)	$90\%$
Time of cervical cancer elimination (year)	2088	2085	2097	2094

### Spillover effect of vaccination-only strategy

In this section, the model (2.5) will be simulated for the case where vaccination is the only anti-HPV intervention implemented in Korea (i.e., no Pap screening), with the aim of quantifying and evaluating the potential spillover effect of vaccinating individuals of one gender on reducing the burden of HPV and related cancers in the other gender. Specifically, the following hypothetical scenarios of the vaccination-only strategy are considered:

(i) *Girls-only vaccination strategy:* This entails the annual vaccination of 99% of girls in the 12-17 year age group for the period between 2021 and 2060, and no vaccination of females (of age 18-26), boys, or males.
(ii) *Boys-only vaccination strategy:* This involves the annual vaccination of 99% of boys in the 12-17 year age group, and no vaccination of males (of age 18-26), girls, or females.

**Fig. 11:**
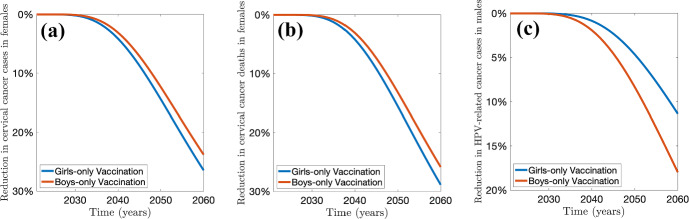
Simulation of the model (2.5) to assess the spillover benefit of vaccinating adolescents of one gender on the burden of HPV-related cancers in the other gender, in comparison to the worst-case scenario (with no vaccination or screening). The panels depict reduced proportion of cumulative (a) cervical cancer cases in females, (b) cervical cancer-induced deaths in females, and (c) HPV-related cancer cases in males, by 2060. Parameter values used in these simulations are as given in Table 9, but with the following changes: (i) Girls-only vaccination: $q_f=0.99$, $q_m=0$, (ii) Boys-only vaccination: $q_m=0.99$, $q_f=0$. In all simulations, the following values are used: $\xi _f=\xi _m=0$ per year (no catch-up vaccination), $d_{u3}g_f=h_{u3}y_f=q_{u3}z_f=w_f=0$ per year (no routine Pap screening).

**Fig. 12:**
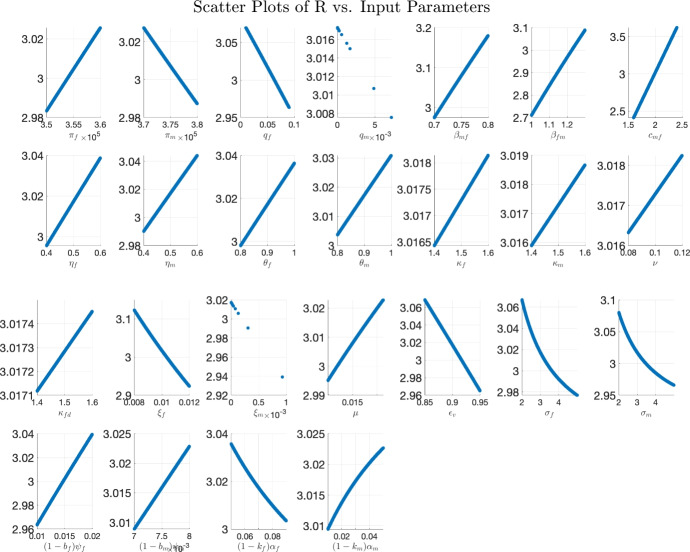
Scatter plots of the control reproduction number, $\mathcal {R}_{vs}$, of the model (2.5), with respect to each of the parameters in $\mathcal {R}_{vs}$ included in the sensitivity analysis. For each plot, the other parameter values used are as given in Table 9.

**Fig. 13:**
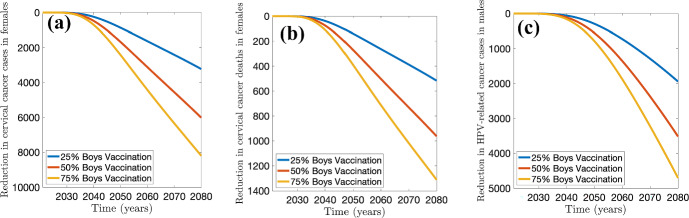
Simulation of the model (2.5) to assess the combined impact of Pap screening and vaccination (of girls, females, and boys) on the burden of HPV-related cancers in females and males in Korea by the year 2080, for three vaccination coverages for boys in addition to Strategy A, in comparison to Strategy A under baseline Pap screening coverage. The panels depict reduction in the cumulative (a) cervical cancer cases in females, (b) cervical cancer-induced deaths in females, and (c) HPV-related cancer cases in males, by 2080. Parameter values used in these simulations are as given in Table 9, but with the following changes: (i) Strategy A: $q_f=0.8$, (ii) 25% boys vaccination: $q_f=0.8$, $q_m=0.25$, (iii) 50% boys vaccination: $q_f=0.8$, $q_m=0.5$, (iv) 75% boys vaccination: $q_f=0.8$, $q_m=0.75$.

**Fig. 14:**
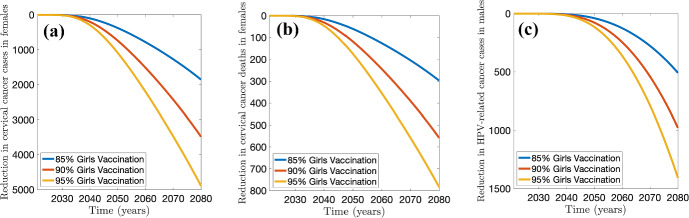
Simulation of the model (2.5) to assess the combined impact of Pap screening and vaccination (of girls and females) on the burden of HPV-related cancers in females and males in Korea by the year 2080, for three vaccination coverages for girls in addition to Strategy A, in comparison to Strategy A under baseline Pap screening coverage. The panels depict reduction in the cumulative (a) cervical cancer cases in females, (b) cervical cancer-induced deaths in females, and (c) HPV-related cancer cases in males, by 2080. Parameter values used in these simulations are as given in Table 9, but with the following changes: (i) Strategy A: $q_f=0.8$, (ii) 85% girls vaccination: $q_f=0.85$, (iii) 90% girls vaccination: $q_f=0.9$, (iv) 95% girls vaccination: $q_f=0.95$.

The model is simulated, using the baseline values of the parameters in Table 9 and in the caption of Figure 11, for the period from 2021 to 2060. The simulation results obtained are then compared with those generated under the worst-case scenario (when neither vaccination nor Pap screening is implemented).

The simulation results obtained, depicted in Figure 11, show that vaccinating boys only or girls only reduces cumulative cervical cancer cases in females, in comparison to the worst-case scenario without vaccination or screening. In particular, boys-only vaccination causes 24% reduction in cervical cancer cases by the year 2060 compared to the worst-case scenario, clearly showing a significant spillover benefit in vaccinating boys in reducing cumulative number of cervical cancer cases in females in Korea. Similarly, girls-only vaccination causes about 26% reduction in cumulative number of cervical cancer cases by the year 2060 in comparison to the worst-case scenario (see blue curve of Figure 11 (a)), which, as expected, is slightly higher than the spillover reduction generated by the boys-only vaccination (red curve in Figure 11 (a)). A similar trend is observed with respect to reductions in cumulative mortality due to cervical cancer in females (Figure 11(b)), where boys-only vaccination reduces 26% of cumulative cervical cancer-induced deaths in females by 2060, in comparison to the worst-case scenario (which is quite close to the 29% cumulative reduction obtained by vaccinating girls only at 99% coverage, in comparison to the worst-case). Finally, boys-only vaccination is, as expected, more effective in reducing the cumulative number of HPV-related cancer cases in males, than the spillover related to the implementation of girls-only vaccination (Figure 11(c)). In particular, while boys-only vaccination causes an 18% reduction of cumulative HPV-related cancers in males by the year 2060, in comparison to the baseline (where there is no vaccination or screening), girls-only vaccination only causes 11% reduction of these male cancers by the same time period, in comparison to the baseline (compare blue and red curves in Figure 11(c)). It is worth noting that the spillover effect of vaccinating boys in reducing cervical cancer cases and mortality in females is larger than that of vaccinating girls in reducing HPV-related cancer cases in males (compare subplots (a) and (b) with subplot (c) in Figure 11). This may be due to the fact that HPV-infected females are more likely to develop cancer, compared to HPV-infected males (Giuliano et al. 2008a; National Cancer Center Korea Central Cancer Registry 2022; Moscicki et al. 2004, 1998; Van Doornum et al. 1994).

## Discussion and Conclusion

HPV (Human *Papillomavirus*) accounts for virtually all cervical cancer cases and significantly contributes to anal, penile, vaginal, and head and neck cancers (de Martel et al. 2017). Although cervical cancer is one of the few human cancers that is vaccine-preventable and curable if detected early and treated, it continues to pose major health burden globally (causing an average of over 300,000 deaths annually (Bray et al. 2024; Schiffman et al. 2007)). It is the third most common female cancer in the Republic of Korea in the 15-44 year age group (causing an average of over 1,000 deaths per year (Bruni et al. 2023)). In order to curtail or mitigate the burden of cervical cancer (and other HPV-related cancers in females and males), Korea started the National Immunization Program (NIP) for HPV in 2016. This program provides routine HPV immunization to girls in the 12-17 year age group (Kwak and Hwang 2024). Korea also has the National Cancer Screening Program (NCSP), which offers free Pap screening to females over age 30 every 2 years since 2002, and to females over age 20 since 2016 (Shin et al. 2022). The purpose of the current study was to use mathematical modeling approaches, together with data analytics and numerical simulations, to assess the population-level impact of the aforementioned vaccination and Pap screening programs on the transmission dynamics of HPV and its related cancers in South Korea. A sex-structured mathematical model for the heterosexual transmission of HPV in Korea, in the presence of HPV vaccination and Pap screening, was formulated. Although only females are vaccinated in Korea under the current NIP, the model developed in this study allows for the vaccination of males (particularly boys of age group 12-17 years). This allows for the hypothetical assessment of the potential impact of male vaccination on reducing the burden of HPV and related cancers in Korea.

The disease-free equilibrium of this model was shown to be locally-asymptotically stable when a certain epidemiological threshold, known as the *control reproduction number*, is less than one. The epidemiological implication of this result is that the combined vaccination and Pap screening program implemented in South Korea could lead to the elimination of HPV and related cancers if the control reproduction number could be brought to (and maintained at) a value less than one, provided that the initial number of HPV-infected individuals is small enough. Numerical simulations of the model showed that vaccine-derived herd immunity cannot be achieved in Korea under the current NIP coverage (which essentially amounts to 88% of females vaccinated at steady-state). The herd immunity can be achieved if, additionally, at least 65% of males are vaccinated at steady-state (if males are not vaccinated, which is the current situation under the NIP, then at least 99% of females need to be vaccinated to achieve herd immunity). Further rigorous analyses showed that the special case of the model with negligible disease-induced mortality could have one or three endemic equilibria when the associated reproduction number exceeds one. Using a Krasnoselskii sub-linearity argument, it was shown that, for this special case and with vaccine efficacy assumed to be perfect, the unique endemic equilibrium of the model is locally-asymptotically stable whenever its associated reproduction number exceeds one. The implication of this result is that vaccination and Pap screening, at these coverage and efficacy levels, are inadequate to lead to the elimination of HPV and related cancers in Korea (since, in this case, HPV and related cancers will persist in the population).

In order to estimate some of the unknown parameters of the model, a simplified version of the model without Pap screening and with the three *cervical intraepithelial neoplasia* (CIN) and *intraepithelial neoplasia in males* (INM) stages lumped into one epidemiological compartment, was calibrated using observed data for cumulative cases and mortality for HPV-related cancers in Korea for the period from 1999 to 2020 (National Cancer Center Korea Central Cancer Registry 2022). The disease-free equilibrium of the model is shown to be locally-asymptotically stable when the reproduction number of the model is less than unity. The epidemiological implication of this result is that the interventions implemented (i.e., vaccination and Pap screening) can lead to the effective control and elimination of HPV when the reproduction number of the model is less than one provided the initial number of infected individuals is small enough (i.e., fall within the basin of attraction of the disease-free equilibrium). The local asymptotic stability was extended to the global asymptotic stability of the disease-free equilibrium for a special case when the disease-induced mortality and the coverage for catch-up vaccination (for both females and males) in Korea are negligible, when its associated reproduction number is less than one. Epidemiologically-speaking, this result show that HPV (and, hence, HPV-related cancers) will be eliminated in Korea if the associated control reproduction number can be brought down to (and maintained at) a value less than one, regardless of the initial size of the number of HPV-infected individuals in the community.

Using the estimated values of the unknown parameters obtained from this calibration, the full model was then used to conduct global parameter sensitivity analysis to determine the parameters of the model that have the highest impact on a chosen response function, namely the control reproduction number $\mathcal {R}_{vs}$. The results of the sensitivity analysis revealed that parameters related to the transmission of HPV, recovery from symptomatic HPV infection, and HPV vaccine coverage and efficacy were the most influential in determining the value of the control reproduction number (hence, the burden of HPV and related cancers in Korea). The implication of these results are that effective control and mitigation strategies could be formulated by targeting these identified parameters.

The full model was simulated to assess the population-level impact of the combined vaccination and Pap screening strategies currently being implemented in Korea (defined as Strategy A), and to evaluate the potential impact of two hypothetical extended versions of these strategies (namely Strategies B and C). These strategies are formally defined below:

- Strategy A: This is the post-NIP vaccination strategy that entails the annual vaccination of 80% of girls (12-17 years of age) and annual catch-up vaccination of 30,000 females (18-26 years of age) with baseline Pap screening (of 61% of adult females in the age group 20-74 years of age).
- Strategy B: This contains Strategy A together with the increase in the annual vaccination coverage for girls from 80% to 95% and for females from 30,000 to 175,000.
- Strategy C: This contains Strategy A together with the annual vaccination of 80% of boys (12-17 years of age).

The simulation results obtained showed that while all three strategies will lead to a significant reduction in the disease burden (measured in terms of yearly cases and mortality of HPV-related cancers), only Strategies B and C will lead to the elimination of HPV and related cancers in Korea. This is because only the latter two strategies will result in bringing the associated control reproduction number of the model (denoted by $\mathcal {R}_{vs}$) below one. In particular, it was shown that, based on the parameter values used in our simulations, that cervical cancer can be eliminated in Korea using Strategy B in about 60 years (Strategy C could achieve elimination in about 70 years). These simulations suggest that increasing the current levels of the vaccination and Pap screening coverages in Korea, as well as extending the vaccination coverage to adult females and males (to specifically attain the levels in Strategies B and C) could lead to a dramatic reduction (and even elimination) of the burden of HPV and related cancers in Korea.

The model was further simulated to assess the potential impact of vaccinating one gender (only) on reducing the burden of HPV and related cancers in the other gender for the hypothetical case where Pap screening is not implemented. In other words, the model was used to explore and quantify the potential *spillover* benefit of vaccination of one gender on the other (when Pap screening is not implemented). These simulation results obtained, which are based on vaccinating 99% of one gender only, showed that vaccinating 99% of boys only induced a significant spillover benefit in reducing the cumulative number of cervical cancer cases and mortality in females in Korea, in comparison to the worst-case scenario where no vaccination or Pap screening is implemented. This reduction was larger than the spillover benefit achieved (in reducing the disease burden in males) by vaccinating 99% of girls only. Thus, this study showed that extending the current post-NIP strategy in Korea to also include the vaccination of boys (and catch-up vaccination of adult females) will significantly reduce the burden of HPV and related cancers in both females and males in Korea.

Some of the limitation of the model developed in this study include not accounting for homosexual transmission of HPV, reinfection of HPV and waning of infection-acquired and vaccine-acquired immunity against HPV. Adding the dynamics of MSM (men who have sex with men) into the model might affect the level or size of some of the epidemiological thresholds presented in this study. The authors hope to address these limitations in a future study. It should, however, be mentioned that the modeling framework presented in this study, for the dynamics of HPV and related cancers in Korea, can be adapted and applied to other countries or regions or jurisdictions interested in assessing the population-level impact of vaccination and Pap screening on reducing the burden of HPV and related cancers, by adjusting demographic parameters, the vaccination and Pap screening parameters, and the parameter for number of sexual contacts pertinent to the area. Overall, this study showed that the prospect of the effective control or elimination of HPV and related cancers in Korea, using the combined vaccination and Pap screening program, is promising, provided that the coverage levels are high enough and the program is extended to include the vaccination of boys and adult females (with high enough coverage).

## Proof of Lemma 2.1

### Proof

Consider the model (2.5) with $Q_{2u}(0)$, $Q_{2d}(0)$, $J_{2m}(0) >0$, and all other state variables being non-negative at time $t=0$. The equation for the rate of change of the compartment for unvaccinated susceptible females ($S_f(t)$) can be rewritten as:

$$\begin{aligned} &  \frac{d}{dt} \left[ S_f(t)\exp \left( (\xi _f+\mu ) t + \int _0^t \lambda _{m_af} (s)\; ds\right) \right] \\ &  \quad = (1-q_f)\pi _f \exp \left( (\xi _f+\mu ) t + \int _0^t \lambda _{m_af}(s)\; ds\right) ,\end{aligned}$$

and integrating both sides with respect to *t* gives,

$$\begin{aligned}&S_f(t) \exp \left( (\xi _f+\mu ) t + \int _0^{t} \lambda _{m_af} (s) \; ds\right) \\&= S_f(0) + (1-q_f) \pi _f\int _0^{t} \exp \left( (\xi _f+\mu ) t + \int _0^t \lambda _{m_af} (s) \; ds\right) \; dt, \end{aligned}$$

from which it follows that (noting that $0\le q_f\le 1$),

$$\begin{aligned} S_f(t)&= \exp \left( - (\xi _f+\mu ) t -\int _0^t \lambda _{m_af} (s) \; ds\right) \\&\bigg [S_f(0) + (1-q_f) \pi _f\int _0^{t} \left. \exp \left( \int _0^t (\xi _f+\mu ) t +\lambda _{m_af} (s) \; ds \right) \; dt \right] \\&\ge 0,\,\,\mathrm{for\,\,all}\,\,t>0. \end{aligned}$$

Next, given the non-negativity of $S_f(t)$, the equation for the rate of change of the vaccinated female population ($V_f(t)$) satisfies the following:

$$\begin{aligned} \frac{d {V}_f}{dt} = q_f \pi _f + \xi _f S_f- (1-\varepsilon _v) \lambda _{m_af} V_f - \mu V_f \ge q_f \pi _f - (1-\varepsilon _v) \lambda _{m_af} V_f - \mu V_f, \end{aligned}$$

which can be rewritten as,

$$\begin{aligned} &  \frac{d}{dt} \left[ V_f(t)\exp \left( \mu t +\int _0^t (1-\varepsilon _v)\lambda _{m_af} (s) \; ds \right) \right] \\ &  \ge q_f\pi _f \exp \left( \mu t +\int _0^t (1-\varepsilon _v)\lambda _{m_af} (s) \; ds \right) ,\end{aligned}$$

from which it follows (by integrating both sides) that,

$$\begin{aligned}&V_f(t) \exp \left( \mu t +\int _0^t (1-\varepsilon _v)\lambda _{m_af} (s) \; ds \right) \\&\ge V_f(0) + q_f \pi _f\int _0^{t} \exp \left( \mu t +\int _0^t (1-\varepsilon _v)\lambda _{m_af} (s) \; ds \right) \; dt. \end{aligned}$$

Hence, $V_f(t)\ge 0$ for all $t >0$. Similarly, using this approach, it can be shown that the state variables $E_f(t), I_f(t), P_f(t), S_m(t), V_m(t), E_m(t), I_m(t)$ and $P_m(t)$ of the model (2.5) are also non-negative for all $t >0$. The next step is to prove the non-negativity of the state variables $Q_{iu}, Q_{id}, J_{im} (i = 1,2,3)$ of the model (2.5). This is done by contradiction as follows. First of all, the equations for the rate of change of $Q_{iu} (i = 1,2,3)$ can be rewritten as:

A.1 $$\begin{aligned} \frac{d}{dt}\left[ Q_{1u} \exp \left( (g_f + \mu )t \right) \right]&= [(1-k_f)\alpha _f P_f + h_{u2}y_fQ_{2u}]\exp ((g_f+\mu )t), \nonumber \\ \frac{d}{dt}\left[ Q_{2u} \exp \left( (y_f + \mu )t \right) \right]&= [(1-d_{u1}-d_{u3})g_f Q_{1u} + q_{u2}z_fQ_{3u}]\exp ((y_f+\mu )t), \\ \frac{d}{dt}\left[ Q_{3u} \exp \left( (z_f + \mu )t \right) \right]&= [(1-h_{u1}-h_{u2}-h_{u3})y_f Q_{2u}]\exp ((z_f+\mu )t), \nonumber \end{aligned}$$

which, by integrating both sides with respect to *t*, gives

A.2 $$\begin{aligned} Q_{1u}(t)&= Q_{1u}(0) + \left[ \int _0^t \{(1-k_f)\alpha _f P_{f}(s) + h_{u2}y_fQ_{2u}(s)\} \nonumber \right. \\&\quad \left. \exp ((g_f+\mu )s) \; ds\right] \exp ((-g_f-\mu )t), \nonumber \\ Q_{2u}(t)&= Q_{2u}(0) + \left[ \int _0^t \{(1-d_{u1}-d_{u3})g_f Q_{1u}(s)\nonumber \right. \\&\quad \left. + q_{u2}z_fQ_{3u}(s)\} \exp ((y_f+\mu )s) \; ds \right] \exp ((-y_f-\mu )t), \nonumber \\ Q_{3u}(t)&= Q_{3u}(0) + \left[ (1-h_{u1}-h_{u2}-h_{u3})y_f \int _0^t Q_{2u}(s) \nonumber \right. \\&\quad \left. \exp ((z_f+\mu )s) \; ds\right] \exp ((-z_f-\mu )t). \end{aligned}$$

Suppose, now, that $Q_{2u}(t) \le 0$ for some *t* in $(0, \infty ).$ Since $Q_{2u}(0) > 0$ (by assumption), there exists a $t_0 > 0$ such that $t_0 = \inf \{t >0 : Q_{2u}(t) = 0\}.$ Note that $P_f(t) \ge 0$ for all $t \ge 0$ from the proof above. Furthermore, all the model parameters, as well as the proportions $1-k_f, 1-d_{u1}-d_{u3}, 1-h_{u1}-h_{u2}-h_{u3}$, are non-negative (as defined in Section 2). Hence, it follows from (A.2) and the definition of $t_0,$ that both $Q_{1u}(t)$ and $Q_{3u}(t)$ are non-negative for any $t \in [0,t_0].$ Furthermore, it can be seen by evaluating the second equation of (A.2) at $t=t_0$, that

$$\begin{aligned} Q_{2u}(t_0)=&\; Q_{2u}(0) +\left[ \int _0^{t_0} \{(1-d_{u1}-d_{u3})g_f Q_{1u}(s) + q_{u2}z_fQ_{3u}(s)\} \exp ((y_f+\mu )s) \; ds \right] \\&\exp ((-y_f-\mu )t_0). \end{aligned}$$

Using the non-negativity of $Q_{1u}(t)$ and $Q_{3u}(t)$ on $[0,t_0],$ and the strict positivity of $Q_{2u}(0)$, the above expression for $Q_{2u}(t_0)$ is strictly positive. This contradicts the definition of $t_0$ (which requires $Q_{2u}(t_0) = 0$). Thus, $Q_{2u}(t) > 0$ for all $t \ge 0.$ Using the fact that $Q_{2u}(t)$ is strictly positive for all $t \ge 0$, it can be seen from the first and third equations of (A.2), that $Q_{1u}(t)$ and $Q_{3u}(t)$ are also strictly positive for all $t > 0.$ Similarly, the non-negativity of $Q_{id}(t)$, with $i = 1,2,3$, can be proved, using the fact that $Q_{1u}(t), Q_{2u}(t)$ and $Q_{3u}(t)$ are all strictly positive for $t>0$. Furthermore, the proof by contradiction can also be used to show that $J_{im}(t)$, with $i = 1,2,3$, are strictly positive for all $t>0$. Finally, the non-negativity of the remaining state variables, $C_u(t), C_d(t), R^C_f(t), R_f(t), C_m(t), R_m^C(t)$, and $R_m(t)$, follows from the non-negativity of the state variables established above. Hence, all non-negative initial conditions of the model (2.5), with $Q_{2u}(0), Q_{2d}(0), J_{2m}(0)>0$, remain non-negative for all $t>0$. $\square$

## Non-negativity of the Control Reproduction Number ($\mathcal {R}_{vs}$)

Recall from Section 2.2.1 that the control reproduction number of the model (2.5) is given by

$$\begin{aligned} \mathcal {R}_{vs}= \rho (F_sV_s^{-1}) = \sqrt{\mathcal {R}_f \mathcal {R}_m}, \end{aligned}$$

where (with all the associated parameters and quantities as defined in Section 2.2.1),

$$\begin{aligned} \mathcal {R}_f =&\frac{\beta _{fm}c_{mf}\mu p_m^*}{\pi _m} \left[ \frac{\eta _{f}}{k_1} + \frac{\sigma _f}{k_1k_2} + \frac{\theta _{f}\sigma _fl_1}{k_1k_2k_3} + \right. \\&+\left. \frac{\theta _{f}\sigma _fl_1l_2\{\iota _{f2} +\kappa _fl_3k_6 +\kappa _fl_3l_4\}}{k_1k_2k_3\iota _{f1}} + \frac{\theta _{f}\nu \sigma _fl_1l_2(j_{f1} +\kappa _{fd}j_{f2} +\kappa _{fd}j_{f3})}{k_1k_2k_3\iota _{f1}\iota _{f3}} \right] ,\\ \mathcal {R}_m =&\frac{\beta _{mf}c_{mf}\mu p_{f}^*}{\pi _m} \left[ \frac{\eta _m}{e_1} + \frac{\sigma _m}{e_1e_2} + \frac{\theta _m\sigma _m\psi _m(1-b_m)}{e_1e_2e_3} \right. \\&\left. + \frac{\theta _m\sigma _m \psi _m(1-b_m) \alpha _m (1-k_m)\{\iota _{m2} + \kappa _mg_m(1-d_m)e_6 + \kappa _mg_m(1-d_m)f_1\}}{e_1e_2e_3\iota _{m1}} \right] . \end{aligned}$$

Since the parameters of the model are all non-negative and the proportions related to some of the parameters are between zero and one, the non-negativity of $\mathcal {R}_f$ and $\mathcal {R}_m$ (hence that of $\mathcal {R}_{vs}$) depend on the non-negativity of the expressions for $\iota _{f1}, \iota _{f2}, \iota _{f3}, j_{f1}, j_{f2}, j_{f3}, \iota _{m1}$, and $\iota _{m2}$. In order to prove the non-negativity of $j_{f1}, j_{f2},$ and $j_{f3}$, it is convenient to rewrite their expressions by factoring out $g_f$ (which is automatically non-negative) and organizing the inner expressions in terms of $d_{u3}$ (for $j_{f1}$) and $\mu$ (for $j_{f2}$ and $j_{f3}$) in descending order (note that $d_{u3}$ and $\mu$ are non-negative), as follows:

$$\begin{aligned} j_{f1}&= g_f(\iota _{f4} d_{u3}+\iota _{f5}), \\ j_{f2}&= g_f(\iota _{f6}\mu ^3 + \iota _{f7}\mu ^2 + \iota _{f8}\mu +\iota _{f9}), \\ j_{f3}&= g_f(\iota _{f10}\mu ^2 + \iota _{f11}\mu + \iota _{f12}), \end{aligned}$$

where,

B.1 $$\begin{aligned} \iota _{f4} =&\{y_fz_f(1-q_{u_2}(1-h_{u_1}-h_{u_2}-h_{u_3})) + \mu (y_f+z_f) + \mu ^2\}\nonumber \\&\{\zeta _2\zeta _3(1-q_{d_2}(1-h_{d_1}-h_{d_2})) \nonumber \\&+\mu (\zeta _2 + \zeta _3) + \mu ^2\}, \nonumber \\ \iota _{f5} =&y_f\zeta _2 h_{d_2}h_{u_3}(1-d_{u_1}-d_{u_3})(z_f+\mu )(\zeta _3+\mu )\nonumber \\&+y_fz_f\zeta _2\zeta _3 q_{u_3}q_{d_2}h_{d_2}(1-d_{u_1}-d_{u_3})(1-h_{u_1}-h_{u_2}-h_{u_3}),\nonumber \\ \iota _{f6} =&d_{u_3}\zeta _1 (1-d_{d_1})+h_{u_3}y_f(1-d_{u_1}-d_{u_3}),\nonumber \\ \iota _{f7} =&d_{u_3}\zeta _1(1-d_{d_1})(y_f+\zeta _3+z_f)\nonumber \\&+(1-d_{u_1}-d_{u_3})h_{u_3}y_f(\zeta _1+\zeta _3+z_f),\nonumber \\ \iota _{f8} =&d_{u_3}\zeta _1 \{y_f\zeta _3+ y_fz_f(1-(1-h_{u_1}-h_{u_2}\nonumber \\&-h_{u_3})q_{u_2})+\zeta _3z_f\} (1-d_{d_1}) + (1-d_{u_1}-d_{u_3}) \nonumber \\&h_{u_3}y_f (\zeta _1\zeta _3+\zeta _1z_f+\zeta _3z_f) + (1-d_{u_1}\nonumber \\&-d_{u_3})(1-h_{u_1}-h_{u_2}-h_{u_3})q_{d_2}q_{u_3}y_f\zeta _3z_f,\nonumber \\ \iota _{f9} =&d_{u_3}y_f\zeta _1\zeta _3z_f(1-d_{d_1})(1-q_{u_2}) + h_{u_3}y_f\zeta _1\zeta _3z_f(1-d_{u_1}-d_{u_3})\nonumber \\&+ (1-d_{u_1}-d_{u_3}) \nonumber \\&(1-h_{u_1}-h_{u_2}-h_{u_3}) q_{d_2}q_{u_3}y_f\zeta _1\zeta _3z_f+(1-d_{d_1})\nonumber \\&(h_{u_1}+h_{u_2}+h_{u_3}) d_{u_3}q_{u_2}y_f\zeta _1\zeta _3z_f,\nonumber \\ \iota _{f10} =&d_{u_3}\zeta _1\zeta _2(1-h_{d_1}-h_{d_2})(1-d_{d_1})\nonumber \\&+h_{u_3}y_f\zeta _2(1-d_{u_1}-d_{u_3})(1-h_{d_1}-h_{d_2})\nonumber \\&+y_fz_fq_{u_3}(1-d_{u_1}-d_{u_3})(1-h_{u_1}-h_{u_2}-h_{u_3}),\nonumber \\ \iota _{f11} =&d_{u_3}\zeta _1 \zeta _2 (1-h_{d_1}-h_{d_2})(1-d_{d_1})(y_f+z_f)+h_{u_3}y_f\zeta _2(\zeta _1+z_f)\nonumber \\&(1-d_{u_1}-d_{u_3})(1-h_{d_1}-h_{d_2})\nonumber \\&+ q_{u_3}y_fz_f(\zeta _1+\zeta _2)(1-d_{u_1}-d_{u_3})(1-h_{u_1}-h_{u_2}-h_{u_3}), \nonumber \\ \iota _{f12} =&d_{u_3}\zeta _1\zeta _2y_fz_f(1-d_{d_1})(1-h_{d_1}-h_{d_2})\nonumber \\&(1-q_{u_2}(1-h_{u_1}-h_{u_2}-h_{u_3}))\nonumber \\&+ h_{u_3}y_f\zeta _1\zeta _2z_f(1-d_{u_1}-d_{u_3})(1-h_{d_1}-h_{d_2}) \nonumber \\&+ q_{u_3}y_fz_f\zeta _1\zeta _2(1-d_{u_1}-d_{u_3})(1-h_{u_1}-h_{u_2}-h_{u_3})(1-h_{d_2}(1-d_{d_1})). \end{aligned}$$

Then, it immediately follows from the non-negativity of the parameters of the model (and the proportions in equations (B.1) being less than one) that $\iota _{f4}, \iota _{f5}, \dots , \iota _{f12}$ are all non-negative, hence $j_{f1}, j_{f2},$ and $j_{f3}$ are non-negative. The non-negativity of the remaining terms, $\iota _{f1}, \iota _{f2}, \iota _{f3}, \iota _{m1}$, and $\iota _{m2}$, can be shown by rewriting their expressions as follows:

B.2 $$\begin{aligned} \iota _{f1}= &  \mu ^3 + \mu ^2(g_f+y_f+z_f) + \mu \{g_fy_f(1-h_{u2}(1-d_{u1}-d_{u3})) \nonumber \\ &  +y_fz_f(1-q_{u2}(1-h_{u1}-h_{u2}-h_{u3})) \nonumber \\  &  + g_fz_f\} + g_fy_fz_f\{(1-q_{u2})(1-h_{u2})\nonumber \\ &  +h_{u2}(d_{u1}+d_{u3})+q_{u2}(h_{u1}+h_{u3})\}, \nonumber \\ \iota _{f2}= &  (1-q_{u2}(1-h_{u_1}-h_{u_2}-h_{u_3}))y_fz_f + \mu (y_f + z_f + \mu ), \nonumber \\ \iota _{f3}= &  \mu ^3 + \mu ^2(\zeta _1 +\zeta _2 +\zeta _3) + \mu \{ \zeta _1 \zeta _2(1-h_{d_2} + d_{d_1} h_{d_2}) \nonumber \\ &  + \zeta _2\zeta _3(1-q_{d_2}(1-h_{d_1}-h_{d_2})) + \zeta _1\zeta _3\} \nonumber \\ &  + \zeta _1\zeta _2\zeta _3\{(1-h_{d_2})(1-q_{d_2})+q_{d_2}h_{d_1}+d_{d_1}h_{d_2} \}, \nonumber \\ \iota _{m1}= &  \mu ^3 + \mu ^2(g_m+y_m+z_m) + \mu \{g_my_m(1-h_{m2}(1-d_m)) \nonumber \\ &  +y_mz_m(1-q_{m2}(1-h_{m1}-h_{m2})) \nonumber \\  &  + g_mz_m\}+ g_my_mz_m\{(1-q_{m2})(1-h_{m2})\nonumber \\ &  +h_{m2}d_m+q_{m2}h_{m1}\}, \nonumber \\ \iota _{m2}= &  \mu ^2 + \mu (y_m+z_m) +y_mz_m(1-q_{m2}(1-h_{a_1}-h_{a_2})). \end{aligned}$$

Thus, since all the parameters of the model are non-negative and the proportions in equations (B.2) are less than or equal to one, it follows from (B.2) that $\iota _{f1}, \iota _{f2}, \iota _{f3}, \iota _{m1},$ and $\iota _{m2}$ are all non-negative. Hence, the control reproduction number, $\mathcal {R}_{vs}$, of the model (2.5) is non-negative.

## Coefficients of the Polynomial (2.20)

The expressions for the coefficients of the quartic (2.20), given in in Section 2.2.2, are given by:

$$\begin{aligned} a_4 =&(\mu (1-\varepsilon _vq_f) + \xi _f(1-\varepsilon _v)) \pi _f(1-\varepsilon _v)^2\{\mathcal {R}_{f}\pi _f(\xi _m+\mu )\mu +\pi _m(\mu (1-\varepsilon _v q_m) + \xi _m(1-\varepsilon _v))\}\\&\{(1-\varepsilon _v)\mathcal {R}_{f}\pi _f(\xi _m+\mu )\mu +\pi _m(\mu (1-\varepsilon _v q_m) + \xi _m(1-\varepsilon _v)) \mu \} \quad \ge 0, \\ a_3 =&(\mu (1-\varepsilon _v q_f) + \xi _f(1-\varepsilon _v))\pi _f(1-\varepsilon _v)(\xi _m+\mu )[2(1-\varepsilon _v)\\&\mathcal {R}_f^2\pi _f^2(\xi _m+\mu )\mu ^2(\mu (1-\varepsilon _vq_f) + \xi _f(1-\varepsilon _v))\\&+ \mathcal {R}_f\pi _f\pi _m\mu (\mu (1-\varepsilon _vq_f) + \xi _f(1-\varepsilon _v))(\mu (1-\varepsilon _v q_m) + \xi _m(1-\varepsilon _v))((1-\varepsilon _v)(\xi _m+\mu )\\&+\mu ) + 2\pi _m^2\mu (\mu (1-\varepsilon _v q_m) + \xi _m(1-\varepsilon _v))^2((1-\varepsilon _v)(\xi _f+\mu )+\mu ) + \mathcal {R}_f\pi _f\pi _m\mu \\&(\mu (1-\varepsilon _v q_m) + \xi _m(1-\varepsilon _v))((1-\varepsilon _v)(\xi _f+\mu )+\mu ) ((1-\varepsilon _v)(\xi _m+\mu )+\mu )]- \mathcal {R}_f\mathcal {R}_m\pi _f\pi _m \\&(1-\varepsilon _v)^2(\xi _f+\mu )(\xi _m+\mu )\mu ^2 \{\mathcal {R}_f\pi _f(1-\varepsilon _v)(\xi _m+\mu )\mu \\&+\pi _m(\mu (1-\varepsilon _v q_m) + \xi _m(1-\varepsilon _v))^2\} \\ \ge&(\mu (1-\varepsilon _v q_f) + \xi _f(1-\varepsilon _v))\pi _f(1-\varepsilon _v)(\xi _m+\mu )[\pi _m^2\mu (\mu (1-\varepsilon _v q_m) \\&+ \xi _m(1-\varepsilon _v))^2((1-\varepsilon _v)(\xi _f+\mu )+\mu )\\ &+ \mathcal {R}_f\pi _f\pi _m\mu (\mu (1-\varepsilon _v q_m) + \xi _m(1-\varepsilon _v))((1-\varepsilon _v)(\xi _f+\mu )+\mu ) ((1-\varepsilon _v)(\xi _m+\mu )+\mu )] \\&- \mathcal {R}_{vs}\pi _f\pi _m (1-\varepsilon _v)^2(\xi _f+\mu )(\xi _m+\mu )\mu ^2 \{\mathcal {R}_f\pi _f(1-\varepsilon _v)(\xi _m+\mu )\mu \\&+\pi _m(\mu (1-\varepsilon _v q_m) + \xi _m(1-\varepsilon _v))^2\} \\ =&(\mu (1-\varepsilon _v q_m) + \xi _m(1-\varepsilon _v))^2\pi _f\pi _m^2(1-\varepsilon _v)(\xi _m+\mu )\mu \{(\mu (1-\varepsilon _v q_f)\\&+ \xi _f(1-\varepsilon _v))((1-\varepsilon _v)(\xi _f+\mu )+\mu )\\&-\mathcal {R}_{vs}(1-\varepsilon _v)(\xi _f+\mu )\mu \} + \mathcal {R}_f\pi _f^2\pi _m(1-\varepsilon _v)(\xi _m+\mu )\mu \{(\mu (1-\varepsilon _vq_f) \\&+ \xi _f(1-\varepsilon _v))(\mu (1-\varepsilon _v q_m)\\&+ \xi _m(1-\varepsilon _v))((1-\varepsilon _v)(\xi _f+\mu )+\mu ) ((1-\varepsilon _v)(\xi _m+\mu )+\mu )\\&-\mathcal {R}_{vs}(1-\varepsilon _v)^2(\xi _f+\mu )(\xi _m+\mu )\mu ^2\} \\ \ge&(1-\mathcal {R}_{vs})\{(\mu (1-\varepsilon _v q_m) + \xi _m(1-\varepsilon _v))^2\pi _f\pi _m^2(1-\varepsilon _v)(\xi _m+\mu )\mu (1-\varepsilon _v)^2(\xi _f+\mu )(\xi _m+\mu )\mu ^2 \\&+\mathcal {R}_f\pi _f^2\pi _m(1-\varepsilon _v)(\xi _m+\mu )\mu (1-\varepsilon _v)^2(\xi _f+\mu )(\xi _m+\mu )\mu ^2\} \quad \ge 0 \;\bigg ( \;\text{ if }\; \mathcal {R}_{vs}\le 1 \;\bigg ),\\ a_2 =&(\mu (1-\varepsilon _vq_f) + \xi _f(1-\varepsilon _v))\pi _f (\xi _m\!+\!\mu )[(1\!-\!\varepsilon _v)\mathcal {R}_{f}^2\pi _f^2(\xi _m\!+\!\mu )\mu ^2(\mu (1\!-\!\varepsilon _vq_f) \!+\! \xi _f(1-\varepsilon _v))^2 \\&+ \mathcal {R}_f\pi _f\pi _m\mu (\mu (1-\varepsilon _vq_f) + \xi _f(1-\varepsilon _v))(\mu (1-\varepsilon _v q_m) + \xi _m(1-\varepsilon _v)) ((1-\varepsilon _v)(\xi _m+\mu )+\mu )\\&((1-\varepsilon _v)(\xi _f+\mu )+\mu ) + \pi _m^2\mu (\mu (1-\varepsilon _v q_m) + \xi _m(1-\varepsilon _v))^2 ((1-\varepsilon _v)(\xi _f+\mu )+\mu )^2 \\&+ \pi _m (\mu (1-\varepsilon _v q_m) + \xi _m(1-\varepsilon _v))(\xi _f+\mu )\mu ^2 \{\mathcal {R}_f\pi _f(1-\varepsilon _v)\\&((1-\varepsilon _v)(\xi _m+\mu )+\mu )+ 2\pi _m(1-\varepsilon _v) \\&(\mu (1-\varepsilon _v q_m) + \xi _m(1-\varepsilon _v))\}] -\mathcal {R}_f\mathcal {R}_m\pi _f\pi _m(1-\varepsilon _v)(\xi _f+\mu )(\xi _m+\mu )\mu ^2\\&\{2\mathcal {R}_f\pi _f(1-\varepsilon _v) (\xi _m+\mu )\mu \\&(\mu (1-\varepsilon _v q_f) + \xi _f(1-\varepsilon _v)) + \pi _m(\mu (1-\varepsilon _v q_f) + \xi _f(1-\varepsilon _v))(\mu (1-\varepsilon _v q_m) + \xi _m(1-\varepsilon _v))^2\\&+ \pi _m(\mu (1-\varepsilon _v q_m) + \xi _m(1-\varepsilon _v))^2 ((1-\varepsilon _v)(\xi _f+\mu )+\mu )\}\\ \ge&\mathcal {R}_f (\mu (1-\varepsilon _v q_f) + \xi _f(1-\varepsilon _v))\pi _f^2\pi _m\mu \{(\mu (1-\varepsilon _v q_m) \\ &+ \xi _m(1-\varepsilon _v))(\xi _f+\mu )(1-\varepsilon _v)\mu ((1-\varepsilon _v) \\&(\xi _m+\mu )+\mu )+(\mu (1-\varepsilon _v q_f) + \xi _f(1-\varepsilon _v))(\mu (1-\varepsilon _v q_m) + \xi _m(1-\varepsilon _v))((1-\varepsilon _v)(\xi _f+\mu )+\mu ) \\&((1-\varepsilon _v)(\xi _m+\mu )+\mu ) -2\mathcal {R}_{vs}(1-\varepsilon _v)^2(\xi _f+\mu )(\xi _m+\mu )\mu ^2\} + (\mu (1-\varepsilon _v q_m)\\&+ \xi _m(1-\varepsilon _v))^2\pi _f\pi _m^2 \\&(\xi _m+\mu )\{(\mu (1-\varepsilon _vq_f) + \xi _f(1-\varepsilon _v))\mu ((1-\varepsilon _v)(\xi _f+\mu )+\mu )^2-\mathcal {R}_{vs}(1-\varepsilon _v)(\xi _f+\mu ) \\&((1-\varepsilon _v)(\xi _f+\mu )+\mu )\mu ^2 + (\mu (1-\varepsilon _vq_f) + \xi _f(1-\varepsilon _v))(1-\varepsilon _v)(\xi _f+\mu )\mu ^2(2-\mathcal {R}_{vs})\}\\ \ge&(1-\mathcal {R}_{vs})[\mathcal {R}_f (\mu (1-\varepsilon _v q_f) + \xi _f(1-\varepsilon _v))\pi _f^2\pi _m\mu (1-\varepsilon _v)^2(\xi _f+\mu )(\xi _m+\mu )\mu ^2\\&+ (\mu (1-\varepsilon _v q_m) \\&+ \xi _m(1-\varepsilon _v))^2\pi _f\pi _m^2(\xi _m+\mu )\{(1-\varepsilon _v)(\xi _f+\mu )((1-\varepsilon _v)(\xi _f+\mu )+\mu )\mu ^2 + (\mu (1-\varepsilon _vq_f) \\&+ \xi _f(1-\varepsilon _v))(1-\varepsilon _v)(\xi _f+\mu )\mu ^2\}] \quad \ge 0 \;\bigg ( \;\text{ if }\; \mathcal {R}_{vs}\le 1 \;\bigg ),\\ a_1 =&(\mu (1-\varepsilon _vq_f) + \xi _f(1-\varepsilon _v))(\mu (1-\varepsilon _v q_m) + \xi _m(1-\varepsilon _v))\pi _f\pi _m(\xi _f+\mu )(\xi _m+\mu )\mu ^2 [\mathcal {R}_f\pi _f \\&(\mu (1-\varepsilon _v q_f) + \xi _f(1-\varepsilon _v))((1-\varepsilon _v)(\xi _m+\mu )+\mu ) + 2\pi _m(\mu (1-\varepsilon _v q_m) + \xi _m(1-\varepsilon _v))((1-\varepsilon _v) \\&(\xi _f+\mu )+\mu )]-\mathcal {R}_f\mathcal {R}_m\pi _f\pi _m (\xi _f+\mu )(\xi _m+\mu )\mu ^2\\&\{\pi _m(1-\varepsilon _v)(\xi _f+\mu )\mu (\mu (1-\varepsilon _v q_m) + \xi _m(1-\varepsilon _v))^2 \\&+ \mathcal {R}_f\pi _f(1-\varepsilon _v)(\xi _m+\mu )\mu (\mu (1-\varepsilon _vq_f) + \xi _f(1-\varepsilon _v))^2 + \pi _m(\mu (1-\varepsilon _vq_f) + \xi _f(1-\varepsilon _v)) \\&(\mu (1-\varepsilon _v q_m) + \xi _m(1-\varepsilon _v))^2((1-\varepsilon _v)(\xi _f+\mu )+\mu )\}\\ \ge&(\mu (1-\varepsilon _v q_m) + \xi _m(1-\varepsilon _v))^2 \pi _f\pi _m^2(\xi _f+\mu )(\xi _m+\mu )\mu ^2\{2(\mu (1-\varepsilon _v q_f)\\&+ \xi _f(1-\varepsilon _v))((1-\varepsilon _v)(\xi _f+\mu )\\&+\mu )-\mathcal {R}_{vs}(1-\varepsilon _v)(\xi _f+\mu )\mu -\mathcal {R}_{vs}(\mu (1-\varepsilon _v q_f) + \xi _f(1-\varepsilon _v))((1-\varepsilon _v)(\xi _f+\mu )+\mu ))\} \\&+ \mathcal {R}_f(\mu (1-\varepsilon _v q_f) + \xi _f(1-\varepsilon _v))^2 \pi _f^2\pi _m(\xi _f+\mu )(\xi _m+\mu )\mu ^2\{(\mu (1-\varepsilon _v q_m) + \xi _m(1-\varepsilon _v))\\&((1-\varepsilon _v)(\xi _m+\mu )+\mu )-\mathcal {R}_{vs}(1-\varepsilon _v)(\xi _m+\mu )\mu \}\\ \ge&(1-\mathcal {R}_{vs})[(\mu (1-\varepsilon _v q_m) + \xi _m(1-\varepsilon _v))^2 \pi _f\pi _m^2(\xi _f+\mu )(\xi _m+\mu )\mu ^2\{(1-\varepsilon _v)(\xi _f+\mu )\mu \\&+ (\mu (1-\varepsilon _vq_f)+\xi _f(1-\varepsilon _v))((1-\varepsilon _v)(\xi _f+\mu )+\mu )\} + \mathcal {R}_f(\mu (1-\varepsilon _v q_f)\\&+ \xi _f(1-\varepsilon _v))^2 \pi _f^2\pi _m\\&(\xi _f+\mu )(\xi _m+\mu )\mu ^2(1-\varepsilon _v)(\xi _m+\mu )\mu ] \quad \ge 0 \;\bigg ( \;\text{ if }\; \mathcal {R}_{vs}\le 1 \;\bigg ),\\ a_0 =&(\mu (1-\varepsilon _vq_f) + \xi _f(1-\varepsilon _v))(\mu (1-\varepsilon _v q_m) \\ &+ \xi _m(1-\varepsilon _v))^2\pi _f\pi _m^2(\xi _f+\mu )^2(\xi _m+\mu )\mu ^3[1-\mathcal {R}_f\mathcal {R}_m] \\ \ge&~0 \;\bigg ( \;\text{ if } \text{ and } \text{ only } \text{ if }\; \mathcal {R}_{vs}\le 1 \;\bigg ). \end{aligned}$$

It should be mentioned that the inequalities above use the fact that (for $i=f,m$):

$$\mu (1-\varepsilon _vq_i)+\xi _i(1-\varepsilon _v) \ge (\mu +\xi _i)(1-\varepsilon _v),$$

since $$0\le q_i\le 1$$.

## Proof of Theorem 2.6

### Proof

Consider the special case of the model (2.5) with negligible disease-induced mortality ($$\delta = \delta _d = 0$$) and a perfect vaccine ($$\varepsilon _v=1$$). Then, the coefficients of the polynomial (2.20) satisfy $$a_4=a_3=0$$ and $$a_2\ge 0$$, hence the quartic equation becomes quadratic in $$\lambda _{mf}^{**}$$. In this case, the special case of the model (with $$\delta =\delta _d=0$$ and $$\varepsilon _v=1$$) has a unique endemic equilibrium if $$a_0 < 0$$ (i.e., $$\tilde{\mathcal {R}}_{vs}>1$$), by the Descartes’ Rule of Signs. It is convenient to define this unique endemic equilibrium by $$\tilde{\mathcal {E}}_{1s} = \mathcal {E}_{1s}|_{\varepsilon _v=1}$$. The local asymptotic stability of the associated unique endemic equilibrium (EE) of the special case of the model can be established, when $$\tilde{\mathcal {R}}_{vs}>1$$, using the Krasnoselskii sub-linearity approach (as in (Esteva and Vargas 2000; Hethcote and Thieme 1985; Pant and Gumel 2024; Thieme 1985)). The method is based on the fact that, for a given $$n-$$dimensional system of nonlinear differential equations of the form $$\dot{x} = f(x)$$, its equilibrium $$\mathcal {E}$$ is locally-asymptotically stable if its corresponding linearized system, $$\dot{Z} = Df(\mathcal {E})Z$$ does not have a solution of the form $$Z(t) = \textbf{Z}e^{\omega t}$$, where $$\textbf{Z}\in \mathbb {C}^n\setminus \{0\}$$, $$\omega \in \mathbb {C}$$, and Re$$(\omega ) \ge 0$$. Since $$\delta =\delta _d=0$$, it follows that the total female ($$N_f(t)$$) and male ($$N_m(t)$$) populations at time *t* are asymptotically constant. That is, $$(N_f(t), N_m(t)) \rightarrow (N_f^*, N_m^*)$$, as $$t \rightarrow \infty $$. Substituting $$N_m^*$$ and $$N_f^*$$ for $$N_m(t)$$ and $$N_f(t)$$ in the model shows that,

D.1 $$\begin{aligned}&S_{f} = N_{f}^{*}-V_{f}-E_{f}-I_{f}-P_{f}-Q_{1u}-Q_{2u}-Q_{3u}-Q_{1d}-Q_{2d}\nonumber \\&-Q_{3d}-C_{u}-C_{d}-R_{f}^{C}-R_{f},\nonumber \\&S_m = N_m^{*}-V_m-E_m-I_m-P_m-J_{1m}-J_{2m}-J_{3m}-C_m-R_m^{C}-R_m. \end{aligned}$$

Using (D.1), the special case of the model (2.5), with $\delta =\delta _d=0$ and $\varepsilon _v=1$, can be rewritten as:

D.2 $$\begin{aligned} \begin{aligned}&{Females} \left\{ \begin{array}{lcl} \dot{V}_f & =& q_f \pi _f + \xi _f (N_{f}^{*}-V_{f}-E_{f}-I_{f}-P_{f}-Q_{1u}-Q_{2u}-Q_{3u}-Q_{1d} \\  & & -Q_{2d}-Q_{3d}-C_{u}-C_{d}-R_{f}^{C}-R_{f}) - \mu V_f, \vspace{2mm}\\ \dot{E}_f & =& \lambda _{mf} (N_{f}^{*}-V_{f}-E_{f}-I_{f}-P_{f}-Q_{1u}-Q_{2u}-Q_{3u}-Q_{1d} \\  & & -Q_{2d}-Q_{3d}-C_{u}-C_{d}-R_{f}^{C}-R_{f}) - k_1 E_f, \vspace{2mm}\\ \dot{I}_f & =& \sigma _f E_f - k_2 I_f, \vspace{2mm}\\ \dot{P}_f & =& l_1 I_f - k_3 P_f, \vspace{2mm}\\ \dot{Q}_{1u} & =& l_2 P_f + h_{u2}y_f Q_{2u} -k_4 Q_{1u}, \vspace{2mm}\\ \dot{Q}_{2u} & =& l_3 Q_{1u} +q_{u2}z_fQ_{3u} -k_5 Q_{2u}, \vspace{2mm}\\ \dot{Q}_{3u} & =& l_4 Q_{2u}-k_6 Q_{3u}, \vspace{2mm}\\ \dot{Q}_{1d} & =& d_{u3}g_f Q_{1u} + h_{d2} \zeta _2 Q_{2d} -k_7 Q_{1d}, \vspace{2mm}\\ \dot{Q}_{2d} & =& h_{u3}y_f Q_{2u} +l_6 Q_{1d} + q_{d2}\zeta _3 Q_{3d} -k_8 Q_{2d}, \vspace{2mm}\\ \dot{Q}_{3d} & =& q_{u3} z_f Q_{3u} + l_7 Q_{2d} -k_9 Q_{3d}, \vspace{2mm}\\ \dot{C}_u & =& l_5 Q_{3u} -k_{10} C_u, \vspace{2mm} \\ \dot{C}_d & =& l_8 Q_{3d} + w_f C_u -k_{11} C_d, \vspace{2mm} \\ \dot{R}_f^C & =& r_u C_u + r_d C_d -\mu R_f^C, \vspace{2mm}\\ \dot{R}_f & =& b_f \psi _f I_f + k_f \alpha _f P_f + d_{u1}g_f Q_{1u} + h_{u1}y_f Q_{2u} + q_{u1}z_f Q_{3u} + d_{d1} \zeta _1 Q_{1d} \\  & & + h_{d1}\zeta _2 Q_{2d} + q_{d1} \zeta _3 Q_{3d} -\mu R_f, \end{array} \right. \\\\&{Males} \left\{ \begin{array}{lcl} \dot{V}_m & =& q_m\pi _m +\xi _m(N_m^{*}-V_m-E_m-I_m-P_m-J_{1m}-J_{2m}-J_{3m} \\  & & -C_m-R_m^{C}-R_m) - \mu V_m, \vspace{2mm}\\ \dot{E}_m & =& \lambda _{fm} (N_m^{*}-V_m-E_m-I_m-P_m-J_{1m}-J_{2m}-J_{3m} \\  & & -C_m-R_m^{C}-R_m) - e_1E_m, \vspace{2mm} \\ \dot{I}_m & =& \sigma _m E_m - e_2 I_m, \vspace{2mm}\\ \dot{P}_m & =& (1-b_m)\psi _m I_m - e_3P_m, \vspace{2mm}\\ \dot{J}_{1m} & =& (1-k_m)\alpha _m P_m + h_{m2}y_m J_{2m}- e_4J_{1m}, \vspace{2mm} \\ \dot{J}_{2m} & =& (1-d_m)g_m J_{1m} + q_{m2}z_m J_{3m}- e_5J_{2m}, \vspace{2mm} \\ \dot{J}_{3m} & =& f_1 J_{2m}-e_6 J_{3m}, \vspace{2mm} \\ \dot{C}_m & =& f_2 J_{3m} -e_7 C_m, \vspace{2mm} \\ \dot{R}_m^C & =& r_m C_m -\mu R_m^C, \vspace{2mm}\\ \dot{R}_m & =& b_m \psi _m I_m + k_m \alpha _m P_m + d_mg_m J_{1m} + h_{m1}y_m J_{2m} + q_{m1}z_m J_{3m}- \mu R_m, \end{array} \right. \end{aligned} \end{aligned}$$

where the associated forces of infection, $\lambda _{mf}$ and $\lambda _{fm}$, obtained by substituting $N_m = N_m^{*}$ in the special case of the model, are given by:

$$\begin{aligned}&\lambda _{mf} = \left( \beta _{mf} c_{mf}\right) \left[ \frac{I_m + \eta _m E_m + \theta _m [P_m + J_{1m} + \kappa _m(J_{2m} + J_{3m})]}{N_m^*}\right] , \\&\lambda _{fm} = \left( \beta _{fm} c_{mf}\right) \\&\quad \left[ \frac{I_f + \eta _{f}E_f + \theta _{f} [P_f + Q_{1u} + \kappa _{f}(Q_{2u} + Q_{3u}) + \nu \{Q_{1d} + \kappa _{fd}(Q_{2d} + Q_{3d})\}]}{N_m^*}\right] , \end{aligned}$$

and $k_1 = \sigma _f+\mu , k_2 = \psi _f + \mu , k_3 = \alpha _f + \mu , k_4 = g_f+\mu , k_5 = y_f+\mu , k_6 = z_f+\mu , k_7 = \zeta _1+\mu , k_8 = \zeta _2+\mu , k_9 = \zeta _3+\mu , k_{10} = w_f+r_u+\delta +\mu , k_{11} = r_d+\delta _d+\mu , l_1 = (1-b_f)\psi _f, l_2 = (1-k_f)\alpha _f, l_3 = (1-d_{u1}-d_{u3})g_f, l_4 = (1-h_{u1}-h_{u2}-h_{u3})y_f, l_5 = (1-q_{u1}-q_{u2}-q_{u3})z_f, l_6 = (1-d_{d1})\zeta _1, l_7 = (1-h_{d1}-h_{d2})\zeta _2, l_8 = (1-q_{d1}-q_{d2})\zeta _3, e_1 = \sigma _m + \mu , e_2 = \psi _m + \mu , e_3 = \alpha _m + \mu , e_4 = g_m+\mu , e_5 = y_m+\mu , e_6 = z_m+\mu , e_7 = r_m+\mu , f_1 = (1-h_{m1}-h_{m2})y_m, f_2 = (1-q_{m1}-q_{m2})z_m$ are as given in Section 2.2.1. Suppose that the linearized system

D.3 $$\begin{aligned} \dot{Z} = J(\tilde{\mathcal {E}}_{1s})Z \end{aligned}$$

around the unique endemic equilibrium $\tilde{\mathcal {E}}_{1s}$ has a solution of the form:

D.4 $$\begin{aligned} Z(t) = \textbf{Z}e^{\omega t}, \end{aligned}$$

with $\textbf{Z} = (Z_1, Z_2, \cdots , Z_{24}) \in \mathbb {C}^{24}\setminus \{0\}, ~\omega \in \mathbb {C}$ (i.e., assume that *Z*(*t*) is a nontrivial solution). Substituting the solution of the form (D.4) into the linearized system (D.3) gives

D.5 $$\begin{aligned} \omega Z_1 =&~ (-\xi _f-\mu )Z_1 -\xi _f \sum _{i=2}^{14} Z_i, \nonumber \\\omega Z_2 =&~ -\lambda _{mf}^{**}Z_1 +(-\lambda _{mf}^{**}-k_1)Z_2\nonumber \\&-\lambda _{mf}^{**}\sum _{i=3}^{14} Z_i +a_1 Z_{16} +a_2Z_{17} + a_3 Z_{18} +a_3 Z_{19} \nonumber \\&+a_4 Z_{20} +a_4 Z_{21}, \nonumber \\ \omega Z_3 =&~ \sigma _f Z_2-k_2Z_3,\nonumber \\ \omega Z_4 =&~ l_1Z_3 -k_3Z_4,\nonumber \\ \omega Z_5 =&~ l_2Z_4-k_4Z_5+h_{u2}y_fZ_6,\nonumber \\ \omega Z_6 =&~ l_3Z_5 -k_5Z_6 +q_{u2}z_fZ_7,\nonumber \\ \omega Z_7 =&~ l_4Z_6 -k_6Z_7,\nonumber \\ \omega Z_8 =&~ d_{u3}g_fZ_5 -k_7Z_8 +h_{d2}\zeta _2Z_9,\nonumber \\ \omega Z_9 =&~ h_{u3}y_fZ_6 +l_6Z_8 -k_8Z_9 +q_{d2}\zeta _3Z_{10},\nonumber \\ \omega Z_{10} =&~ q_{u3}z_fZ_7 +l_7Z_9 -k_9Z_{10},\nonumber \\ \omega Z_{11} =&~ l_5Z_7 -k_{10}Z_{11},\nonumber \\ \omega Z_{12} =&~ l_8Z_{10} +w_fZ_{11} -k_{11}Z_{12},\\ \omega Z_{13} =&~ r_uZ_{11} +r_dZ_{12} -\mu Z_{13},\nonumber \\ \omega Z_{14} =&~ b_f\psi _fZ_3 +k_f\alpha _fZ_4 +d_{u1}g_fZ_5 +h_{u1}y_fZ_6 +q_{u1}z_fZ_7\nonumber \\&+d_{d1}\zeta _1Z_8 +h_{d1}\zeta _2Z_9 \nonumber \\&+q_{d1}\zeta _3Z_{10} -\mu Z_{14},\nonumber \\ \omega Z_{15} =&~ (-\xi _m-\mu )Z_{15} -\xi _m\sum _{i=16}^{24} Z_i,\nonumber \\ \omega Z_{16} =&~ b_1Z_2 +b_2Z_3 +b_3Z_4 +b_3Z_5 +b_4Z_6 +b_4Z_7 +b_5Z_8 +b_6Z_9 +b_6Z_{10} \nonumber \\&-\lambda _{fm}^{**}Z_{15} +(-\lambda _{fm}^{**}-e_1)Z_{16} -\lambda _{fm}^{**}\sum _{i=17}^{24} Z_i, \nonumber \\ \omega Z_{17} =&~ \sigma _m Z_{16}-e_2Z_{17},\nonumber \\ \omega Z_{18} =&~ (1-b_m)\psi _mZ_{17} -e_3Z_{18},\nonumber \\ \omega Z_{19} =&~ (1-k_m)\alpha _mZ_{18}-e_4Z_{19}+h_{m2}y_mZ_{20},\nonumber \\ \omega Z_{20} =&~ (1-d_m)g_mZ_{19} -e_5Z_{20} +q_{m2}z_mZ_{21},\nonumber \\ \omega Z_{21} =&~ f_1Z_{20} -e_6Z_{21},\nonumber \\ \omega Z_{22} =&~ f_2Z_{21} -e_7Z_{22},\nonumber \\ \omega Z_{23} =&~ r_mZ_{22} -\mu Z_{23},\nonumber \\ \omega Z_{24} =&~ b_m\psi _mZ_{17} +k_m\alpha _mZ_{18} +d_mg_mZ_{19} +h_{m1}y_mZ_{20} +q_{m1}z_mZ_{21} -\mu Z_{24},\nonumber \end{aligned}$$

where $$a_1 = \frac{\beta _{mf}c_{mf}\eta _m S_{f}^{**}}{N_m^*}$$, $$a_2 = \frac{\beta _{mf}c_{mf} S_{f}^{**}}{N_m^*}$$, $$a_3 = \frac{\beta _{mf}c_{mf}\theta _m S_{f}^{**}}{N_m^*}$$, $$a_4 = \frac{\beta _{mf}c_{mf}\theta _m\kappa _m S_{f}^{**}}{N_m^*}$$, $$b_1 = \frac{\beta _{fm}c_{mf}\eta _f S_m^{**}}{N_m^*}$$, $$b_2 = \frac{\beta _{fm}c_{mf} S_m^{**}}{N_m^*}$$, $$b_3 = \frac{\beta _{fm}c_{mf}\theta _f S_m^{**}}{N_m^*}$$, $$b_4 = \frac{\beta _{fm}c_{mf}\theta _f\kappa _f S_m^{**}}{N_m^*}, b_5 = \frac{\beta _{fm}c_{mf}\theta _f\nu S_m^{**}}{N_m^*}, b_6 = \frac{\beta _{fm}c_{mf}\theta _f\nu \kappa _{fd} S_m^{**}}{N_m^*}$$. The third to the 14th equations of the system (D.5) can be rewritten, by moving their negative terms to the left-hand side and dividing by their respective coefficients, as:

D.6 $$\begin{aligned} \left( 1+\frac{\omega }{k_2}\right) Z_3= &  \frac{\sigma _f}{k_2}Z_2, ~~\left( 1+\frac{\omega }{k_3}\right) Z_4 = \frac{l_1}{k_3}Z_3,\nonumber \\ \left( 1+\frac{\omega }{k_4}\right) Z_5= &  \frac{l_2}{k_4}Z_4 + \frac{h_{u2}y_f}{k_4}Z_6, ~~\left( 1+\frac{\omega }{k_5}\right) Z_6 = \frac{l_3}{k_5}Z_5 +\frac{q_{u2}z_f}{k_5}Z_7,\nonumber \\ \left( 1+\frac{\omega }{k_6}\right) Z_7= &  \frac{l_4}{k_6}Z_6, ~~\left( 1+\frac{\omega }{k_7}\right) Z_8 = \frac{d_{u3}g_f}{k_7}Z_5 +\frac{h_{d2}\zeta _2}{k_7}Z_9,\nonumber \\ \left( 1+\frac{\omega }{k_8}\right) Z_9= &  \frac{h_{u3}y_f}{k_8}Z_6 +\frac{l_6}{k_8}Z_8 +\frac{q_{d2}\zeta _3}{k_8}Z_{10}, ~~\left( 1+\frac{\omega }{k_9}\right) \nonumber \\ &  Z_{10} = \frac{q_{u3}z_f}{k_9}Z_7 +\frac{l_7}{k_9}Z_9,\nonumber \\ \left( 1+\frac{\omega }{k_{10}}\right) Z_{11}= &  \frac{l_5}{k_{10}}Z_7, ~~\left( 1+\frac{\omega }{k_{11}}\right) Z_{12} = \frac{l_8}{k_{11}}Z_{10} +\frac{w_f}{k_{11}}Z_{11},\nonumber \\ \left( 1+\frac{\omega }{\mu }\right) Z_{13}= &  \frac{r_u}{\mu }Z_{11} +\frac{r_d}{\mu }Z_{12},\nonumber \\ \left( 1+\frac{\omega }{\mu }\right) Z_{14}= &  \frac{b_f\psi _f}{\mu }Z_3 +\frac{k_f\alpha _f}{\mu }Z_4 +\frac{d_{u1}g_f}{\mu }Z_5 +\frac{h_{u1}y_f}{\mu }\nonumber \\ &  Z_6 +\frac{q_{u1}z_f}{\mu }Z_7 +\frac{d_{d1}\zeta _1}{\mu }Z_8 \nonumber \\  &  +\frac{h_{d1}\zeta _2}{\mu }Z_9 +\frac{q_{d1}\zeta _3}{\mu }Z_{10}. \end{aligned}$$

Similarly, the 17th through the 24th equations of the system (D.5) can be rewritten as:

D.7 $$\begin{aligned} \begin{aligned} \left( 1+\frac{\omega }{e_2}\right) Z_{17}&= \frac{\sigma _m}{e_2}Z_{16}, ~~\left( 1+\frac{\omega }{e_3}\right) Z_{18} = \frac{(1-b_m)\psi _m}{e_3}Z_{17},\\ \left( 1+\frac{\omega }{e_4}\right) Z_{19}&= \frac{(1-k_m)\alpha _m}{e_4}Z_{18} + \frac{h_{m2}y_m}{e_4}Z_{20}, \\ \left( 1+\frac{\omega }{e_5}\right) Z_{20}&= \frac{(1-d_m)g_m}{e_5}Z_{19} +\frac{q_{m2}z_m}{e_5}Z_{21},\\ \left( 1+\frac{\omega }{e_6}\right) Z_{21}&= \frac{f_1}{e_6}Z_{20}, ~~\left( 1+\frac{\omega }{e_7}\right) Z_{22} = \frac{f_2}{e_7}Z_{21},\\ \left( 1+\frac{\omega }{\mu }\right) Z_{23}&= \frac{r_m}{\mu }Z_{22},\\ \left( 1+\frac{\omega }{\mu }\right) Z_{24}&= \frac{b_m\psi _m}{\mu }Z_{17} +\frac{k_m\alpha _m}{\mu }Z_{18} +\frac{d_mg_m}{\mu }Z_{19} +\frac{h_{m1}y_m}{\mu }Z_{20} +\frac{q_{m1}z_m}{\mu }Z_{21}. \end{aligned} \end{aligned}$$

To get a similar expression from the first and second equations of the system (D.5), all equations in (D.6) are rewritten in terms of $Z_2$ as:

D.8 $$\begin{aligned} Z_3&= \frac{\sigma _f}{\omega +k_2}Z_2&=: A_1Z_2,\nonumber \\ Z_4&= \frac{\sigma _fl_1}{(\omega +k_2)(\omega +k_3)}Z_2&=: A_2Z_2,\nonumber \\ Z_5&= \frac{\sigma _fl_1l_2(\omega +k_5)[\omega ^2+(k_5+k_6)\omega +\iota _{f2}]}{(\omega +k_2)(\omega +k_3)B}Z_2&=: A_3Z_2,\nonumber \\ Z_6&= \frac{\sigma _fl_1l_2l_3(\omega +k_6)}{(\omega +k_2)(\omega +k_3)B}Z_2&=: A_4Z_2,\nonumber \\ Z_7&= \frac{\sigma _fl_1l_2l_3l_4}{(\omega +k_2)(\omega +k_3)B}Z_2&=: A_5Z_2,\nonumber \\ Z_8&= \left( \frac{d_{u3}g_f[\omega ^2 +(k_8+k_9)\omega +(\iota _{f3} +l_6h_{d2}\zeta _2k_9)/k_7]}{C}A_3 \right.&\nonumber \\&\hspace{4mm} \left. +\frac{(\omega +k_9)h_{u3}y_fh_{d2}\zeta _2}{C}A_4+\frac{q_{u3}z_fq_{d2}\zeta _3h_{d2}\zeta _2}{C}A_5\right) Z_2&=: A_6Z_2,\nonumber \\ Z_9&= \frac{(\omega +k_9)h_{u3}y_fA_4 +q_{d2}\zeta _3q_{u3}z_fA_5 +(\omega +k_9)l_6A_6}{\omega ^2 +(k_8+k_9)\omega +(\iota _{f3}+l_6h_{d2}\zeta _2k_9)/k_7}Z_2&=: A_7Z_2,\nonumber \\ Z_{10}&= \left( \frac{q_{u3}z_f}{\omega +k_9}A_5 +\frac{l_7}{\omega +k_9}A_7 \right) Z_2&=: A_8Z_2,\nonumber \\ Z_{11}&= \frac{\sigma _fl_1l_2l_3l_4l_5}{(\omega +k_2)(\omega +k_3)B(\omega +k_{10})}Z_2&=: A_9Z_2,\nonumber \\ Z_{12}&= \left( \frac{l_8}{\omega +k_{11}}A_8 +\frac{w_f}{\omega +k_{11}}A_9 \right) Z_2&=: A_{10}Z_2,\nonumber \\ Z_{13}&= \left( \frac{r_u}{\omega +\mu }A_9 +\frac{r_d}{\omega +\mu }A_{10} \right) Z_2&=: A_{11}Z_2,\nonumber \\ Z_{14}&= \left( \frac{b_f\psi _f}{\omega +\mu }A_1 +\frac{k_f\alpha _f}{\omega +\mu }A_2 +\frac{d_{u1}g_f}{\omega +\mu }A_3 +\frac{h_{u1}y_f}{\omega +\mu }A_4 +\frac{q_{u1}z_f}{\omega +\mu }A_5 \right.&\nonumber \\&\hspace{4mm} \left. +\frac{d_{d1}\zeta _1}{\omega +\mu }A_6 +\frac{h_{d1}\zeta _2}{\omega +\mu }A_7 +\frac{q_{d1}\zeta _3}{\omega +\mu }A_8 \right) Z_2&=: A_{12}Z_2,\nonumber \\ \end{aligned}$$

where,

$$\begin{aligned} B&= (\omega +k_4)(\omega +k_5)(\omega +k_6)-(\omega +k_4)l_4q_{u2}z_f-l_3h_{u2}y_f(\omega +k_6) \\&= \omega ^3 +(k_4+k_5+k_6) \omega ^2 +(\iota _{f1}+k_4l_4q_{u2}z_f) \omega /k_6 +(\iota _{f2} +k_6k_4) \omega +\iota _{f1},\\ C&= (\omega +k_7)(\omega +k_8)(\omega +k_9)-(\omega +k_7)l_7q_{d2}\zeta _3-l_6h_{d2}\zeta _2(\omega +k_9) \\&= \omega ^3 +(k_7+k_8+k_9) \omega ^2 +(\iota _{f3}+k_7l_7q_{d2}\zeta _3) \omega /k_9 \\ &\quad +(\iota _{f3}+l_6h_{d2}\zeta _2k_9)\omega /k_7 +k_9k_7\omega +\iota _{f3}, \end{aligned}$$

and $\iota _{f1} = k_4k_5k_6-l_3h_{u2}y_fk_6-k_4l_4q_{u2}z_f, \; \iota _{f2} = k_5k_6-l_4q_{u2}z_f, \; \iota _{f3} = k_7k_8k_9-l_6h_{d2}\zeta _2k_9-k_7l_7q_{d2}\zeta _3$ are as given in Section 2.2.1. It should be noted that $\iota _{f1}$, $\iota _{f2}$, and $\iota _{f3}$ are non-negative (as shown in Appendix B). Hence (note also the aforementioned definitions of $k_4, k_5, k_6, k_7, k_8, k_9, l_3, l_4, l_6, l_7$), *B* and *C* have non-negative coefficients. Then, solving for $Z_1$ from the first equation of the system (D.5) gives:

D.9 $$\begin{aligned} Z_1 = -\left( \frac{\xi _f}{\omega +\xi _f+\mu }\right) \sum _{i=2}^{14}Z_i. \end{aligned}$$

The second equation of (D.5) can be rewritten (by moving its negative terms to the left-hand side and dividing with $k_1$) as:

D.10 $$\begin{aligned}&\frac{\omega }{k_1} Z_2 +\frac{\lambda _{mf}^{**}}{k_1}Z_1 +\left( \frac{\lambda _{mf}^{**}}{k_1}+1\right) Z_2\nonumber \\&\quad +\frac{\lambda _{mf}^{**}}{k_1}\sum _{i=3}^{14} Z_i = \frac{a_1}{k_1}Z_{16} +\frac{a_2}{k_1}Z_{17}+\frac{a_3}{k_1}(Z_{18}+Z_{19}) +\frac{a_4}{k_1}(Z_{20}+Z_{21}). \end{aligned}$$

Then, substituting (D.8) and (D.9) into the left-hand side of (D.10) gives:

D.11 $$\begin{aligned}&\left[ 1+\frac{\omega }{k_1} +\frac{\lambda _{mf}^{**}(\omega +\mu )}{(\omega +\xi _f+\mu )k_1}\left( 1+\sum _{i=1}^{12}A_i\right) \right] Z_2 \nonumber \\&\quad = \frac{a_1}{k_1}Z_{16} +\frac{a_2}{k_1}Z_{17}+\frac{a_3}{k_1}(Z_{18}+Z_{19}) +\frac{a_4}{k_1}(Z_{20}+Z_{21}). \end{aligned}$$

To get a similar expression from the 15th and 16th equations of the system (D.5), the equations in (D.7) are rewritten in terms of $Z_{16}$ as follows:

D.12 $$\begin{aligned} Z_{17}&= \frac{\sigma _m}{\omega +e_2}Z_{16}&=: A_{13}Z_{16},\nonumber \\ Z_{18}&= \frac{\sigma _m(1-b_m)\psi _m}{(\omega +e_2)(\omega +e_3)}Z_{16}&=: A_{14}Z_{16},\nonumber \\ Z_{19}&= \frac{\sigma _m(1-b_m)\psi _m(1-k_m)\alpha _m[\omega ^2+(e_5+e_6)\omega +\iota _{m2}]}{(\omega +e_2)(\omega +e_3)D}Z_{16}&=: A_{15}Z_{16},\nonumber \\ Z_{20}&= \frac{\sigma _m(1-b_m)\psi _m(1-k_m)\alpha _m(1-d_m)g_m(\omega +e_6)}{(\omega +e_2)(\omega +e_3)D}Z_{16}&=: A_{16}Z_{16},\\ Z_{21}&= \frac{\sigma _m(1-b_m)\psi _m(1-k_m)\alpha _m(1-d_m)g_mf_1}{(\omega +e_2)(\omega +e_3)D}Z_{16}&=: A_{17}Z_{16},\nonumber \\ Z_{22}&= \frac{A_{17}f_2}{(\omega +e_7)}Z_{16}&=: A_{18}Z_{16},\nonumber \\ Z_{23}&= \frac{A_{17}f_2r_m}{(\omega +e_7)(\omega +\mu )}Z_{16}&=: A_{19}Z_{16},\nonumber \\ Z_{24}&= \left( \frac{b_m\psi _m}{\omega +\mu }A_{13} +\frac{k_m\alpha _m}{\omega +\mu }A_{14} +\frac{d_mg_m}{\omega +\mu }\right. \nonumber \\&\quad \left. A_{15} +\frac{h_{m1}y_m}{\omega +\mu }A_{16} +\frac{q_{m1}z_m}{\omega +\mu }A_{17}\right) Z_{16}&=: A_{20}Z_{16},\nonumber \end{aligned}$$

where,

$$\begin{aligned} D&= (\omega +e_4)(\omega +e_5)(\omega +e_6)-(\omega +e_4)f_1q_{m2}z_m-(1-d_m)g_mh_{m2}y_m(\omega +e_6) \\&= \omega ^3 +(e_4+e_5+e_6) \omega ^2 +(\iota _{m1}+e_4f_1q_{m2}z_m) \omega /e_6 +(\iota _{m2} +e_6e_4) \omega +\iota _{m1}, \end{aligned}$$

and $\iota _{m1} = e_4e_5e_6-(1-d_m)g_mh_{m2}y_me_6-e_4f_1q_{m2}z_m, \; \iota _{m2} = e_5e_6-f_1q_{m2}z_m$ are as given in Section 2.2.1. It should be noted that $\iota _{m1}$ and $\iota _{m2}$ are non-negative (as shown in Appendix B). Hence (note also the aforementioned definitions of $e_4, e_5, e_6, f_1$), all the coefficients of *D* are non-negative. Then, solving for $Z_{15}$ from the 15th equation of the system (D.5) gives:

D.13 $$\begin{aligned} Z_{15} = -\left( \frac{\xi _m}{\omega +\xi _m+\mu }\right) \sum _{i=16}^{24}Z_i. \end{aligned}$$

The 16th equation of (D.5) can also be rewritten (by moving its negative terms to the left-hand side and dividing with $e_1$) as:

D.14 $$\begin{aligned}&\frac{\omega }{e_1} Z_{16} \!+\!\frac{\lambda _{fm}^{**}}{e_1}Z_{15} \!+\!\left( \frac{\lambda _{fm}^{**}}{e_1}\!+\!1\right) Z_{16} \!+\!\frac{\lambda _{fm}^{**}}{e_1}\!\sum _{i=17}^{24} Z_i \!=\!\frac{b_1}{e_1}Z_2 \!+\!\frac{b_2}{e_1}Z_3 \!+\!\frac{b_3}{e_1}(Z_4+Z_5)\\&\quad +\frac{b_4}{e_1}(Z_6+Z_7) +\frac{b_5}{e_1}Z_8 +\frac{b_6}{e_1}(Z_9+Z_{10}).\nonumber \end{aligned}$$

Then, substituting (D.12) and (D.13) into the left-hand side of (D.14) gives:

D.15 $$\begin{aligned}&\left[ 1+\frac{\omega }{e_1} +\frac{\lambda _{fm}^{**}(\omega +\mu )}{(\omega +\xi _m+\mu )e_1}\left( 1+\sum _{i=13}^{20}A_i\right) \right] Z_{16} = ~\frac{b_1}{e_1}Z_2 +\frac{b_2}{e_1}Z_3 +\frac{b_3}{e_1}(Z_4+Z_5)\\&\quad +\frac{b_4}{e_1}(Z_6+Z_7) +\frac{b_5}{e_1}Z_8 +\frac{b_6}{e_1}(Z_9+Z_{10}).\nonumber \end{aligned}$$

Hence, equations (D.6), (D.7), (D.11), and (D.15) can be rewritten in the following matrix-vector form (where $\textbf{Y} = (Z_2, Z_3, \cdots , Z_{12}, Z_{16}, Z_{17}, \cdots , Z_{22})$ is the projection of the vector $\textbf{Z}$ from $\mathbb {C}^{24}\setminus \{0\}$ to $\mathbb {C}^{18}\setminus \{0\}$):

D.16 $$\begin{aligned} [1+F_i(\omega )]\textbf{Y}_i = (H\textbf{Y})_i, ~i=1, 2, \cdots , 18 , \end{aligned}$$

where,

$$\begin{aligned} \begin{aligned} F_1(\omega )&= \frac{\omega }{k_1} +\frac{\lambda _{mf}^{**}(\omega +\mu )}{(\omega +\xi _f+\mu )k_1}\left( 1+\sum _{i=1}^{12}A_i\right) , ~F_j(\omega ) = \frac{\omega }{k_j} ~(j = 2,3, \cdots , 11),\\ F_{12}(\omega )&= \frac{\omega }{e_1} +\frac{\lambda _{fm}^{**}(\omega +\mu )}{(\omega +\xi _m+\mu )e_1}\left( 1+\sum _{i=13}^{20}A_i\right) , \\&\quad F_j(\omega ) = \frac{\omega }{e_{j-11}} ~(j = 13, 14, \cdots , 18), \end{aligned} \end{aligned}$$

and H is the non-negative $18\times 18$ matrix given below:

$$\begin{aligned}\left[ \begin{array}{c c c c c c c c c c c c c c c c c c} 0 & 0 & 0 & 0 & 0 & 0 & 0 & 0 & 0 & 0 & 0 & \frac{a_1}{k_1} & \frac{a_2}{k_1} & \frac{a_3}{k_1} & \frac{a_3}{k_1} & \frac{a_4}{k_1} & \frac{a_4}{k_1} & 0 \\ \frac{\sigma _f}{k_2} & 0 & 0 & 0 & 0 & 0 & 0 & 0 & 0 & 0 & 0 & 0 & 0 & 0 & 0 & 0 & 0 & 0\\ 0 & \frac{l_1}{k_3} & 0 & 0 & 0 & 0 & 0 & 0 & 0 & 0 & 0 & 0 & 0 & 0 & 0 & 0 & 0 & 0\\ 0 & 0 & \frac{l_2}{k_4} & 0 & \frac{h_{u2}y_f}{k_4} & 0 & 0 & 0 & 0 & 0 & 0 & 0 & 0 & 0 & 0 & 0 & 0 & 0\\ 0 & 0 & 0 & \frac{l_3}{k_5} & 0 & \frac{q_{u2}z_f}{k_5} & 0 & 0 & 0 & 0 & 0 & 0 & 0 & 0 & 0 & 0 & 0 & 0\\ 0 & 0 & 0 & 0 & \frac{l_4}{k_6} & 0 & 0 & 0 & 0 & 0 & 0 & 0 & 0 & 0 & 0 & 0 & 0 & 0\\ 0 & 0 & 0 & \frac{d_{u3}g_f}{k_7} & 0 & 0 & 0 & \frac{h_{d2}\zeta _2}{k_7} & 0 & 0 & 0 & 0 & 0 & 0 & 0 & 0 & 0 & 0 \\ 0 & 0 & 0 & 0 & \frac{h_{u3}y_f}{k_8} & 0 & \frac{l_6}{k_8} & 0 & \frac{q_{d2}\zeta _3}{k_8} & 0 & 0 & 0 & 0 & 0 & 0 & 0 & 0 & 0 \\ 0 & 0 & 0 & 0 & 0 & \frac{q_{u3}z_f}{k_9} & 0 & \frac{l_7}{k_9} & 0 & 0 & 0 & 0 & 0 & 0 & 0 & 0 & 0 & 0 \\ 0 & 0 & 0 & 0 & 0 & \frac{l_5}{k_{10}} & 0 & 0 & 0 & 0 & 0 & 0 & 0 & 0 & 0 & 0 & 0 & 0 \\ 0 & 0 & 0 & 0 & 0 & 0 & 0 & 0 & \frac{l_8}{k_{11}} & \frac{w_f}{k_{11}} & 0 & 0 & 0 & 0 & 0 & 0 & 0 & 0 \\ \frac{b_1}{e_1} & \frac{b_2}{e_1} & \frac{b_3}{e_1} & \frac{b_3}{e_1} & \frac{b_4}{e_1} & \frac{b_4}{e_1} & \frac{b_5}{e_1} & \frac{b_6}{e_1} & \frac{b_6}{e_1} & 0 & 0 & 0 & 0 & 0 & 0 & 0 & 0 & 0 \\ 0 & 0 & 0 & 0 & 0 & 0 & 0 & 0 & 0 & 0 & 0 & \frac{\sigma _m}{e_2} & 0 & 0 & 0 & 0 & 0 & 0 \\ 0 & 0 & 0 & 0 & 0 & 0 & 0 & 0 & 0 & 0 & 0 & 0 & \frac{(1-b_m)\psi _m}{e_3} & 0 & 0 & 0 & 0 & 0\\ 0 & 0 & 0 & 0 & 0 & 0 & 0 & 0 & 0 & 0 & 0 & 0 & 0 & \frac{(1-k_m)\alpha _m}{e_4} & 0 & \frac{h_{m2}y_m}{e_4} & 0 & 0\\ 0 & 0 & 0 & 0 & 0 & 0 & 0 & 0 & 0 & 0 & 0 & 0 & 0 & 0 & \frac{(1-d_m)g_m}{e_5} & 0 & \frac{q_{m2}z_m}{e_5} & 0 \\ 0 & 0 & 0 & 0 & 0 & 0 & 0 & 0 & 0 & 0 & 0 & 0 & 0 & 0 & 0 & \frac{f_1}{e_6} & 0 & 0\\ 0 & 0 & 0 & 0 & 0 & 0 & 0 & 0 & 0 & 0 & 0 & 0 & 0 & 0 & 0 & 0 & \frac{f_2}{e_7} & 0 \end{array}\right] .\end{aligned}$$

Furthermore, it can be shown that the projection of the endemic equilibrium $\tilde{\mathcal {E}}_{1s}$, defined by

$$\begin{aligned} &  \tilde{\mathcal {E}}_{1s}^p := (E_{f}^{**},I_{f}^{**},P_{f}^{**},Q_{1u}^{**},Q_{2u}^{**},Q_{3u}^{**},Q_{1d}^{**},Q_{2d}^{**},Q_{3d}^{**},C_{u}^{**},C_{d}^{**},\\ &  \quad E_m^{**},I_m^{**},P_m^{**},J_{1m}^{**},J_{2m}^{**},J_{3m}^{**},C_m^{**}),\end{aligned}$$

satisfies

D.17 $$\begin{aligned} \tilde{\mathcal {E}}_{1s}^p =H\tilde{\mathcal {E}}_{1s}^p. \end{aligned}$$

Since all components of $\tilde{\mathcal {E}}_{1s}^p$ are strictly positive and $\textbf{Y}$ is a nonzero vector (otherwise, (D.5) shows that $\textbf{Z}$ is also a zero vector, which contradicts the assumption), there exists a minimal positive number s, depending on $\textbf{Y}$, such that

D.18 $$\begin{aligned} |\textbf{Y}| \le s\tilde{\mathcal {E}}_{1s}^p, \end{aligned}$$

where $|\textbf{Y}| = (|Z_2|, |Z_3|, \cdots , |Z_{12}|, |Z_{16}|, |Z_{17}|, \cdots , |Z_{22}|)$ and $|\cdot |$ is the norm in $\mathbb {C}$. Next, to show that Re$(\omega ) < 0$, we suppose the contrary. That is, we suppose that Re$(\omega ) \ge 0$, and consider the following two cases (Esteva and Vargas 2000; Pant and Gumel 2024).

**Case 1:** $$\omega =0$$.

Since plugging $\omega =0$ into (D.4) gives $Z(t)=\textbf{Z}$ and hence $\dot{Z}(t) = \textbf{0}$, it follows that (D.3) is a homogeneous linear system $J(\tilde{\mathcal {E}}_{1s})\textbf{Z} = \textbf{0}$, and its determinant is given by

D.19 $$\begin{aligned} \Delta&= k_1k_2k_3\iota _{f1}\iota _{f3}e_1e_2e_3\iota _{m1}\left( 1-\mathcal {R}_{vs}^2\frac{S_m^{**}S_f^{**}}{S_m^*S_f^*}\right) (\mu +\xi _f)(\mu +\xi _m)k_{10}k_{11}e_7\mu ^4\nonumber \\&\quad +\left[ \lambda _{fm}^{**}G_mk_1k_2k_3\iota _{f1}\iota _{f3}(\mu +\xi _f) +\lambda _{mf}^{**}G_fe_1e_2e_3\iota _{m1}(\mu +\xi _m)\right] k_{10}k_{11}e_7\mu ^4\nonumber \\&\quad +\lambda _{fm}^{**}\lambda _{mf}^{**}G_mG_f, \end{aligned}$$

where,

$$\begin{aligned} G_m&= e_2e_3\iota _{m1}\mu +\sigma _me_3\iota _{m1}(\mu +b_m\psi _m) \\&+\sigma _m(1-b_m)\psi _m\iota _{m1}(\mu +k_m\alpha _m) +\sigma _m(1-b_m)\psi _m(1-k_m)\alpha _m\iota _{m2}(\mu +d_mg_m) \\&+\sigma _m(1-b_m)\psi _m(1-k_m)\alpha _m(1-d_m)g_m\{(e_6+f_1)\mu \\&+f_1q_{m1}z_m +h_{m1}y_me_6 +f_1f_2\},\\ G_f&= k_2k_3\iota _{f1}\iota _{f3}\mu +\sigma _fl_1l_2l_3l_4l_5\iota _{f3} +\sigma _fl_1l_2l_8j_{f3} \\ &+\sigma _fk_3\iota _{f1}\iota _{f3}(\mu +b_f\psi _f) \\&+\sigma _fl_1\iota _{f1}\iota _{f3}(\mu +k_f\alpha _f)\\&+\sigma _fl_1l_2\iota _{f2}\iota _{f3}(\mu +d_{u1}g_f) +\sigma _fl_1l_2l_3(l_4+k_6)\iota _{f3}(\mu +h_{u1}y_f) \\&+\sigma _fl_1l_2\{(j_{f1}+j_{f2}+j_{f3})\mu +j_{f1}d_{d1}\zeta _1 +j_{f2}h_{d1}\zeta _2 +j_{f3}q_{d1}\zeta _3\}. \end{aligned}$$

To determine the sign of $\Delta$, the system (D.2) is solved at the endemic equilibrium $\tilde{\mathcal {E}}_{1s}$ as:

$$\begin{aligned}&\lambda _{mf}^{**}S_{f}^{**} = k_1E_{f}^{**},\\&I_{f}^{**} = \frac{\sigma _f}{k_2}E_{f}^{**}, \; P_{f}^{**} = \frac{\sigma _fl_1}{k_2k_3}E_{f}^{**}, \; Q_{1u}^{**} = \frac{\sigma _fl_1l_2\iota _{f2}}{k_2k_3\iota _{f1}}E_{f}^{**}, \; Q_{2u}^{**} = \frac{\sigma _fl_1l_2l_3k_6}{k_2k_3\iota _{f1}}E_{f}^{**}, \\&Q_{3u}^{**} = \frac{\sigma _fl_1l_2l_3l_4}{k_2k_3\iota _{f1}}E_{f}^{**}, \; Q_{1d}^{**} = \frac{\sigma _fl_1l_2j_{f1}}{k_2k_3\iota _{f1}\iota _{f3}}E_{f}^{**}, \; Q_{2d}^{**} = \frac{\sigma _fl_1l_2j_{f2}}{k_2k_3\iota _{f1}\iota _{f3}}E_{f}^{**}, \\&\quad Q_{3d}^{**} = \frac{\sigma _fl_1l_2j_{f3}}{k_2k_3\iota _{f1}\iota _{f3}}E_{f}^{**},\\&\lambda _{fm}^{**}S_m^{**} = e_1E_m^{**},\\&I_m^{**} = \frac{\sigma _m}{e_2}E_m^{**}, \; P_m^{**} = \frac{\sigma _m(1-b_m)\psi _m}{e_2e_3}E_m^{**}, \\&J_{1m}^{**} = \frac{\sigma _m(1-b_m)\psi _m(1-k_m)\alpha _m\iota _{m2}}{e_2e_3\iota _{m1}}E_m^{**},\\&J_{2m}^{**} = \frac{\sigma _m(1-b_m)\psi _m(1-k_m)\alpha _m(1-d_m)g_me_6}{e_2e_3\iota _{m1}}E_m^{**}, \\&J_{3m}^{**} = \frac{\sigma _m(1-b_m)\psi _m(1-k_m)\alpha _m(1-d_m)g_mf_1}{e_2e_3\iota _{m1}}E_m^{**}. \end{aligned}$$

Then, we observe that

$$\begin{aligned} \lambda _{mf}^{**}S_{f}^{**}&= a_1E_m^{**} +a_2I_m^{**} +a_3(P_m^{**}+J_{1m}^{**}) +a_4(J_{2m}^{**}+J_{3m}^{**})\\&= \left[ a_1 +a_2\frac{\sigma _m}{e_2} +a_3\frac{\sigma _m(1-b_m)\psi _m}{e_2e_3} +a_3\frac{\sigma _m(1-b_m)\psi _m(1-k_m)\alpha _m\iota _{m2}}{e_2e_3\iota _{m1}}\right. \\&\hspace{4mm}+\left. a_4\frac{\sigma _m(1-b_m)\psi _m(1-k_m)\alpha _m(1-d_m)g_m(e_6+f_1)}{e_2e_3\iota _{m1}}\right] E_m^{**}\\&= e_1\mathcal {R}_m\frac{S_{f}^{**}}{S_{f}^*}E_m^{**} = k_1E_{f}^{**},\\ \lambda _{fm}^{**}S_m^{**}&= b_1E_{f}^{**} +b_2I_{f}^{**} +b_3(P_{f}^{**}+Q_{1u}^{**})\\&+b_4(Q_{2u}^{**}+Q_{3u}^{**}) +b_5Q_{1d}^{**} +b_6(Q_{2d}^{**}+Q_{3d}^{**})\\&= \left[ b_1 +b_2\frac{\sigma _f}{k_2} +b_3\frac{\sigma _fl_1}{k_2k_3} +b_3\frac{\sigma _fl_1l_2\iota _{f2}}{k_2k_3\iota _{f1}}\right. +b_4\frac{\sigma _fl_1l_2l_3(k_6+l_4)}{k_2k_3\iota _{f1}}\\&\hspace{4mm}+\left. b_5\frac{\sigma _fl_1l_2j_{f1}}{k_2k_3\iota _{f1}\iota _{f3}} +b_6\frac{\sigma _fl_1l_2(j_{f2}+j_{f3})}{k_2k_3\iota _{f1}\iota _{f3}}\right] E_{f}^{**}\\&= k_1\mathcal {R}_f\frac{S_m^{**}}{S_m^*}E_{f}^{**} = e_1E_m^{**}. \end{aligned}$$

Since $$e_1E_m^{**}, k_1E_f^{**} >0$$ and $$\tilde{\mathcal {R}}_{vs}^2 = \mathcal {R}_m\mathcal {R}_f$$, it follows that $$1-\tilde{\mathcal {R}}_{vs}^2\frac{S_m^{**}S_f^{**}}{S_m^*S_f^*} = 0$$, which means (D.19) can be simplified as

$$\begin{aligned} \Delta =&\left[ \lambda _{fm}^{**}G_mk_1k_2k_3\iota _{f1}\iota _{f3}(\mu +\xi _f) +\lambda _{mf}^{**}G_fe_1e_2e_3\iota _{m1}(\mu +\xi _m)\right] k_{10}k_{11}e_7\mu ^4\\&+\lambda _{fm}^{**}\lambda _{mf}^{**}G_mG_f >0. \end{aligned}$$

Since the determinant ($\Delta$) is positive, it follows that the system (D.5) has a unique solution, given by $\textbf{Z} = \textbf{0}$, which contradicts the assumption that $\textbf{Z}$ is not a zero vector.

**Case 2:** $$\omega \ne 0$$.

The objective here is to show that when $\omega \ne 0$, then Re$(\omega )\ge 0$ gives a contradiction. It is convenient to define

$$\begin{aligned} F(\omega ) = \min \{|1+F_i(\omega )|, ~i=1, 2, \cdots , 18\}. \end{aligned}$$

Then, it follows, by taking absolute values in (D.16), that:

D.20 $$\begin{aligned} F(\omega )|\textbf{Y}| \le H|\textbf{Y}|. \end{aligned}$$

It can be shown (since $\omega \ne 0$ and Re$(\omega )\ge 0$) that, in this case, $|1+F_i(\omega )| >1$ for all $i = 1, 2, \cdots , 18$, and therefore $F(\omega ) >1$. It can be seen from (D.18) and (D.20) (and noting (D.17)), that $F(\omega )|\textbf{Y}| \le H|\textbf{Y}| \le sH\tilde{\mathcal {E}}_{1s}^p = s\tilde{\mathcal {E}}_{1s}^p$, and hence $|\textbf{Y}| \le \frac{s}{F(\omega )}\tilde{\mathcal {E}}_{1s}^p$. Since $F(\omega ) >1$, we obtain a contradiction to the minimality of *s*. Thus, Re$(\omega ) <0$. This completes the proof. $\square$

## Proofs of Theorems 3.1 and 3.3

### Proof of Theorem 3.1

#### Proof

Consider the simplified model (3.1). Recall from Section 3 that the disease-free equilibrium (DFE) of the simplified model is given by:

$$\begin{aligned} \mathcal {E}_{0n} =(S_f^*,V_f^*,0,0,0,0,0,0,0,S_m^*,V_m^*,0,0,0,0,0,0,0), \end{aligned}$$

where, now,

E.1 $$\begin{aligned}&S_f^* = \frac{(1-q_f)\pi _f}{\xi _f + \mu }, \; V_{f}^* = \frac{\pi _f(\xi _f+\mu q_f)}{\mu (\xi _f+\mu )}, \nonumber \\&\quad S_m^* = \frac{(1-q_m)\pi _m}{\xi _m+\mu }, \; V_m^* = \frac{\pi _m(\xi _m+\mu q_m)}{\mu (\xi _m+\mu )}. \end{aligned}$$

The local asymptotic stability of $\mathcal {E}_{0n}$ is derived using the next generation operator method (Diekmann et al. 1990; Van den Driessche and Watmough 2002). The matrices *F* (for new infection terms near the DFE) and *V* (for linear transitions in the infected compartments) are given, respectively, by

$$ F_n =\left[ \begin{array}{c|c} \textbf{0}_{5 \times 5} & \textbf{e}_{1n}^T \textbf{f}_{2n} \\ \hline \textbf{e}_{1n}^T \textbf{f}_{1n} & \textbf{0}_{5 \times 5} \end{array}\right] \; \; \; \text { and } \; \; \; V_n =\left[ \begin{array}{c|c} V_{1n} & \textbf{0}_{5 \times 5} \\ \hline \textbf{0}_{5 \times 5} & V_{2n} \end{array}\right] ,$$

where,

$$\begin{aligned} \textbf{e}_{1n}&= (1,0,0,0,0),\; \textbf{f}_{1n} = \frac{\beta _{fm} c_{mf} p_m^*}{N_m^*} (\eta _{f}, 1, \tilde{\theta }_{f}, \tilde{\theta }_{f} \tilde{\kappa }_{f}, 0),\\&\quad \textbf{f}_{2n} = \frac{\beta _{m f} c_{mf} p_f^*}{N^*_m} (\eta _m, 1, \tilde{\theta }_m, \tilde{\theta }_m \tilde{\kappa }_m, 0),\\ V_{1n}&= \begin{bmatrix} \sigma _f + \mu & 0 & 0 & 0 & 0 \\ -\sigma _f & \psi _f + \mu & 0 & 0 & 0 \\ 0 & -(1-b_f)\psi _f & \alpha _f + \mu & 0 & 0\\ 0 & 0 & -(1-k_f)\alpha _f & \tilde{g}_f+\mu & 0 \\ 0 & 0 & 0 & (1-d_f)\tilde{g}_f & r_f+\tilde{\delta }+\mu \end{bmatrix}, \\ V_{2n}&= \begin{bmatrix} \sigma _m + \mu & 0 & 0 & 0 & 0 \\ -\sigma _m & \psi _m + \mu & 0 & 0 & 0 \\ 0 & -(1-b_m)\psi _m & \alpha _m + \mu & 0 & 0\\ 0 & 0 & -(1-k_m)\alpha _m & \tilde{g}_m+\mu & 0 \\ 0 & 0 & 0 & (1-\tilde{d}_m)\tilde{g}_m & r_m+\mu \end{bmatrix}, \end{aligned}$$

with, $p_f^* = S_f^* + (1-\varepsilon _v) V_f^* = \frac{\pi _f}{\mu } \bigg ( 1-\varepsilon _v \; \frac{\xi _f + \mu q_f}{\xi _f + \mu }\bigg )$ and $p_m^* = S_m^* + (1-\varepsilon _v) V_m^* =\frac{\pi _m}{\mu } \bigg ( 1-\varepsilon _v \; \frac{\xi _m + \mu q_m}{\xi _m + \mu }\bigg ).$ It is convenient to define the quantity:

$$\begin{aligned} \mathcal {R}_{vn}= \rho (F_nV_n^{-1}) = \sqrt{\mathcal {R}_{fn} \mathcal {R}_{mn}}, \end{aligned}$$

where,

$$\begin{aligned}&\mathcal {R}_{fn} = \frac{\beta _{f m} c_{mf} \mu p_m^*}{\pi _m (\sigma _f + \mu )}\\&\quad \left[ \eta _{f} + \frac{\sigma _f}{\psi _f + \mu } + \frac{\tilde{\theta }_{f} \sigma _f (1-b_f)\psi _f }{(\psi _f + \mu )(\alpha _f + \mu )} +\frac{\tilde{\theta }_{f} \tilde{\kappa }_{f}\sigma _f (1-b_f)\psi _f (1-k_f)\alpha _f}{(\psi _f + \mu )(\alpha _f + \mu )(\tilde{g}_f+\mu )}\right] , \\&\mathcal {R}_{mn} = \frac{\beta _{m f} c_{mf} \mu p_f^*}{\pi _m (\sigma _m + \mu )}\\&\quad \left[ \eta _m + \frac{\sigma _m}{\psi _m + \mu } + \frac{\tilde{\theta }_m \sigma _m (1-b_m)\psi _m }{(\psi _m + \mu )(\alpha _m + \mu )} +\frac{\tilde{\theta }_m \tilde{\kappa }_m \sigma _m (1-b_m)\psi _m (1-k_m)\alpha _m}{(\psi _m + \mu )(\alpha _m + \mu )(\tilde{g}_m+\mu )}\right] . \end{aligned}$$

It follows from Theorem 2 of (Van den Driessche and Watmough 2002) that the DFE, $\mathcal {E}_{0n}$, of the simplified model (3.1) is locally-asymptotically stable if $\mathcal {R}_{vn}<1$, and unstable if $\mathcal {R}_{vn}>1$. $\square$

### Proof of Theorem 3.3

#### Proof

Consider the special case of the simplified model (3.1) with no disease-induced mortality (i.e., $\tilde{\delta } = 0$); so that $N_i(t)\rightarrow \frac{\pi _i}{\mu }$ as $t\rightarrow \infty$ for $i=f,m$. Let $\mathcal {R}_{vn}<1$. It is convenient to define $W_i(t) = \frac{S_i(t) +(1-\varepsilon _v) V_i(t)}{\pi _i/\mu }$ and $W_i^* = \frac{S_i^* +(1-\varepsilon _v) V_i^*}{\pi _i/\mu },$ for $i = f, m$. It can be seen from the bounds (3.5) and (3.6) that

E.2 $$\begin{aligned} W_i(t)\le W_i^* \;\;\; \text {for all} \;\;\; t\ge 0 \;\;\; \text {in} \;\;\; \mathcal {D}^*. \end{aligned}$$

Furthermore, let $\textbf{x}(t) = (E_f(t), I_f(t), P_f(t), Q_f(t), C_f(t), E_m(t), I_m(t), P_m(t), Q_m(t), C_m(t))^T$ be the vector of the infected compartments of this special case of the simplified model (3.1). The equation for the rate of change of $\textbf{x}(t)$ can then be re-written in terms of the next generation matrices $F_n$ and $V_n^*:= V_n|_{\tilde{\delta }=0}$ (with $F_n$ and $V_n$ matrices given in Section 3) as:

E.3 $$\begin{aligned} \dot{\textbf{x}}(t) = (F_n-V_n^*)\textbf{x}(t) - J_n \textbf{x}(t), \end{aligned}$$

where,

$$\begin{aligned} J_n = \left[ \begin{array}{c|c} \textbf{0}_{5 \times 5} & \textbf{e}_{1n}^T ~\textbf{j}_{2n} \\ \hline \textbf{e}_{1n}^T ~\textbf{j}_{1n} & \textbf{0}_{5 \times 5} \end{array}\right] , \end{aligned}$$

with, $\textbf{e}_{1n} = (1,0,0,0,0), \; \textbf{j}_{1n} = \beta _{fm} c_{mf} (W_m^*-W_m)(\eta _{f}, 1, \tilde{\theta }_{f}, \tilde{\theta }_{f} \tilde{\kappa }_{f}, 0),$ and $\textbf{j}_{2n} = \beta _{m f} c_{mf}(W_f^*-W_f)(\eta _m, 1, \tilde{\theta }_m, \tilde{\theta }_m \tilde{\kappa }_m, 0).$ Since $\textbf{j}_{1n}$ and $\textbf{j}_{2n}$ are non-negative vectors (from (E.2)), it follows that $J_n$ is a non-negative matrix. Consequently, equation (E.3) can be written in terms of the following inequality:

E.4 $$\begin{aligned} \dot{\textbf{x}}(t) \le (F_n-V_n^*)\textbf{x}(t). \end{aligned}$$

Consider the linear ODE system given by the equality $\dot{\textbf{x}}(t) = (F_n-V_n^*)\textbf{x}(t)$ in (E.4). If $\mathcal {R}_{vn}<1$, then $\rho (F_n{V_n^*}^{-1})<1$ (based on the local asymptotic stability result given in Theorem 3.1), which is equivalent to all eigenvalues of the Jacobian, $F_n-V_n^*$, of the linear system $\dot{\textbf{x}}(t) = (F_n-V_n^*)\textbf{x}(t)$, having negative real parts (Van den Driessche and Watmough 2002). Hence, the linearized differential inequality (E.4) is stable whenever $\mathcal {R}_{vn}<1$. Thus, it follows, by the standard comparison theorem (Lakshmikantham et al. 2016; Smith and Waltman 1995), that $\textbf{x}(t) \rightarrow \textbf{0}$ as $t\rightarrow \infty .$ Substituting $\textbf{x}(t)=\textbf{0}$ into the equations of the special case of the simplified model (3.1) shows that $S_i(t)\rightarrow S_i^*$ and $V_i(t) \rightarrow V_i^*$, as $t\rightarrow \infty$, for $i = f, m$. Hence, the disease-free equilibrium ($\tilde{\mathcal {E}}_{0n}$) of the special case of the simplified model (3.1) with negligible disease-induced mortality (i.e., $\tilde{\delta }=0$) is globally-asymptotically stable in $\mathcal {D}^*$, whenever $\mathcal {R}_{vn}<1$. $\square$
